# Assessing and Enhancing Movement Quality Using Wearables and Consumer Technologies: Thematic Analysis of Expert Perspectives

**DOI:** 10.2196/56784

**Published:** 2024-09-13

**Authors:** T Alexander Swain, Melitta A McNarry, Kelly A Mackintosh

**Affiliations:** 1 Applied Sports, Technology, Exercise and Medicine (A-STEM) Research Centre Swansea University Swansea United Kingdom

**Keywords:** physical activity, exercise, wellness, qualitative, sensors, motor skill, motor learning, movement skills, skill development, movement assessment

## Abstract

**Background:**

Improvements in movement quality (ie, how well an individual moves) facilitate increases in movement quantity, subsequently improving general health and quality of life. Wearable technology offers a convenient, affordable means of measuring and assessing movement quality for the general population, while technology more broadly can provide constructive feedback through various modalities. Considering the perspectives of professionals involved in the development and implementation of technology helps translate user needs into effective strategies for the optimal application of consumer technologies to enhance movement quality.

**Objective:**

This study aimed to obtain the opinions of wearable technology experts regarding the use of wearable devices to measure movement quality and provide feedback. A secondary objective was to determine potential strategies for integrating preferred assessment and feedback characteristics into a technology-based movement quality intervention for the general, recreationally active population.

**Methods:**

Semistructured interviews were conducted with 12 participants (age: mean 42, SD 9 years; 5 males) between August and September 2022 using a predetermined interview schedule. Participants were categorized based on their professional roles: commercial (n=4) and research and development (R&D; n=8). All participants had experience in the development or application of wearable technology for sports, exercise, and wellness. The verbatim interview transcripts were analyzed using reflexive thematic analysis in QSR NVivo (release 1.7), resulting in the identification of overarching themes and subthemes.

**Results:**

Three main themes were generated as follows: (1) “Grab and Go,” (2) “Adjust and Adapt,” and (3) “Visualize and Feedback.” Participants emphasized the importance of convenience to enhance user engagement when using wearables to collect movement data. However, it was suggested that users would tolerate minor inconveniences if the benefits were perceived as valuable. Simple, easily interpretable feedback was recommended to accommodate diverse audiences and aid understanding of their movement quality, while avoiding excessive detail was advised to prevent overload, which could deter users. Adaptability was endorsed to accommodate progressions in user movement quality, and customizable systems were advocated to offer variety, thereby increasing user interest and engagement. The findings indicate that visual feedback representative of the user (ie, an avatar) should be used, supplemented with concise text or audible instructions to form a comprehensive, multimodal feedback system.

**Conclusions:**

The study provides insights from wearable technology experts on the use of consumer technologies for enhancing movement quality. The findings recommend the prioritization of user convenience and simplistic, multimodal feedback centered around visualizations, and an adaptable system suitable for a diverse audience. Emphasizing individualized feedback and user-centric design, this study provides valuable findings around the use of wearables and other consumer technologies to enhance movement quality among the general population. These findings, in conjunction with those of future research into user perspectives, should be applied in practical settings to evaluate their effectiveness in enhancing movement quality.

## Introduction

Wearable technology is ubiquitous in the modern era, enabling behavioral and physiological tracking of numerous variables, including the 24-hour movement profile [1,2]. However, commercially available wearable devices have almost exclusively focused on the quantification of such measurements, largely overlooking the emerging capabilities of many devices to assess, and potentially improve, human movement quality [1-3]. While there is a vast array of evidence supporting the need to be physically active for overall health and well-being [4-6], research has also demonstrated the physiological and cognitive benefits associated with improved motor competence [7]. Indeed, better movement quality has a catalytic effect on facilitating lifelong physically active lifestyles [7,8].

In elite sports, movement quality is widely analyzed as athletes strive to improve performance [9]. Yet, there is a dearth of opportunities for the wider population, encompassing youths through to older adults, to capitalize on either the health or performance benefits of better movement quality. Indeed, the most common means of assessing movement quality, such as optical motion capture [10], the use of depth cameras [11,12], or the employment of a coach [13], have associated practical and financial limitations [10,11,13]. However, wearable technology may help to overcome these barriers [13]. Wearable devices, equipped with motion-detecting sensors such as accelerometers, have the capability to capture movement data. These components can be leveraged to assist in assessing and improving an individual’s movement quality during specific activities [14]. As the capabilities of wearable technology continue to improve, it is important to identify how such devices can best be implemented in the general, nonelite, population. Furthermore, it is important not only to facilitate user-friendly means of collecting data but also to optimally deliver specific and contextualized feedback, using smart technology applications [14], to maximize the improvements in movement quality [2,15].

A growing body of scientific literature has shown the capabilities of wearable devices to detect and classify movement discrepancies. Specifically, wearable devices have been frequently validated for use in the assessment of movement quality in clinical contexts [16-18], as well as for specific sports and exercises [19-24]. Kianifar et al [16], for example, demonstrated that a binary machine-learning algorithm could distinguish between “good” and “poor” repetitions of a single-leg squat with 90% accuracy using a single wearable device worn on the ankle, and 96% accuracy using 3 devices (ankle, thigh, and lower back). Moreover, O’Reilly et al [22-24] classified specific movement discrepancies using a network of 5 sensors, with accuracies of 78%, 80%, and 70% for the barbell deadlift, body weight squat, and body weight lunge, respectively. However, the delivery of feedback is seldom considered within the scope of such validation studies [17,22-25] or is expected to be provided by a trained clinician [16].

The perspectives of influential figures, such as parents [26], teachers [27], and health professionals [28,29] have been explored in the context of wearable-based physical activity tracking. More prevalently, however, user perceptions around the practicalities and limitations of wearable activity trackers have been researched [30-33]. Collectively, these studies highlight several common barriers to their use: device unreliability and an associated lack of trust, a prerequisite for technological literacy, and age-related usability challenges for both young children and older adults due to physical constraints. Additionally, user opinions around physical activity feedback have been explored extensively, specifically in relation to how much physical activity an individual has performed [27,34,35], which is important to maximize user engagement [35].

Consumer perspectives play a valuable role in identifying user needs, particularly in the preliminary stages of product development, recognizing that designers and developers may overlook requirements that are specific to the user demographic [36]. However, relying solely on consumer insights is limiting, as users are typically constrained by their current mental models; they lack the ability to foresee disruptive context changes, such as technological advancements, and opportunities for novel innovations [37]. Moreover, it is reasonable to assume that consumers lack the comprehensive understanding of critical aspects of product development that designers and developers possess, particularly regarding technological and manufacturing capabilities. Consequently, it is beneficial to implement synergistic design strategies that use the insights of consumers, along with the expertise, insights, and interpretive qualities of developers and individuals in consumer-facing positions [37,38]. Yet, to date, there is a notable gap in the literature regarding the perceptions of individuals who have worked directly in the development and application of wearable technology. Furthermore, there is also a dearth of formative research that considers the practicalities of wearables and the provision of technology-based feedback specifically for assessing the quality of human movement (ie, how well individual moves) among the general population. Therefore, the aim of this study was to ascertain the opinions of experts with combined experience in the development and application of wearable technology, with a focus on its application for measuring movement quality and providing feedback. Additionally, it sought to identify potential strategies for incorporating the participants’ preferred assessment and feedback characteristics into a technology-based intervention for the general, recreationally active population. This study represents the first of two, with the second focusing on consumer opinions, seeking to provide a comprehensive, holistic perspective on the use of consumer technologies for assessing and enhancing movement quality.

## Methods

### Data Collection and Analysis

A total of 12 adults (age: mean 42, SD 9 years; 5 males) were recruited via an intraorganizational email network. All participants had extensive experience in the development or application of technology for sports, exercise, and wellness. Purposive sampling was used to ensure a diverse and balanced range of specialists from both commercial and product development roles were selected from across an organization. Although data saturation is commonly used to ensure an adequate sample size within qualitative research [39], it was not conducive to the analytical approach used in this study [40]. Therefore, the pragmatic guiding concept of information power was instead applied to appraise and confirm the adequacy of the final sample size based on the focused study aims, a dense intrainstitutional sample, and theory-guided investigation methods [41].

Semistructured interviews were conducted primarily in-person at the participants’ workplace by the lead researcher (TAS), with 1 interview conducted via Microsoft Teams (version 1.5.00.21463). All interviews took place between August 18, 2022, and September 8, 2022. For each interview, only TAS and the interviewee were present. The interviews lasted 38 (SD 16) minutes and used a predetermined interview schedule that enabled follow-up questions and prompts (Multimedia Appendix 1). Participants were also presented with an information sheet outlining the overarching rationale for the study and their participation (Multimedia Appendix 2). All interviews were conducted in English with fluent, nonnative English speakers. The initial questions aimed to stimulate freethinking. However, as the discussion progressed, the questions became more targeted and contextualized. When discussing visual feedback, examples were used as prompts to direct participants and aid understanding (Figures S1-S3 in Multimedia Appendix 1). The examples were selected to indicate ways in which visualizations have been previously used, incorporating a combination of technicality, simplicity, and an array of designs. The prompts provided context and offered an opportunity for reflection, while enabling participants to express their opinions relating to certain features and characteristics of visualizations. The interview schedule was compiled by TAS and MAM, and subsequently approved by the wider research team. Interviews were audio recorded using a Philips DVT3400 Voice Tracer (Koninklijke Philips N.V.), and subsequently transcribed verbatim (Multimedia Appendix 3). To ensure anonymity, the characteristics of the 12 participants have been intentionally restricted. However, participants could be classified into 2 broader categories based on their job role: commercial (n=4), or research and development (R&D; n=8). Consequently, participants with a commercial function have been assigned identification codes C1-C4, while those in R&D have been designated as RD1-RD8.

The interviews were analyzed by using the 6-stage reflexive thematic analysis (RTA) process developed by Braun and Clark [42-45]. This method was used to identify repeated patterns in the data, organized around particular themes [43]. The use of RTA is conducive to exploring deeper, underlying meanings within the data, rather than superficially identifying, and reporting, what participants said [43]. Additionally, RTA offered a structure to which the analysis could adhere while allowing themes to develop organically without undue restriction [43]. Initial familiarization with the data involved relistening to the audio recordings of each interview while taking notes and compiling a summary report for each of the 12 interviews [43-45], comparable with the approach taken by Byrne [46]. The audio transcriptions were uploaded to NVivo (release 1.7; QSR International), where TAS generated the initial codes before undertaking a period of refinement and organization. Themes and subthemes were then iteratively created, assisted by thematic mapping (Multimedia Appendix 4). The initial codes were developed inductively without any predetermined coding framework, though, as is typical in RTA, a degree of deductive analysis was required to ensure that the included codes and themes were related to the overarching research direction [42,44,46]. Notably, by using both inductive and deductive methods, the research questions were iteratively refined throughout the 6-stages to ensure relevance to the intended application. Both semantic and latent interpretations of the data were investigated [43,44,46].

### Philosophical and Theoretical Underpinnings

The study provided a platform for wearable technology experts to share their professional knowledge and personal experiences, providing valuable information related to improving movement quality and allowing consumer demands and future development opportunities to be explored. Questions regarding movement-quality feedback were theoretically underpinned by Schmidt and Wrisberg [15] and Fleming and Mills [47]. That is, recognizing individuals may respond better to certain sensory modalities than others [47], and that motor skill–learning and –retention can be maximized through optimal delivery of feedback, though this is both individual and contextual [15]. However, participants were afforded the freedom to think creatively with minimal restrictions when discussing data collection.

### Researcher Positionality

Reflexivity implies that there is an inherent, yet valued, subjective component to RTA [42]. As such, it is important to understand how researcher positionality may have influenced the findings of the study [42,48]. The lead researcher (TAS) is a White British male who is a current PhD candidate and holds a BEng in Mechanical Engineering and an MSc by Research in Sport and Exercise Science. TAS also has extensive industrial experience, albeit in industries largely unrelated to wearable technology. As such, these experiences were influential during the analysis, where considerations around potential applications and future developments were, at times, central to the thought process. Prior to commencing this study, TAS was already familiar with wearable technology, both as a researcher and as a consumer. Further, TAS had briefly met with some of the participants ahead of the interviews.

### Methodological Rigor

To increase credibility and to ensure methodological rigor, the data analysis process was discussed with KAM, and the generated themes were checked to ensure they appropriately represented the data [29]. Additionally, a “critical friend” used the checklist devised by Braun and Clark [45] to evaluate the quality of the thematic analysis [48]. Any unsatisfactory responses to the checklist items were highlighted and feedback provided, after which amendments were made until all questions in the checklist were adequately answered. The COREQ (Consolidated Criteria for Reporting Qualitative Research) checklist [49], an established method of ensuring explicit and comprehensive reporting qualitative research, was also used (Multimedia Appendix 5)

### Ethical Considerations

This study was approved by Swansea University College of Engineering Research Ethics and Governance Committee (TS_01-07-22). All study data were anonymized before analysis and no other personal or private information was included in the data set to protect the privacy and confidentiality of the study participants. All participants provided written informed consent before participation.

## Results

**Figure 1:**
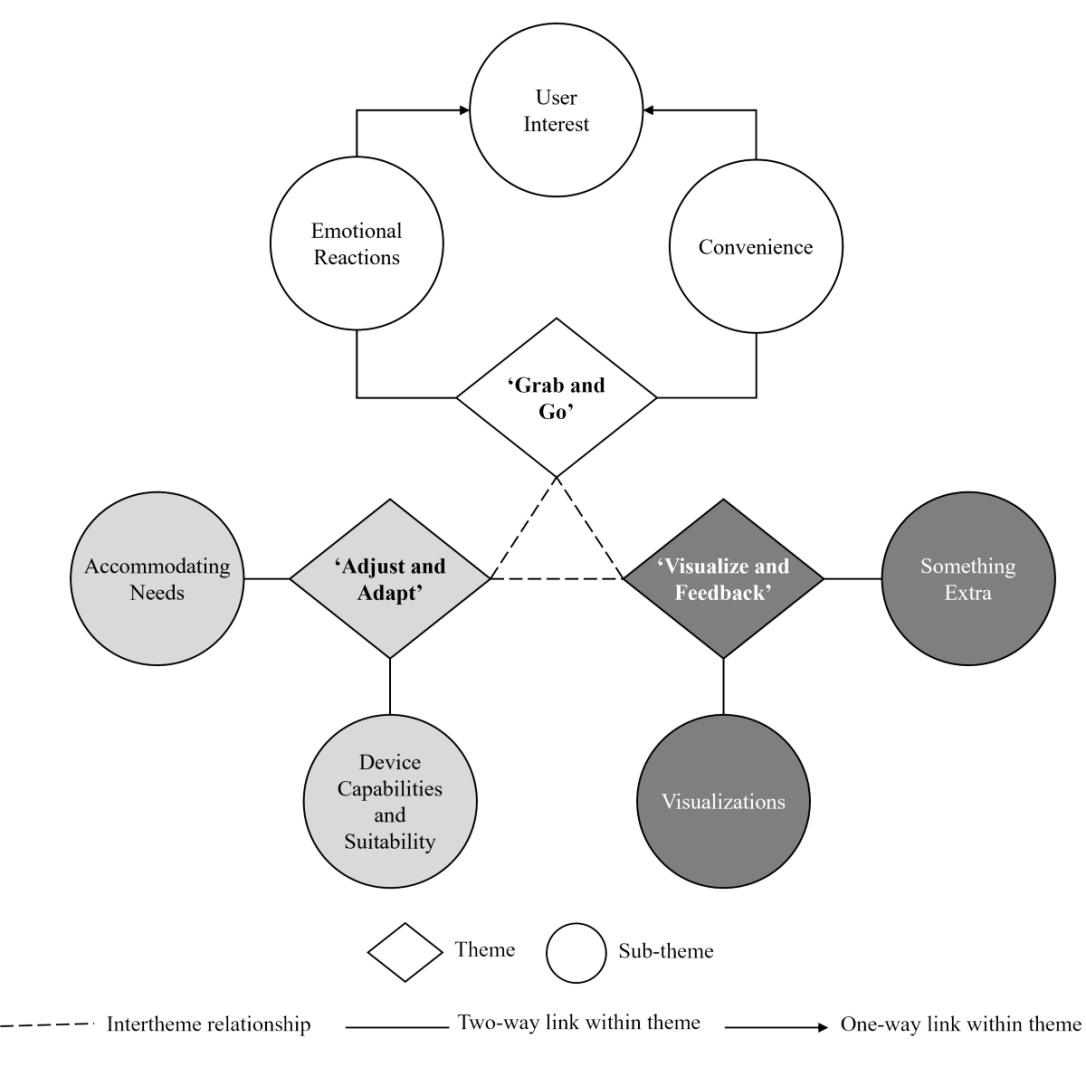
Final thematic map illustrating the interrelatedness of 3 generated themes: “Grab and Go,” “Adjust and Adapt,” and “Visualize and Feedback.” Within themes, the relationships of the identified subthemes are also presented, including directionality.

Overall, three key themes were generated as follows: (1) “Grab and Go,” (2) “Adjust and Adapt,” and (3) “Visualize and Feedback,” with each theme featured alongside the subthemes and associated relationships (Figure 1).

### Grab and Go

#### User Interest

User interest was identified as a key determinant of wearable technology use. The experts explained the need to capture user interest through functional hardware but also highlighted the need for a device that “looks cool, nice” (RD2), or even perhaps discrete devices that are “super comfortable” (RD2) and that “others wouldn’t be able to see so clearly that you’re wearing some kind of device” (RD6).

Participants also described the importance of users being aware of the need for movement-quality feedback and the associated benefits of good movement, proposing that technology use could promote this. The interviewees suggested ways to encourage the implementation of technology in the first instance, highlighting that “if they will get really precious information” (RD5) and “if the information is good enough” (RD3), users will be more receptive to wearing devices and receiving feedback. However, the experts largely focused on the various benefits of the technology, especially for “the beginners (...) or those who haven’t been physically active” (C3), who may lack understanding of good movement quality. It was suggested that such individuals may have “a common fear, in a way, that [they] are doing this movement wrong, and [they] might injure [themselves]” (RD8), hence streamlined feedback delivery would be welcomed.

Variety was proposed as a means of retaining user interest, with 1 participant who had used wearable technology personally stating that they “got bored of [visualizations] because they never changed” (RD2). It was also felt that the implementation of a progression-based system would be beneficial when learning to move proficiently.

I think it could be good to have the basics. “This is the basics”, and then when you've, let's say, you've done that exercise on five different occasions, and then you can make the assumption that “Okay, now you're familiar with these three things”, that it tells you, then you could move on to the next three things.

C4

However, it is important to recognize that progression may fluctuate and that regression in skill proficiency may also occur. Hence, an adaptable system that accommodates both advancements and potential regressions would likely be more appropriate. Additionally, where feedback was considered for use in movement skill learning, it was proposed that the learning phase may have a finite lifespan and that the feedback would no longer be required once the skill mastery had been achieved.

So, using the app like that, with the pictures, with the squat, and things like that, it's kind of hard to imagine that somebody uses it for a long time.

C3

Participants widely spoke of the need to avoid information overload. Too much information was generally viewed as a barrier to technology use. When presented with an example of a technical visualization (Figure S1 in Multimedia Appendix 1), 1 interviewee stated, “I just can’t get a hold of it in any way, and I lost interest, like, in a second” (C1), whereas another proposed, “for the [sic] normal people, the simpler, the better” (C2), despite acknowledging that a niche demographic with greater experience or interest in the data may “want to dig into the details” (C2). Participants repeatedly advocated for simplicity, especially “if you are starting [exercise] from scratch” (C2), as it was speculated that it would make skill learning easier. However, 1 R&D participant also recommended simplicity for more advanced athletes to assist with the processing of information, where the feedback might be viewed as useful rather than a distraction, or something that could be perceived as intimidating.

The usability has to be, even if I'm a professional (...) in that moment I'm exercising and doing sports, I do not have brain cells to start analyzing what I'm doing. If I have the information when I'm on the bike, or during the physical movements. So, with one glance, I have to analyze it easily.

RD7

#### Convenience

The convenience of the applied technology and the delivery of feedback were highlighted as essential. Generally, the experts expressed a preference for a more streamlined process without unnecessary complexities or time delays between the data collection and the delivery of movement feedback. One R&D participant spoke of their own personal experiences to provide context around the benefits of convenient movement-quality feedback.

I don't want to go on the classes. I'm just too lazy to join the one-month course or something like that. If somebody could offer me a device for every now and then, put it on and then giving me feedback of my swimming technique. How to make it more effortless. That would be interesting.

RD2

To accompany the desire for simplicity, the wearable technology experts widely alluded to consumer appetite for a convenient solution, promoting the use of wearable technology for data collection, as it “collects everything automatically, so you don’t need to collect it by yourself” (RD1) and it is “easy because it’s always with you” (RD5). However, when discussing the use of technology for the provision of feedback, opinions were mixed. Smartphones were broadly viewed as the preferred means of receiving feedback, “because everybody has one, and it’s always there” (RD2), and “computers are too big” (RD4). Smartphones are more prevalent in modern society and have a wide range of capabilities, whereas other larger devices are too impractical. Yet, some participants highlighted the negative aspects of smartphones for feedback. For example, a frequent gym-goer stated “During my exercise, I don't want to gaze [at] it all the time (...) so mobile phone, no thank you.” (RD7)

Limitations and opportunities were simultaneously identified for other technologies too, both for collecting data and providing feedback. Wrist units were perceived as convenient for most daily activities and many sport and exercise applications, especially as they can be used to collect data and subsequently deliver feedback. But the wrist was also viewed as “a very competitive spot” (RD2) that may already be being used for other devices or accessories. Additionally, participants expressed concern that a single wearable might be “guessing” (RD1) when used on its own for data collection, and may also be inappropriate for some sports, as it may be “harder to move, or it might even ruin your technique” (RD3). Similarly, a superficial device could be a problem “like, if you have contact sports” (RD8), as they could increase injury risk.

There appeared to be a trend whereby convenience could be sacrificed to an extent, albeit reluctantly, if greater benefits could be gleaned from, for example, a more comprehensive network of wearable devices. For instance, fewer sensors intuitively take less time to put on, but users may be required to wear more sensors to enhance the quality of the data and feedback. This may be easier to facilitate if user demands can be accommodated, such as if the sensors “allow some tolerance of where they are” (RD8) and “they don’t really affect your performance in a bad way” (RD3). Furthermore, by accommodating accessible and favorable sensor locations, ensuring “that it’s easy to wear” (RD7), they do not “require quite a lot of time to strap on” (RD8), and even making devices multifunctional, the experts appeared to suggest that much of the convenience could be retained.

Yeah, wrists are easy. I guess, legs or ankles could be possible as well. And torso...

RD8

But then, if it's, like, they need to buy it just for [capturing movement data], maybe that might be a problem. But, if they can use it for other purposes, too.

RD1

#### Emotional Reactions

This subtheme primarily centered around emotional reactions to feedback, rather than the data collection process. Participant C3 highlighted the skepticism some users may have when receiving feedback, possibly from a lack of familiarity with technology, but also due to a lack of trust.

For some people, I'm quite sure that it would be kind of hard to believe that, “Is this really working? Or is this good for me? And how do they know that I should do this?” and convincing them.

C3

The experts recognized that critiquing an individual’s movement quality may be a sensitive topic. One participant expressed that users often reside in what was described as an “an ideal self-bubble” (RD4), where individual beliefs differ greatly from reality. The participant indicated that individuals with a preconceived notion that they already understand good movement would react negatively to criticism. The interviewees indicated that product users would rather “trust the feeling [they] have in [their] body” (C1) and if the feedback was critical, they may perceive the feedback as erroneous, suggesting that they “wouldn’t have [it] again” (C1). It was also proposed that, when receiving feedback, some users could react badly to seeing themselves performing a movement.

Some people might find it, I don’t know, even embarrassing. Not everyone likes to see themselves, at least if they are not good at what they are doing.

RD1

Participants highlighted that a large portion of the population is technology-averse and that many of the population have a reluctance to engage with modern devices, particularly for health-related applications. It was felt that some people would find it “kind of hard to believe” (C3) what they are being told by a device and that they may also “be nervous about where the information is used” (RD2). However, if delivered effectively and positively, “feedback is rewarding” and could encourage users to persist with the activity.

It was believed that feedback “has to be really constructive” (C3), and supportive of progression, such that user engagement is retained for improving their movement quality, but also that they are not deterred from using the technology for other capabilities. Participants advised that feedback needs to inform consumers of any movement discrepancies, “but in order to give a positive impression, it would also need to have something encouraging” (C2). One individual cautioned of the effects of negatively perceived feedback.

Like, “I don't like this device, because it tells me stuff like this, and I don't understand what it means, and I don't want to use this anymore”. So, they might just drop their interests for every feature in the watch and not just that. So, it could also be kind of risky.

RD3

### Adjust and Adapt

#### Accommodating Needs

When discussing feedback, those interviewed provided conflicting views, particularly with regard to detail. Some experts suggested that beginners with less experience and understanding of good movement “might find it really useful to get some sort of explanation” (RD3) to help them understand how to use the feedback, whereas others indicated that too much detail could lead to confusion.

Those who we are trying to get physically active more, or they are just beginning, not too much information, because, well, I think they could get lost in there and it's kind of hard to understand it.

C3

However, it is plausible that additional information may have been falsely associated with complexity, and that experienced individuals might actually “want to dig into the details” (C2). Notably, the interviewed experts collectively recognized that individuals would have different requirements and desires, proposing that giving the user some control over the feedback could be effective, enabling them to “check the key points” (RD3) during or immediately after exercise, but providing the opportunity to “read the details if [they] have time” (RD3). Some participants proposed that “background information about the person” (C3) could be integrated to offer users “the opportunity to give feedback to the system” (RD4), an approach that could enhance the insights provided by the wearable-based system. This may also enable increased relevance and accuracy, consequently tailoring the system to better accommodate individual requirements.

#### Device Capabilities and Suitability

Despite user demands, it is important to acknowledge the capability and accessibility limitations of existing technologies. As wearable technology is still a developing area, participants recognized the opportunities for improvement, such as being “more precise than they are nowadays” (C3), but also highlighted some existing restrictions that could limit how effectively wearable technology could be used for collecting movement data.

I see a lot of, especially in the hardware technology side, a lot of challenges that we still need to overcome. And when I talk about these, I mean, things like the sensor and sizing, the price of it, the communication of the sensor, or the sensors between each other.

C3

Additionally, participants spoke of device suitability and effectiveness in certain environments and contexts, acknowledging, “it depends on the exercise type” (C3). Similarly, different feedback modalities were suggested to be better in some settings than others. For instance, “audio would be a bit difficult in the gym (...) but audio would be nice on a run” (C3). Similarly, visual feedback would be inappropriate for running, yet more suitable in a gym or at home.

### Visualize and Feedback

#### Visualizations

Visual feedback, which may consist of images, videos, or animations, was thought to be the most effective way “to deliver a lot of information” (C2) with the greatest efficiency and ease of understanding “instead of having to read stuff” (C2). Interviewees generally endorsed the use of a digital representation of the users, such as an avatar, as “you immediately get what the information is about” (C2), similar to that used in Figure S1 in Multimedia Appendix 1. Instead of something abstract that is “not you (...) it’s something totally different” (C2), the experts largely favored more direct, relatable feedback with very little analysis required.

Participants offered ways in which feedback could be optimized, perhaps through the inclusion of accompanying visual standards to aim for, such as seeing “the perfect image there, in a shadow” (RD3). An R&D participant proposed that an image of the “perfect” movement could be presented along with the user’s movement “maybe side-by-side, something like that” (RD6). However, some individuals emphasized the importance of appropriate visualization design, evident through their own misinterpretations of the examples presented. One participant stated, “I don't think it’s very clear, at least for me” (C3), highlighting the need for clarity in the visualizations.

#### Something Extra

The wearable technology experts appeared to favor visualizations. However, the consensus was that visualizations would be best accompanied by an additional form of feedback to aid interpretation, as “visual on its own is maybe not enough” (C4). It was thought that visualizations alone would be able to show good or poor movements, but it was advocated that “it would need text and/or audio, also” (C4) to provide some sort of explanation. The interviewees generally felt that visualizations can depict the movements well, but consumers would likely be left without instruction to rectify or enhance any movement discrepancies if there was an absence of an additional feedback modality. It was also noted that, in some settings, visualizations may not be the most appropriate form of feedback, and therefore it could be beneficial to have alternatives.

Well, I would think that if you have visuals, you have to have a big screen. So, there could be problems. So maybe audio...

RD5

## Discussion

### Principal Findings

This study sought the opinions of experts with combined experience in the development and application of wearable technology, with emphasis on its application for measuring movement quality and delivering feedback. Overall, three themes were generated as follows: (1) “Grab and Go,” (2) “Adjust and Adapt,” and (3) “Visualize and Feedback.” Despite some ambivalence surrounding device preferences, there was a collective agreement among participants on the importance and effectiveness of wearable devices to provide real-time, detailed movement-quality feedback. This uniform agreement emphasized the potential of wearables to improve user awareness and enhance physical activity.

This study highlighted numerous barriers, as well as facilitators, to using wearables and consumer technologies to assess and improve movement quality. Initial accessibility, coupled with the retention of user interest, was recognized as essential to encourage users to improve their movement quality. Congruent with prior research [31], the interviewees emphasized the need to capture user interest through effective wearable device design and capability, and then preserve interest through ease of application, variety, engaging feedback, and providing scope for progression. Notably, however, this study is framed around the assessment and improvement of movement quality, inherently dependent on process-focused “Knowledge of Performance” feedback, in contrast to the outcome-based “Knowledge of Results” feedback frequently used when evaluating movement quantity [15,50]. Further, motor-skill refinement is arguably a more iterative process than increasing physical activity volume, given the requirement for more nuanced and continual refinements and repeated exposure [51]. Consequently, this study offers a novel perspective on feedback in assessing movement quality, focusing on the opinions of wearable technology experts rather than user perspectives.

To bolster efficacy, there is potential to apply methods such as machine learning, by using captured movement data and user feedback to enable algorithmic updates and facilitate the ongoing learning and improvement of the user’s movement quality [22]. This aligns with the appetite for personalization during movement-quality assessments identified in this study. Personalization would enable the user to have control over feedback modalities, feedback timing, and the level of detail they wish to receive, while potentially contributing to increased assessment accuracy. Additionally, congruent with previous research [29,31,52,53], this study highlights the potential negative impact of information overload on user interest, underscoring the advantages of individualization: users have control of how much and when feedback is provided. Indeed, the wearable experts advocated for streamlined feedback, delivered in manageable and interpretable quantities, while minimizing overall information provided and catering to the individual’s requirements. This concurs with Orphanides and Nam [54], where the need for flexible feedback methods was highlighted given the difficulties for specific populations, such as young children and older adults, to interact with modern technologies.

The risk of overwhelming users with excessive details may be negated through the use of glanceable displays when providing real-time feedback during certain activities [27], as indicated by the findings of this study. Specifically, research has suggested that delivering varied feedback in short snippets via glanceable displays may increase engagement and motivation to be more physically active [27,55,56]. However, it is postulated that glanceable displays may be too limiting to provide adequate nuances for substantial motor-skill developments, but could be appropriate for minor technical adjustments and reinforcements in real time. The importance of ensuring that feedback is both constructive and positive to maximize user experience was emphasized by participants in this study, given the risk of negative experiences leading to device discontinuation [57]. Offering constructive feedback can enhance the potential of wearable technology to deliver effective solutions for those seeking to improve their movement quality [58]. Furthermore, in alignment with previous research [26], a different perspective on the use of physical activity trackers suggests that once the feedback is understood and, in the context of movement, mastery is attained, a feature may no longer be used. This alternative perspective may alleviate the pressure for sustained use of a feature, while potentially prompting the need for further progression in developing advanced motor skills. However, considering the potential for skill regression, it is crucial that features can be seamlessly reintroduced as required, helping users both regain and maintain movement skill proficiency.

While each device has inherent strengths and weaknesses, it may be advantageous to provide flexibility in device selection, given the diverse opinions observed in this study. Customization is already prevalent in physical activity tracking, where users can typically modify performance outcomes (eg, step-count targets and total daily energy expenditure), as well as feedback methods [59,60]. However, using wearable technology to measure and assess movement quality is still in its infancy and there are additional challenges to personalization and the provision of user-specific feedback, largely attributable to individuals’ unique anthropometrics and physical limitations (eg, injuries and mobility restrictions). Of concern, as outputs become increasingly segmented and specific, measurement accuracy has been shown to proportionately decline [16,22]. In accordance with previous research, this study highlighted that consumers may have difficulties trusting technology [31,52], and unreliable, invalid data would likely exacerbate this issue. However, Meyer et al [52] proposed that there is a trade-off between detailed, yet erroneous, outputs and losing user-specificity due to oversimplification. Providing relevant and specific feedback to the user can be attained without the need for excessive detail [52], and, as suggested in the findings of this study, avoiding information overload could enhance motor-skill learning by streamlining the necessary information into manageable and interpretable quantities.

The unavoidable compromise between sensor quantity and the capability to effectively measure and assess holistic movements is a limitation of wearable technology [3]. It is widely recognized, and expressed by participants within this study, that a network of sensors positioned around the body is generally superior for conducting movement-quality assessments for holistic movements, with provisions of greater accuracies and more insightful movement capture [3,16,22-24]. Indeed, within clinical environments especially, there is very little leniency in measurement accuracies [61]. Hence, in such settings, there may be value in the application of a comprehensive sensor network. However, the possible need for multiple sensors may reduce practicality, a potential barrier for some. The participants of this study conveyed that users may be more tolerant of reduced practicality if the feedback was perceived as valuable and informative. Moreover, reiterating previous observations [26], participants indicated that once users achieve mastery of a movement, they might cease using certain features. It is therefore plausible to suggest that the short-term inconvenience of managing multiple sensors could also be justified by the long-term benefits in motor-skill development. Subsequently, this raises questions around the optimal balance between practical application and sufficient accuracy for general population consumers, considerations which align with recent pedagogical research for the assessment of motor development in youths [62]. Further, the cost implications of a multisensor approach should not be overlooked. Although users may tolerate temporary discomfort for improved movement-quality assessments using wearables, the perceived value and affordability of using multiple sensors, particularly over a relatively short period, could impact their willingness to adopt such technologies [13].

Participants discussed numerous feedback methods (ie, text, audio, visual, and haptic), providing contextual examples of when they may best be implemented for assessing movement quality. However, a visual representation of the user was widely endorsed by the experts in lieu of something more abstract or minimalistic, particularly when learning a new movement. In the context of activity quantification, overly minimalistic visualizations have been shown to be less favorable [52], and this trend appears to also extend into movement-quality feedback. While abstract visualizations may be effective for learning both simple and complex motor skills, they may be perceived as boring after prolonged use, and challenging to apply to complex multidimensional movements in 3D space [63]. Participants also encouraged the use of multimodal feedback, such that the combination of methods could complement any discrepancies that would exist in unimodal feedback. Specifically, it was proposed that supplementing a visual representation of the user with clear and concise text or instructional audio would aid understanding. Indeed, previous research strongly supports this, by indicating that audiovisual feedback enhances motor learning more effectively for a single task compared with single-modality feedback [50,63]. Interestingly, however, participants in this study suggested that some users may dislike seeing themselves on a screen, especially if their movement is being critiqued. This sentiment presents a notable paradox; those who would benefit from corrective feedback the most may experience discomfort through self-observation. Therefore, it is surmised, based on the present results, that an avatar may be a suitable compromise that is both engaging and understandable, yet offers sensitivity, and could help avoid potential psychological discomfort. Specifically, the use of avatars has been shown to be effective in supporting the learning of gross motor skills [64,65], and increasing physical activity levels [65].

### Strengths and Limitations

There are numerous strengths associated with this study, including the recruitment of participants with extensive experience in wearable technology use in sports, exercise, and wellness. The sample encompassed both technical expertise and insights from those who interact with users. Furthermore, the study used a thorough and rigorous data analysis process centered around RTA [42-45], a well-established and widely recognized qualitative research method. Nonetheless, the study is not without limitations. The sample size was identified to be adequate using the concept of information power in lieu of data saturation, which, according to Braun and Clark [45], is too subjective to find a definitive point of saturation when conducting RTA [40]. However, the interviews were conducted with experts from a limited recruitment pool, which may have narrowed the findings due to common experiences. As such, future research incorporating a more diverse, wider-reaching array of wearable technology experts is required, while it would also be beneficial to seek insights from prospective wearable users for evaluating movement quality specifically. Moreover, it is pertinent to note that this study was conducted with a view to targeting a nonelite demographic. Therefore, future research is warranted to consider feedback mechanisms for elite athletes.

### Conclusions

Overall, this study identified that wearable technology experts perceived convenience and simplicity as priorities for both movement data capture and feedback mechanisms. Further, due to the subjective demands of prospective users, an adaptable solution was considered preferable when implementing these findings in a practical setting. Moreover, it was advised that movement-quality interventions utilizing technology should be progressive and use visual feedback that is representative of the user, such as an avatar, supplemented with concise text or verbal instructions as part of a multimodal system. A second study will consider the opinions of prospective consumers to compare with the findings of this study and enable a comprehensive evaluation across all stakeholders. Thereafter, the combined findings from both wearable technology experts and users should be applied in a practical setting to assess their efficacy for enhancing movement quality.

## Appendix

Multimedia Appendix 1 Interview schedule.

Multimedia Appendix 2 Participant information sheets.

Multimedia Appendix 3 Transcripts.

Multimedia Appendix 4 Thematic maps to assist in the generation of themes.

Multimedia Appendix 5 ISSM COREQ (Consolidated Criteria for Reporting Qualitative Research) checklist.

## Abbreviations

COREQ: Consolidated Criteria for Reporting Qualitative Research
R&D: Research and Development
RTA: reflexive thematic analysis

## Data Availability

All data generated or analyzed during this study are included in this published article and its supplementary information files.
