# Supplementary material for: Assessing and Enhancing Movement Quality Using Wearables and Consumer Technologies: Thematic Analysis of Expert Perspectives
Source: JMIR Form Res. 2024 Sep 13;8:e56784. doi: 10.2196/56784 (PMC11437222; doi:10.2196/56784)
Supplement: Multimedia Appendix 1 [file formative_v8i1e56784_app1.doc]

**Supplementary Information 1 – Interview schedule**

*General introduction about there being no right or wrong answers, just really keen to get their perspectives and thoughts. All will be confidential and anonymous. Then a brief introduction into what we’re doing.*

**Icebreaker:**

*“Before I get started with the questions, I would like to get to know a little about you. So, if you are able to introduce yourself, tell me a little bit about your role, and what you like to do in your spare time”*

Ok, so now I want us to talk a bit about physical activity (reference if they’ve already spoken about it in icebreaker).

1. Are you physically active in your spare time?
2. How do you interpret the term ‘movement quality’?
3. What sorts of things help people to move well?
   1. During everyday life?
   2. When doing exercise?
4. What do you think influences how well people move?
   1. During everyday life?
   2. During exercise?
5. What benefits do you think people could have by improving how well they move during exercise?
6. What kind of information do you think is important to receive when trying to improve how well you move during exercise?

We are exploring ways to help people understand how they move and how they can improve the way they move during exercise. Currently, there are no affordable, accessible and effective ways in which this can be achieved. However, with the use of wearable technology, we hope to change this. We are also exploring ways that makes the data easy for people to interpret such that they can implement it safely and effectively.

1. Are you able to give me some examples of feedback methods you are aware of that help inform people of their activity?
   1. For measuring how much activity?
   2. For measuring how people move?
2. We have been really drawn to the use of visual aids.
   1. What do you understand by this? (*Interviewer to explain as required*)
   2. What do you think people would think of this?
   3. Would you like something like this – why/why not?
   4. Can you think of any examples of visualisations used to help people during exercise?
   5. Do you think this sort of feedback would be best provided in isolation, or with other forms of feedback, like audio cues, for example.
3. What sort of devices do you think would best provide feedback? (Prompts: App, watch, laptop)
4. How much detail would you expect feedback to provide.

Here’s a few examples of visualisations that exist in products to date, most of which are used to quantify activity, but also some which consider movement quality.

**
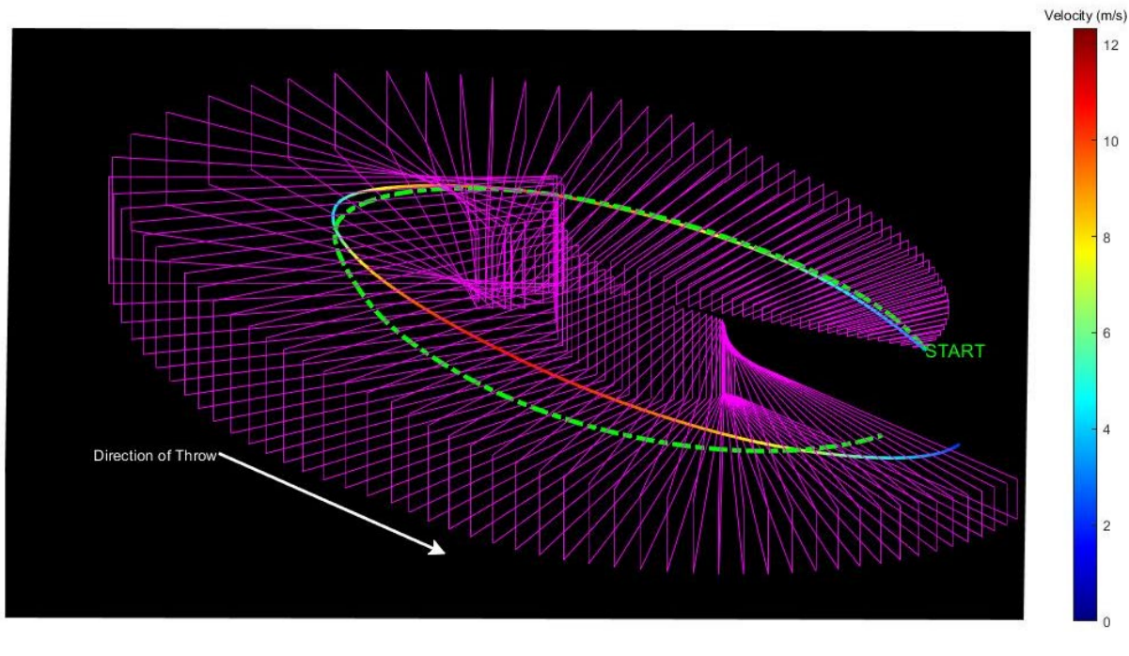
**

Gold-standard throw trace

Participant’s throw trace which also shows velocity

Band that indicates the tolerable amount of error

**Figure S1: A visualisation showing the wrist trajectory during an overhand throw compared against a gold standard throw**

1. What do you think about this *(consider things that you like/dislike)*? _______________________
2. Could you suggest how this could be improved? _______________________

**
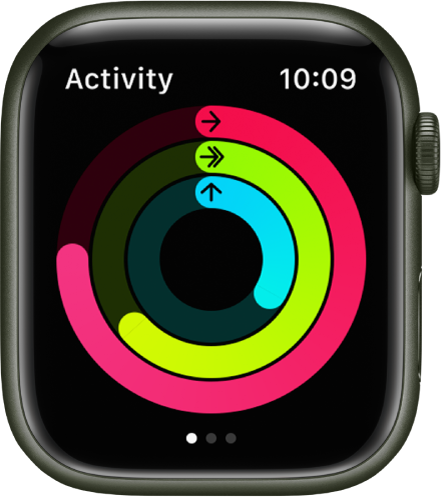
**

**Figure S2: Apple Watch rings indicating calories burned (red), time spent being active (green), and time spent standing (blue; from apple.com)**

1. What do you think about this *(consider things that you like/dislike)*? _______________________
2. Could you suggest how this could possibly be improved? _______________________

**
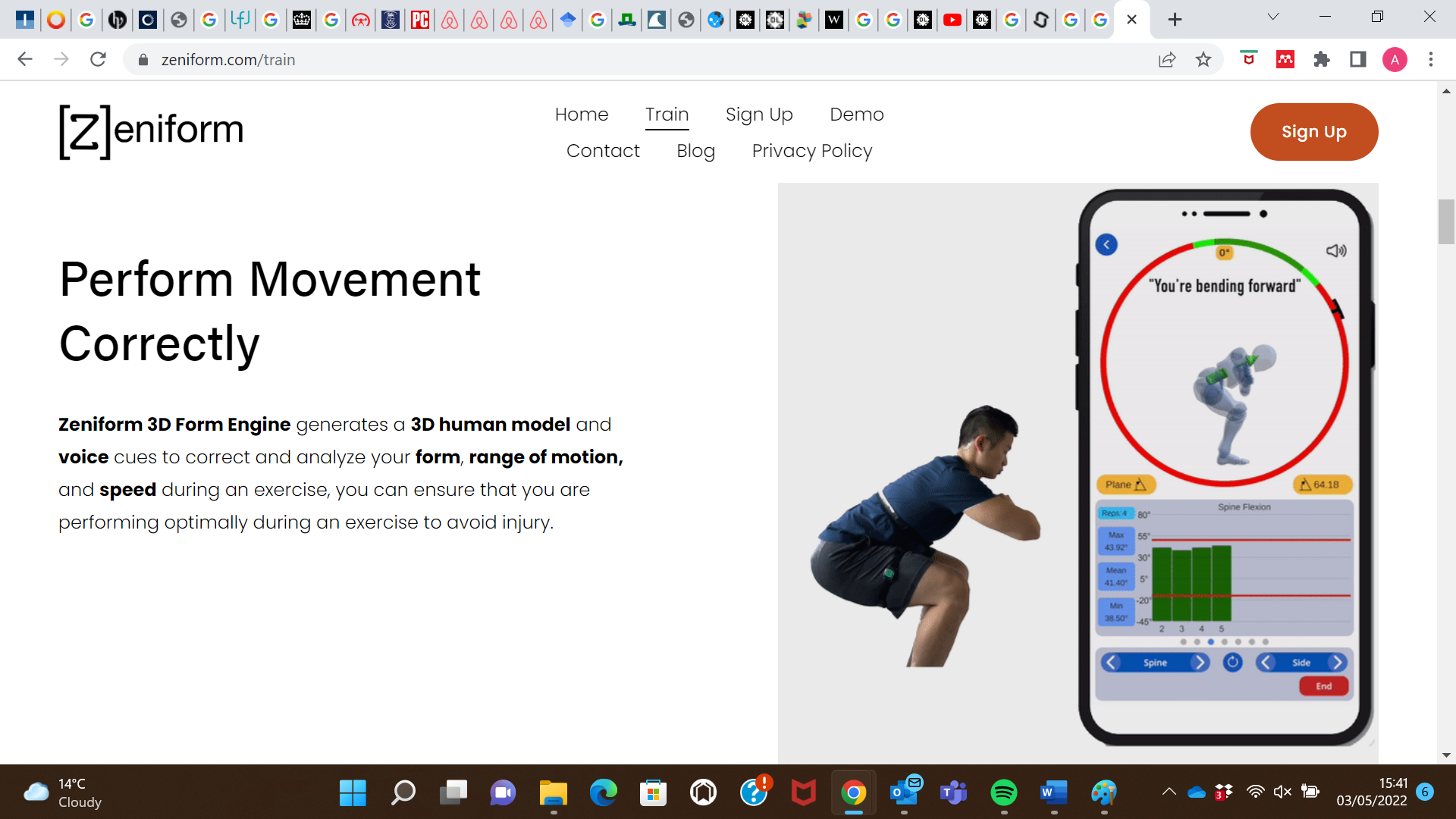
**

**Figure S3: A complete movement assessment app integrated with a network of wearable sensors providing real-time feedback as animations, images, text, and audio (from zeniform.com)**

1. What do you think about this *(consider things that you like/dislike)*? _______________________
2. Could you suggest how this could possibly be improved? _______________________
3. What do you think of these? *– Open discussion based one likes, dislikes, reasons why etc.*
4. Has this changed your initial thoughts of using visualisations for feedback? How? Why?
5. How do you think the general population will find the use of visualisations? Any thoughts around the level of detail and their ability to interpret what is presented?
6. How do you think the general population would respond to movement quality feedback?
7. How do you think that users will use the information they are provided?
8. Do you foresee any concerns that people may have about the information that could be provided?
9. Where do you think people would be willing to wear a monitor to ‘capture’ their movement? (prompts: ankle, wrist, thigh, waist)
10. Do you see any potential barriers or facilitators to using wearables to assess movement quality?
11. What are your thoughts on introducing this in products?

Any other thoughts or comments?
