# Supplementary material for: Assessing and Enhancing Movement Quality Using Wearables and Consumer Technologies: Thematic Analysis of Expert Perspectives
Source: JMIR Form Res. 2024 Sep 13;8:e56784. doi: 10.2196/56784 (PMC11437222; doi:10.2196/56784)
Supplement: Multimedia Appendix 2 [file formative_v8i1e56784_app2.pdf]

PARTICPANT INFORMATION SHEET – INDUSTRY INTERVIEWS
(Version 1.1, Date: 01/07/22)

Project Title:
Industry insights and public opinions on the use of wearable technology and the delivery of feedback when assessing movement quality

Contact Details:
Thomas Alexander Swain *Prof Melitta McNarry*

[redacted]

1. Invitation Paragraph
Thank you for taking the time to read this information sheet. We would like to invite you to take part in our research study. Before you decide if you would like to join in, it is really important that you understand what the study is about, why the study is being done and what it will involve for those participating. We are more than happy for you to talk to family, friends, or colleagues about it if you want to.

If something isn't clear or you have questions, you can email us and we can discuss it with you. The decision to take part is entirely up to you and you will not be disadvantaged for future studies should you decide not to participate.

2. What is the purpose of the study?
Enabling people to move better can have profound benefits. It can help people to perform daily tasks with greater ease, avoid injuries, and live a more fulfilling life. The purpose of this study is to gather ideas, interpretations and feedback from industry experts about we can help people to move better by providing feedback to increase understanding and awareness of movement quality. We are particularly keen to get views on feedback methods that can be used to provide an engaging and effective way to help people move better during exercise. Unfortunately, it can be difficult for many people to get feedback about their movement, as this usually requires the help of a professional, such as a coach or a performance analyst. However, with the use of affordable wearable technology, we hope to overcome this limitation to better inform individuals of possible problems with their movements to help people get fitter and healthier and live more active, enjoyable lifestyles.

3. Why have I been chosen?
You have been chosen because you are an employee of [redacted] and are older than 18 years old.

4. What will happen if I take part?
We just want to hear your views about exercise, movement, and how you think we could use technology to help you to move better. To achieve this, we will be conducting one-to-one interviews around 60 minutes

long. Our findings from the interviews will help inform future developments, eventually leading to an intervention to improve how people move during exercise. The interviews will be conducted in-person at [redacted], or using Teams or Zoom if in-person attendance is not possible. Consent will be conducted using an electronic or physical consent form, and will also be provided verbally prior to the commencement of the interview.

5. What are the possible disadvantages of taking part?

It will require you to provide approximately 60 minutes of time out of your working day, up to a maximum of 90 minutes. The interviews should not address any sensitive information, but if you feel distressed during the interview we can provide you with information for support.

6. What are the possible benefits of taking part?

The findings of the interviews will be of great benefit to [redacted] moving forward, which are in line with the company's vision for the future. Furthermore, you will be giving valuable views on potential feedback methods that could dramatically change the way we assess movement and educate people to move better.

7. Will my taking part in the study be kept confidential?

Data Protection and Confidentiality

Your data will be processed in accordance with the Data Protection Act 2018 and the General Data Protection Regulation 2016 (GDPR). All information collected about you will be kept strictly confidential. Your data will only be viewed by the researcher/research team.

All electronic data will be stored on a researchers' password-protected computer and in a password-protected file. There will be no paper records. Your consent information will be kept separately from your responses to minimise risk in the event of a data breach.

Please note that the data we will collect for our study will be made anonymous. All transcripts and information will only be identifiable by participant number and not name. It will not be possible to identify and remove your data at a later date, should you decide to withdraw from the study. Therefore, if at the end of the interview you decide to have your data withdrawn, please let us know prior to leaving.

Data Protection Privacy Notice

The data controller for this project will be Swansea University. The University Data Protection Officer provides oversight of university activities involving the processing of personal data and can be contacted at the Vice Chancellors Office.

Your personal data will be processed for the purposes outlined in this information sheet. Standard ethical procedures will involve you providing your consent to participate in this study by completing the consent form that has been provided to you.

The legal basis that we will rely on to process your personal data is necessary for scientific research. This public interest justification is approved by the College of Engineering Research Ethics Committee, Swansea University.

How long will your information be held?

We will hold any personal data and special categories of data for five years.

What are your rights?

You have the right to access you personal information, to object to the processing of your personal information, to rectify, to erase, to restrict and to transfer your personal information. Please visit the University Data Protection webpages for further information in relation to your rights.

Any requests or objections should be made in writing to the University Data Protection Officer:-

University Compliance Officer (FOI/DP)
Vice-Chancellor's Office
Swansea University
Singleton Park
Swansea
SA2 8PP
Email: dataprotection@swansea.ac.uk

How to make a complaint
If you are unhappy with the way in which personal data has been processed you may in the first instance contact the University Data Protection Officer using the contact details above.

If you remain dissatisfied then you have the right to apply directly to the Information Commissioner for a decision. The Information Commissioner can be contacted at: -

Information Commissioner's Office,
Wycliffe House,
Water Lane,
Wilmslow,
Cheshire,
SK9 5AF
www.ico.org.uk

8. What if I have any questions?

Further information can be obtained from Alex Swain, the lead researcher, Topi Korhonen, or Prof Melitta McNarry using the contact details on the information sheet. This project has been approved by the College of Engineering Research Ethics Committee at Swansea University. If you have any questions regarding this, any complaint, or concerns about the ethics and governance of this research please contact the Chair of the College of Engineering Research Ethics Committee, Swansea University: coe-researchethics@swansea.ac.uk. The institutional contact for reporting cases of research conduct is Registrar & Chief Operating Officer Mr Andrew Rhodes. Email: researchmisconduct@swansea.ac.uk. Further details are available at the Swansea University webpages for Research Integrity. http://www.swansea.ac.uk/research/researchintegrity/."
