# Supplementary material for: Assessing and Enhancing Movement Quality Using Wearables and Consumer Technologies: Thematic Analysis of Expert Perspectives
Source: JMIR Form Res. 2024 Sep 13;8:e56784. doi: 10.2196/56784 (PMC11437222; doi:10.2196/56784)
Supplement: Multimedia Appendix 3 [file formative_v8i1e56784_app3.doc]

**Supplementary Information 3 - Transcripts**

[Participant 1 – User Experience (UX) 2](#__RefHeading___Toc167369085)

[Participant 2 – Customer Experience 10](#__RefHeading___Toc167369086)

[Participant 3 – Industrial Design 19](#__RefHeading___Toc167369087)

[Participant 4 – Research 31](#__RefHeading___Toc167369088)

[Participant 5 – Research 43](#__RefHeading___Toc167369089)

[Participant 6 – Research 56](#__RefHeading___Toc167369090)

[Participant 7 – Software Architecture 66](#__RefHeading___Toc167369091)

[Participant 8 – User Experience (UX) 77](#__RefHeading___Toc167369092)

[Participant 9 – Customer Experience 90](#__RefHeading___Toc167369093)

[Participant 10 – Product Management 100](#__RefHeading___Toc167369094)

[Participant 11 – Sales 111](#__RefHeading___Toc167369095)

[Participant 12 – Sales 124](#__RefHeading___Toc167369096)

# **Participant 1 – User Experience (UX)**

Tue, 8/16 1:30PM • 27:58

**Interviewer** 00:04

Okay, so just a general introduction for you. So, there's no right and wrong answers. It's just to get your perspectives, your thoughts. As discussed, prior to the recording, it's all confidential and anonymous. Just to explain, again, briefly what we're trying to do. Because you work in this industry, and are industry expert, we want to get your opinion on using technology, how we can use that to assess movement and how we can give feedback to potential customers and users of the products. Before we get started, would you be able, please, to just introduce yourself? Also, tell me a little bit about your role in the company, what it is you do day to day, but also, outside of the company, what you'd like to do in your spare time?

**Participant 1** 01:36

Yeah. I'm a UX designer here. And I've been working here for four and a half years. And I work with these devices [points at smartwatch on wrist]. So we also have work designers in the service side, but I work especially with these devices. So that's a bit special platform. And, what I do in free time... I like sports. I like to ride horses. And I like many kind of sports. Almost anything.

**Interviewer** 02:18

What about outside of sports? Anything? Other hobbies?

**Participant 1** 02:24

Yeah, I like painting, playing guitar.

**Interviewer** 02:30

Okay. So, as I mentioned, some of the questions might be answered in earlier parts. So are you physically active in your spare time? So, yes. Okay, so a lot of the project, we speak about movement quality. So how do you interpret the term 'movement quality'?

**Participant 1** 03:03

Yeah, I was thinking about that. Maybe it's, it's like doing one movement in a good or bad way. Like, I expect that is what you mean. But also, it could be like, overall, like, movement that you use your body in many ways to improve strength?

**Interviewer** 03:28

Okay. So what sort of things do you think help people move well? First of all, what do you think helps people to move well during everyday life?

**Participant 1** 03:49

Yeah, maybe at least one thing that comes to mind is that if you have some problem, for example, in your body [injury], you must think about how you move like, for example, I have sometimes pain in my lower back. So I need to think about it and try to do strength training to improve the muscles.

**Interviewer** 04:15

And then to move on from that a little bit. What about when you exercise specifically? So not just in daily life, but actually when you're doing exercise? What sort of things help people to move well?

**Participant 1** 04:35

At least trainers.

**Interviewer** 04:41

Okay. So the next one is a similar question. It's what do you think influences how people move? So like, external factors that might help people to move better? I think, actually, that one, you said about the injury. So I think that kind of comes together. So we'll move to the next one [next question]. So what benefits do you think people could have by moving better?

**Participant 1** 05:15

At least avoiding the injuries. Like before they come [before they get injured]. Also, living with injuries, if they ask. Also, if you like to do sports, it's good to do it better, so you can do it.

**Interviewer** 05:35

So when you do exercise, what sort of information do you think is good to have to get you to move better?

**Participant 1** 05:49

I think it depends on what you do. But at least for me, I do riding [horses]. So it's really important to get, like, advice from a trainer to do things right. There are so many things to think about.

**Interviewer** 06:13

We're exploring ways to help people understand how they move, and how they can improve the way they move during exercise. Currently, there are no affordable, accessible and effective ways in which this can be achieved. So when I say about affordable and accessibility, I'm thinking about, like, maybe a physiotherapist. You have to go somewhere you have to pay, it's not cheap. Maybe have you seen the optical capture devices with the markers, which are big systems in a lab. But with the use of wearable technology, we're hoping to change this and make it more accessible for people. And we're also exploring ways that makes the data easy for people to interpret, so they can implement it safely and effectively. So on that note, can you give me some examples of feedback methods that you're aware of that help inform people of their activity? So first of all, ways in which people get information about how much they move. So not just movement quality, but how much they move?

**Participant 1** 07:36

Our devices, collect that data and, like, distances, durations, those kind of things.

**Interviewer** 07:44

Yeah. And then about how well they move.

**Participant 1** 07:48

That is a bit more difficult. But yeah, we have some metrics, like heart rate, you can follow what kind of heart rate you use.

**Interviewer** 08:05

I think also on that one, we've kind of covered that with the trainer [personal trainer or a coach] as well. So like a trainer actually gives you that feedback. So we've been really drawn to the use of, like, visual aids. So obviously, with your UX [User Experience - job role] experience, you know, all the graphics. So that's what we're talking about with the visuals. So I've kind of answered that question for you [the next question]. But in terms of using visual aids, what do you think people would think of those? Do you think they'd be well received? Do you think that they wouldn't like them?

**Participant 1** 08:57

Do you mean like, showing visually how used to do some-?

**Interviewer** 09:02

Yeah. So if I use the example of the [company] devices, if you look, obviously, they have the, the rings to see how active you've been [referring to a specific company device feature]. Or you can go on to the app, and it talks about, like, the sleep; you have the graph. So those are the visual things, but we're thinking that type of thing, but for movement quality. So do you think that would be something that people would like?

**Participant 1** 09:33

Yeah, usually people like visual feedback. It's easy to learn. When learn to read it, it's easier.

**Interviewer** 09:44

Yeah. So can you think, again, we've covered this a little bit, but can you think of any examples of visualisations that people might use to help them do an exercise

**Participant 1** 10:05

For example, I, [unintelligible - 10:07] came to my mind. We have this jump test in our devices, we have this visualisation of the stick figure doing the movement in the right way.

**Interviewer** 10:18

Yeah, I know the ones you're talking about. For [specific company feature]?

**Participant 1** 10:24

Actually, that's another one. But for jump test.

**Interviewer** 10:29

Okay. And then, so, when we're giving the visual feedback, do you think it would be best provided in isolation? So that would be you just give them a visual aid? Or do you think it would be better to include other types of feedback with it? So for example, you might have something on the screen, but then you might also have audible information? Or maybe the device will vibrate or something like that? Do you think it would be better just to have the visual? Or do you think there's a benefit to having other types of feedback at the same time?

**Participant 1** 11:19

Yeah, of course, it depends on the situation, but I would say that, it is better to have something more to at least to understand the visual feedback.

**Interviewer** 11:32

Okay, so obviously, within [company], you have the wearable devices, but also they interact with other devices. So for example, there is the watch, but then there's like the [company] apps, but then, also, there's an online facility. So if we're giving feedback, what devices do you think are best for giving the feedback?

**Participant 1** 12:07

I guess it depends on the situation. So, the wearable device is so close, you can use it when you do the training or anything. It's near you. But then, if you want like more deeper feedback, it's better to do in some bigger platform.

**Interviewer** 12:27

Yeah. Okay, so how much detail would you expect the feedback to provide? So remembering the customer base, do you think they would like a lot of detail? Or do you think they'd like something more simple? How much detail do you think that they would want to receive?

**Participant 1** 12:55

It depends, but they might want a lot of data, but it must be so that it's not everything comes to your face at once. You'll be able to find more information.

**Interviewer** 13:10

Okay. So, I'm going to show you a couple of examples of visualisations and I'll explain each one. And then we'll just sort of have a discussion about each one. What you like what you don't like, why. So, the first one is, is this one [Figure 1]. So, without telling you what it is? Do you think you can understand what it shows?

**Participant 1** 13:53

[Participant 1 inspects Figure 1] I guess this is some movement. These other things are, like, the dimensions.

**Interviewer** 14:12

So to explain what it is, basically, the coloured line is the wrist directory of someone throwing. So someone wore a device on their wrist, they did a throw with a ball and that shows the path that it took, but also the speed at each point in the throw. So, you can imagine as it [the arm - action demonstrated] goes back, and then it slows down and then it accelerates again. The green line is the gold standard. So that is what we're trying to achieve. And the pink area gives us a level of tolerance. So if it went there [points within error limits] it would still be okay. But if it went there [points outside of the error limits], it wouldn't be okay. And then just some extra things just to show you the direction throws going [points at direction arrow]. And just some information to explain that. So, first of all, what do you like about it? What don't you like? And why?

**Participant 1** 15:25

I like at least the speed colour is really simple to understand. Maybe this, like, how it is, it is a bit difficult to understand [where] the movement goes. I don't know if it's possible to see it from other angles? Yeah, but [unintelligible - 15:48] understandable. Yeah.

**Interviewer** 15:49

Okay. Then the next one [shows Figure 2]. Do you think you'd be able to understand that one?

**Participant 1** 15:57

Yeah, I've been using Apple.

**Interviewer** 16:01

Yeah. So what do you like about it? What don't you like about it?

**Participant 1** 16:08

I actually like that a lot. I think it's, it's simple. It collects everything automatically, so you don't need to collect it by yourself.

**Interviewer** 16:23

And then the last one is this one [shows Figure 3]. So again, can you understand what it shows? And then what do you like? What do you not like?

**Participant 1** 16:44

Is he wearing some sensors?

**Interviewer** 16:45

Yeah, so he's wearing sensors. You can see one on his thigh? There's one on his chest. And his back as well.

**Participant 1** 16:53

Yeah. So this shows, like..... I'm not sure if it is the same position that he has, or is this something he should have [points at human model in Figure 3]? Maybe it is the same as he is, but then these are telling how he should change the position?

**Interviewer** 17:17

Yeah. So the idea is, is that as he bends over, this line, marks it so he wants to be within this green zone. And then it'll tell him if he goes outside, and then that shows each repetition and where his angle was within each repetition. So that's like, that point [points to Figure to show where markers align with the different parts of the figure]. That is at point [points to Figure to show where markers align with the different parts of the figure]. So you can see each rep he's trying to be in that position. So again, same questions, what do you like? What do you not like?

**Participant 1** 17:59

I like this upper part [points to top part of Figure 3 which features the human model]. I think this is clear. And nicely visualised. But this lower part [points to lower part of figure, which shows a graph], I'm not sure if I understand it, at least now.

**Interviewer** 18:15

Okay, yeah. So I think you quite like this because of the simplicity [points at top part of Figure 3]. Whereas this [points at bottom part of Figure 3] is a little bit more complicated to understand. Do you like the use of text?

**Participant 1** 18:36

Yeah, yeah, it helps to understand it. We have these angles here. So is that the angle of the back or?

**Interviewer** 18:52

Yes, so I think it would be this [points to human model on Figure 3 to indicate relevant part]. So, if he was more upright [points to first limit on Figure 3], but as you go forward that would move [points at black marker on top part of Figure 3] but it sets the limit.

**Participant 1** 19:04

Yeah, that makes it more understandable when you do it like that.

**Interviewer** 19:09

So having seen those, did it change any of your original thoughts about using visualisations for feedback?

**Participant 1** 19:30

Yeah, maybe the last one [Figure 3] gave some inspiration. Yes. Just a bit different kind of...

**Interviewer** 19:38

Okay. So, how do you think the general population, so that would be the main customer base, would find the use of visualisations?

**Participant 1** 20:02

Maybe the first one was a bit technical, it would require some learning. But the others were quite easy to understand. I think they would be useful.

**Interviewer** 20:15

And then having seen those, have you got any thoughts about the level of detail? And how easy they'd find it to interpret? So obviously, you've said, the first one was quite technical. So would that be more towards the simple... You think the simple ones are better?

**Participant 1** 20:40

Yeah. Yeah, maybe. Yeah. For not everybody. But of course, there are some people who want like, really technical and want to see the real data and everything.

**Interviewer** 20:56

Okay, so how do you think the general population would respond to movement quality feedback? So I know there's similar questions. But if we were to give them feedback during a movement, do you think it would be something that they would like? Or do you think there'd be some resistance against it?

**Participant 1** 21:24

Yeah, I think for example, for those strength training type of movements, it would be really useful to have those guides that really, like, measure you and so the user, they assess and solve the movement, but also measuring if you do it right or not.

**Interviewer** 21:45

And how do you think the users would use the information that we would provide?

**Participant 1** 21:54

Yeah, I think I think they could use it for their trainings. To correct from wrong positions.

**Interviewer** 22:01

Yeah. Do you foresee any concerns that people may have about the information they get? So if we were to give them feedback about their movement? Do you have any concerns about how people might react to that information?

**Participant 1** 22:22

One thing that came to my mind, like, how we measure it. Would it be the trust to the measurement? If we have many sensors, it would be quite good. But if we have only one, give us in the wrist, it might be a bit guessing.

**Interviewer** 22:43

Okay. That's an interesting one, which takes us on to the next question quite nicely. So where do you think people would be willing to wear a monitor to capture their movement?

**Participant 1** 22:57

Yeah, that's a difficult one, if we want to measure, like, it depends what we measure. For example, in strength training, they could have it, really, on hand or anywhere, but depending on the movement, but if you're trying to measure like bigger movements, full body things, it could be a bit difficult.

**Interviewer** 23:19

What about where people are comfortable wearing one? So if you just forget about what movement they're doing, I think the watch is an obvious one [wrist worn sensors]. But what about other areas? Do you think people will be comfortable wearing it on the ankle? Chest? Their waist?

**Participant 1** 23:44

At least there are devices like heart rate sensors on the chest and, and optical heart rate [sensors], the smaller ones, they can be used on arm [points to upper arm] or even on feet. But then, then if it's, like, they need to buy it just for that, maybe that might be a problem. But, if they can use it for other purposes, too....

**Interviewer** 24:08

Yeah. So you've already given some examples for the next question, but do you see any potential barriers or facilitators to using wearables to assess movement quality? So basically, do you see any problems or ways in which we can use the wearables to assess movement?

**Participant 1** 24:36

Yeah, maybe that that's the one that I mentioned. Like, if you need to buy something extra to measure, it might be as easy to go to trainer [personal trainer or coach]. So even do the effort of purchasing something.

**Interviewer** 24:57

Yeah. Okay, so it's actually nearly the last question. What are your thoughts on introducing something like this in [company] products?

**Participant 1** 25:19

Yeah, I think it's, it's a good idea, definitely a bit difficult to, maybe, produce..... depends on what we are doing with it. Like, is it for some sports only? What kind of things? But I think it would be something people would like. Would be useful.

**Interviewer** 25:42

And then the last question is, whether you have any other thoughts or comments at all, related to the project? Any ideas with the wearable technology? Any ideas with the feedback? If you had anything else you wanted to say?

**Participant 1** 26:00

I was thinking about, like, some kind of video algorithm, I don't know, would it work? So that someone could just take video of their, like, swimming or some... whatever training. Could be somehow used for visual data? I don't know if it's possible. And then of course there are these old ideas like wearable clothes, smart clothing, or whatever. They will always require some something to buy, but-. Also, I was thinking about, like, maybe it's not exactly what you were thinking about, but, like, offering some guidance to, like, improve.... how would I say it? Like, like exercises that would help you do the training like they are not the training but they improve the muscles. Understanding of your body to help you to learn to find your muscles and how to use them. That's kinda, [unitelligible 27:31] digital or technical things.

**Interviewer** 27:35

Okay, great. Any other comments, or?

**Participant 1** 27:43

Yeah, this is it, really interesting, yeah.

# **Participant 2 – Customer Experience**

Wed, 8/17 1:47PM • 39:06

**SPEAKERS**

Participant 2, Interviewer

**Interviewer** 00:07

Okay, so just a general introduction to start with to let you know that there are no right and wrong answers. It's just to get your perspectives and your thoughts. As discussed before the recording started, it's all confidential and anonymous. Just to give a bit of an introduction to what we're doing. So we're looking at ways in which we can use wearable technology to measure and assess movement quality. So the distinction between quality and quantity, so we can already do steps and heart rate, calories, but it's more about how well we move. So, before we get started with the questions, could you just give me a bit of an introduction about yourself, so your role in the company, but also then what you'd like to do outside of work in your spare time?

**Participant 2** 01:11

Oh, I've been here for 11 and a half years. So most of it, I've been kind of in the frontline. So talking with customers. I do communications, customer experience, design and so on. Here I've also worked for for social media, marketing. So a lot of different things. Outside work, volleyball, that's been my favourite since a kid. Beach volleyball. Gym. Walking. Okay, great.

**Interviewer** 02:06

So generally quite active?

**Participant 2** 02:08

Yeah.

**Interviewer** 02:09

Okay. So as I've said already in some of the questions, it's a template. So some of the questions if we've answered them in a previous part, I'll go beyond that. So like, the first question is, are you physically active? So we've already established, yes, you are. So I spoke in the introduction about moving well, the term 'movement quality', how do you interpret 'movement quality'?

**Participant 2** 02:59

I think usually, people have some goal, kind of, whether it is physical or mental or something else. And I feel that if we're talking about quality of movement, then it kind of felt it's good for the goal.

**Interviewer** 03:32

Okay. So what sort of things do you think help people to move well, so, first of all, during everyday life, so you're coming to work you're doing your shopping. What sort of things, if anything, are you aware of that could help you move well during everyday life?

**Participant 2** 03:58

Well here, weather effects a lot. Like, with yesterday evening [referring to heavy rainfall and thunderstorms] I wouldn't go by a bicycle to work in that weather. Also, during winter time, I think it's so dark here. So, I think that affects as well. I think people move more in the evenings and then if it's darker, it's also easier to kind of stay home and then and if it's cold, then enjoy outside.

**Interviewer** 05:08

So that's during everyday life. What do you think helps people move well, during exercise? So think about, like, the technique that you use, what sort of things are you aware of that might help somebody do exercise with better movement?

**Participant 2** 05:30

Understanding of what is done, like, for instance, I got back to gym after, I don't know, a decade or two. So it took a while to kind of find my way. And then on the other hand, to understand that I need to really use heavy, kind of heavy weight the to get results?

**Interviewer** 06:16

So just while we're on that topic, you said about you haven't been to the gym for a long time, and then you went back. What sort of things when you were done doing exercises? Was there anything that helped you to learn or remember how to do those exercises properly? Or did you just, kind of, figure it out?

**Participant 2** 06:48

I also had a personal trainer. And I've been using the internet to search for information. And I think there's also, I understood later on how important for us, for instance, kind of the flexibility is for being able to do a good gym training.

**Interviewer** 07:18

Yeah. So number three and four [questions 3 and 4 on the interview schedule] are somewhat related. So we've kind of covered four [question 4]. So, when you move well during exercise, what do you think are the benefits of of doing that? So doing the movement well, instead of badly. What do you think are the benefits of doing it well?

**Participant 2** 07:46

I have a better feeling after the exercise, because the exercise can be bad as well and it affects it affects the day, it kind of feels that this gym training didn't go well.

**Interviewer** 08:09

Is that because you didn't feel like you had a good workout? Or is that because if you did the technique badly something is hurting?

**Participant 2** 08:24

Perhaps I didn't have enough kind of energy to pay attention to everything I do. And perhaps I just didn't push myself enough.

**Interviewer** 08:41

So when you are trying to improve how well you move during exercise, what information do you think is important to have that will help you move well? So, if we look at like the personal trainer, or when you're looking on the Internet, what sort of information are you looking for that will help you move better? So like you could be doing a squat. What information do you think they could give you that would help you do that exercise better?

**Participant 2** 09:27

Kind of personal, because I have a lot of injuries behind from volleyball. Like with my knees, so the internet won't tell me they can't tell me what to do with my knees. So that's where the someone who understands is better.

**Interviewer** 09:57

Yeah. So it's kind of how you can do the exercise safely, and work around your injuries at the same time, and then having that person to tell you.

**Participant 2** 10:13

And when I feel safe with it, then I have the motivation to lift up the weights and....

**Interviewer** 10:29

So we're exploring ways to help people understand how they move, and how they can improve the way that they move. So, currently, there are no affordable, accessible and effective ways in which this can be achieved. So for example, you have a personal trainer, but you have to pay for each session, or maybe you have an injury, and you need a physiotherapist. Also, there's systems, I'm not sure if you're aware of them, but they use like cameras. But you need to be in a big lab to do the assessments, so, and they're expensive, and they're kind of reserved for elite athletes. But what we're hoping is that with wearable technology, we can make it more accessible for them, and also more affordable. So we're looking at ways of using wearable technology to use the data we collect, give it to people, make it easy for them to interpret and that they can then use that information to implement it safely and effectively. So that's the idea. So are you able to give me some examples of feedback methods that could help inform people of their activity. So first of all, like, measuring how much activity they do. So ways in which people can get information about how much activity they've done. [long pause] So just to give an example, then, obviously, the company, we use the devices to track steps and things like that. So that's an example of how we get feedback on how much exercise we've done. So obviously, there's the devices. But are there any... can you think of any other examples?

**Participant 2** 12:54

I think heart rate readings is one, how, like, heart rate zones. How much you've been in certain zones and how it affects and how it should feel and that kind of thing.

**Interviewer** 13:12

And then moving on from how much they move. Again, it's coming back to how well they move. So can you think of any examples, perhaps other than the personal trainer, that could maybe tell you how well you move?

**Participant 2** 13:33

I think it's also with the heart rate zones, in a way. Understanding, well, they need to be correct, first of all, but understanding how it how it affects and what is your goal.

**Interviewer** 13:50

So, we've been really drawn to the use of visual aids. So, like, things people see when they're getting feedback. So first of all, what do you think people would think of that? Do you think people would like to see something visual? So if we would measure movement? Something visual? Would you like something like that?

**Participant 2** 14:25

Yes. I think goals are visual. How well are you? You have a certain goal for, I don't know, whatever period. Well, some people used to step goals, 10,000, and how much you've achieved from it, and then daily activity with our watches, it's another one. I think it helps and it motivates.

**Interviewer** 15:01

So can you think of any examples of these visual aids? That might help people do an exercise? It doesn't have to be specifically for movement quality, it could be either quantity or quality.

**Participant 2** 15:27

Well, their daily activity was one. But if, training plans? Then how you performed it?

**Interviewer** 15:37

That's an interesting one, actually. So when we provide a visual aid for somebody, do you think it would be best to give them that visualisation? So that would be like, some sort of image or like a graphic? Do you think it'd be best to give them that on its own, just in isolation? Or do you think it would be best to give that visualisation, but with other information, like, for example, an audible sound? Or maybe like vibration? To give you that information?

**Participant 2** 16:27

I think that that's kind of personal preference.

**Interviewer** 16:33

Okay.

**Participant 2** 16:34

But I think, I think for most people, seeing it is enough. And on the other hand, we get a lot of, kind of, notifications daily from phones and everything. So I think audible or vibration or something, it could feel more, like, a distraction, or, you know, like, with a phone, you get all messages and emails and stuff. So, so it feels a bit different.

**Interviewer** 17:11

Okay. So what sort of devices do you think will be best to get the feedback? So for example, you do an exercise, you want to find out how well you've done the exercise, a device is going to give you information. Do you think it would be best on a watch or a mobile phone app or a computer?

**Participant 2** 17:43

I think it depends also on the person, but mainly, mainly watch or the app. Like, we have, we [the company] have both, like, exercise app as well. So it depends on which one you use. I believe that when people go for an exercise, and then whichever they choose to have, they would like to see, kind of, the success, if you can say so, there, where they have used for recording.

**Interviewer** 18:31

So when you get information about how well you've moved, how much detail would you expect to have? Or how or even how much detail would you want to have?

**Participant 2** 18:53

Not much. I think for me, heart rate readings are the ones that I follow.

**Interviewer** 19:04

Okay. So, if I just explore that a little bit more. Is it that detail is just too much to think about when you're exercising? Is it that you want something simpler, or is it because you get it quicker? Or what's most important to you?

**Participant 2** 19:30

I've learned to know myself, based on what happens during exercise and how my heart rate acts. So that's kind of enough for me.

**Interviewer** 19:42

Okay, so it's not having too much information, so you can kind of get on with it? [Participant 2 nods in agreement] So I'm going to show you a couple of examples of visualisations that exist. Most visualisations that exist, generally are around, like, how much people move. The ones I'm going to show you are a bit of both, but it's not really about.... it's trying to understand, if you can understand what they show. So first of all, I'm going to show you each one, one by one. And then we're just going to discuss whether you like it, what you don't like about it. Okay, so, this is the first one. So without telling you too much about it, do you think you could understand what it shows? Just take a minute. Just see if you can understand what it's showing.

**Participant 2** 21:00

No, and honestly, for me, it's..... I just can't get a hold of it in any way. And I lost interest, like, in a second.

**Interviewer** 21:15

Okay. So if I explain it, just so you can understand what it's showing. So this is somebody throwing a ball. So this is where wear watches worn on the wrist [points to Figure 1 line of trajectory] and as they throw, this is the pattern, their wrist followed. So the green line is the best type of throw. And this pink area [points to error band in Figure 1], is kind, of like a tolerance for how much error. So if it's inside the pink, it's okay, if it goes outside, the pink gets not okay. And then the colour [of the throw trajectory] shows the speed. So it starts off slow, and then as it moves, it gets faster. And then it slows down, and then it goes fast again. So that's what it shows. So on that note, is there anything you like about it?

**Participant 2** 22:22

No.

**Interviewer** 22:22

What is it that you don't like about colours?

**Participant 2** 22:32

Colours.

**Interviewer** 22:32

Too much colours? Or the specific colours that were chosen?

**Participant 2** 22:38

I don't know. It's just really weird.

**Interviewer** 22:47

Do you find it's, like, too complex? Do you think there's too much detail?

**Participant 2** 22:52

Yeah.

**Interviewer** 22:52

Okay. Moving on to the next one, which is this one. This is obviously an Apple Watch. Again, do you think you can understand what it shows?

**Participant 2** 23:07

Without having any other information, no.

**Interviewer** 23:14

Okay, so the rings show how much activity you've done. This one shows how much total activity you've done [points to outer red ring on Figure 2]. And then these ones show you like, how intense it's been [points to green ring on Figure 2]. But again, is there anything you like about that?

**Participant 2** 24:04

I don't, I don't understand it. Like, if I think about my day, yeah, I, I don't understand it because I kind of feel, like, I'm not sure if there's some goal behind it?

**Interviewer** 24:21

Yeah, so the idea is, is that as you come around, that's your target [points Figure 2 and points at the ring moving around the watch face to make a complete circle]. So, like, this would be your total activity. So once that ring is completed, you meet your activity goals for the day. That's the idea.

**Participant 2** 24:36

Well, it's making some sense now.

**Interviewer** 24:41

So is there anything you dislike about it?

**Participant 2** 24:48

I don't care about calories.

**Interviewer** 24:52

Okay, and then the last one is this [Figure 3]. First of all, I'll let you look at it. Just for some context. He's wearing wearable sensors. So he's got like one there, and there's one on his chest.... And he's doing a squatting movement. So, with that in mind, do you think you could understand what that is showing?

**Participant 2** 25:25

Yeah

**Interviewer** 25:28

Could you explain please?

**Participant 2** 25:37

I think we're, kind of, back to the technique. And that stuff that, like I said, it's important that you do what's right, so you get the results you want to have. So that would help in understanding, because we easily make the movement easier. So I think that would help in preventing that.

**Interviewer** 26:11

So, obviously, in this one, he's doing the squat. So this is referring to his torso angle. So, he starts off standing up straight. And then as he comes down, he bends forward. But all this is doing is this black marker [point to black marker on ring around human model on Figure 3] is supposed to stay in the green zone [points to green zone on ring on Figure 3]. So as he goes down, that will move. And if he goes outside, he gets that information. And then that tells him the last reps, what his angle was [points to graph at bottom of Figure 3]. So first of all, what do you like about that? And then also, what do you dislike?

**Participant 2** 27:04

Well there are two sensors.

**Interviewer** 27:21

So you'd like that there's less sensors or more?

**Participant 2** 27:32

I perhaps wouldn't want to wear that. Personally, because I kind of want everything to be easy. And then there's also another thing that this is part of what I said earlier, about technique and everything, I feel that I should be able to, kind of, trust the feeling that I have in my body. And if this.... if I feel that I'm doing it right and this says otherwise, and it's wrong, I probably wouldn't have that again.

**Interviewer** 28:27

Okay, that's really interesting. So yeah, thank you. On to the next question. So thinking about what I've shown you here, has that changed your thoughts on how we could use devices to give people feedback for how well they've moved?

**Participant 2** 29:15

Perhaps with the sensors, I think they could be, kind of.... when people start to exercise, all the, the exercise goes to a next level. In a way, they could be of help at that point, to ensure people are moving better, so that it would kind of be the personal trainer, in a way, in the beginning. And then you wouldn't need to use them anymore, because you're doing it better.

**Interviewer** 29:57

So if we going to use a visualisation like that. You're thinking maybe it's best to have it for teaching someone the movement at the start? And then they kind of move away from it as they get better?

**Participant 2** 30:13

Yes, because they because they also need to trust what they do with their bodies.

**Interviewer** 30:20

Yeah. Great. So how do you think that the general public and the population and the customer base would find the use of visualisations? And do you think they should be generally quite simple? Or do you think they like detail?

**Participant 2** 30:49

I think it depends, kind of, what kind of athlete or sports person you are, because if you are starting it from scratch, I think it should be very simple and easy to go forward. But then, if you're like me, that I would like to go to the next level. And, I just need the right instructions for plans for it, then I could pay more attention to more details.

**Interviewer** 31:38

So how do you think they would respond to the feedback? Do you think.... I know, we've spoken a little bit about this already, but if if we had a feature in the device that would give them feedback, do you think it's something they would use?

**Participant 2** 32:04

I believe so. Yeah.

**Interviewer** 32:08

And then how would you think they would actually use the information we provide?

**Participant 2** 32:19

I think it would motivate to... because it motivates you to, kind of, continue with the exercise or exercising. Because you know, you're doing it right.

**Interviewer** 32:40

Yeah. Now, do you see any concerns that people may have with a feature like that? And if I actually come back to the picture I showed you [Figure 3], you spoke about it. If it feels right, but the device is telling you it's not right. You might not like that. Do you think... so that's an example. Do you think there would maybe be any other concerns people would have?

**Participant 2** 33:14

Trusting the technology. We are so different, physically. So is it the standard for everyone? Does it take into account my injuries or my limitations?

**Interviewer** 33:41

Okay. So, then, talking about wearing the sensors. So I think you've already said that you wouldn't necessarily want to wear too many sensors. Where would you think.... Where do you think people will be willing to wear the sensors? So of course, the watch is kind of an obvious one. During exercise, do you think people will be happy to wear it on the wrist? Or their ankle or foot? Maybe the chest or the waist?

**Participant 2** 34:23

That's really hard. Because like, with gym training, there can be so many different exercises that pretty much, well, watch it sometimes in the way. So, I don't know. Perhaps, upper arm [points to humerus] or lower arm [points to forearm] can be one. I think ankle is a bit difficult but perhaps like, upper leg in a way. So not the ankle but 10 centimetres higher or something, so, perhaps.

**Interviewer** 35:09

Okay. So would that be, you mean like, so if that's my ankle [points at ankle], it would be like there [points at middle of shank] not there [points at thigh].

**Participant 2** 35:17

Yeah.

**Interviewer** 35:17

Okay. Do you see any potential barriers? Or ways in which we could use the wearables to assess the movement quality? Okay, that's poorly worded [laughter]. Do you think there's any..... what do you think of the limitations of using wearable technology? And then what do you think are the strengths of using it for movement quality?

**Participant 2** 35:53

A strength, guidance, first of all. Understanding your body. Limitations, to get people, kind of, understand the strengths, the benefits, because probably many think that "I don't need this stuff".

**Interviewer** 36:30

Okay. And then, last, proper question is, what are your thoughts on introducing something like this in [company] products?

**Participant 2** 36:43

Definitely worth it.

**Interviewer** 36:44

And then, finally, do you have any other thoughts or comments or ideas that you might want to say about now that I haven't spoken about already?

**Participant 2** 37:08

I think in today's world, people are busy. There's so much information everywhere. And although you would like to get more information on the exercising, there are so many sources that you kind of need to first understand which one is valid? Which one is is ...**...? And already that can make the motivation drop very much. And another thing I feel, in the busy world, when you, kind of, have the time, you shouldn't need to make a choice, in a way. Because, for instance, if you ever every morning if you choose your clothes previously, you spent a lot of time you've done that choice already before. Instead of like, "what should I wear? Does this feel good?" I think it's the the same with exercising it's much easier if you..... if, kind of, the choices have been made for you.

**Interviewer** 38:51

Yeah. I understand. Yeah, so that's the end of the interview.

# **Participant 3 – Industrial Design**

Thu, 8/18 1:19PM • 52:33

**SPEAKERS**

Participant 3, Interviewer

**Interviewer** 00:13

Okay, so thank you for helping with this interview. As we discussed off the recording, it's quite informal. There's no right and wrong answers. It's just to get your perspectives and your thoughts. Everything is confidential, anonymous. And then to just give you a brief introduction of what we're doing. So with exercise and activity, wearable technology has been around for quite a while now. It's still, it generally focuses on movement quantity. So it's like, how much activity you do. So how many steps, heart rate calories, and so on. What we're kind of looking towards is more around how well people move. So when they do an exercise, how their technique looks, but also how we give users the information about how they've done the technique. So before we get started with this, the questions, there will be quite a bit of reading from a sheet, but most of it [unintelligible] and some questions you won't answer because you may have answered them previously in a different question. So it's quite fluid. Before we get started with the questions, would it be possible please just to introduce yourself, tell me a little bit about your role at the company. And then also give a bit of insight into what you do in your spare time outside of the company as well.

**Participant 3** 02:04

Okay, so I'm (...) and I'm an industrial designer. Working in our team, there's five of us. And I've been working for [company] almost 22 years. So, as a design team, what we focus on is the outlook of the product, whether it's a wristband, or the sensors or-. And me specifically, at the moment, for a couple last years, few years, last years, I've been working more on the colours and materials. So there's some. Every project is divided, somebody's taking care of-, somebody who's responsible of the 3D [3-dimensional design]. And then then I've been working pretty well, all of the other three guys like with the colours and materials. So mainly that. And obviously, we have a lot of internal customers, so like, project managers, and marketing and having to conduct for all the different teams. So that's, in a nutshell, what we do in the design team. And like I said, I've been working here quite long. Our team is pretty old in general, like the member of our team of four, he's been already working for six years, and all the others over 20 years. So we kind of know each other pretty well. And the company as well. And what do I do outside? I have two kids, and a husband and a dog. So I walk regularly every day. He [the dog] has to be taken for a walk. So that's probably my main like exercise. But I also like actively taking part in yoga. I like swimming. I do different kinds-. I go kayaking in the summertime. I just went for a hike for the weekend. I ride bicycle. I sometimes take my inline skates and I go skating. And wintertime skiing, cross country skiing, downhill skiing, all kinds-. A little bit of everything. But nothing as regularly as just purely walking. So that's about my exercise habits.

**Interviewer** 04:56

So generally active in your spare time?

**Participant 3** 05:00

Yes.

**Interviewer** 05:02

I spoke about in the introduction, we're looking at ways in which people can move better, and move well. So if I was to say the term 'movement quality', what does that mean to you? How would you interpret the term 'movement quality'?

**Participant 3** 05:28

Well, to me, personally, when I'm thinking about exercising, to mean, the driver is definitely health being like in a good place, because I'm not competitive. I've never been into competitive sports. And, okay, when I was younger, I wanted to run half a marathon. And so that was the quality. It was "how do I develop on that?" to get my goal, but nowadays, it's more that I'm mentally and physically balanced. And how will I sleep, all that, just wellbeing is my measurement for the quality of movement. And I think I totally recognise that I need to exercise to feel well. So that's my own perspective.

**Interviewer** 06:21

Okay. So what sort of things, then, help people to move well? So, first of all, in daily life. So, for example, you walk with the dogs, you have children, you probably do the food shopping, you come to work. What sort of things do you think will help people to move well, during everyday life?

**Participant 3** 06:55

That's a good question, and not not the easy one either. I have, from my own experience, how I feel if I exercise, or I don't it's like knowing yourself first. Like, how do you react if you exercise or not? Recognising your own now like needs. But then I think you need this internal motivation. You need to recognise the benefits, but then also living a pretty hectic life, you also need to be motivated to organise your day so that there is some space for exercise. That's a challenge for me. I totally know that I need to exercise but I don't manage to get that time. Because I have-. The yoga is the only thing of my exercise that is scheduled. I go to a certain place and there's a person who's instructing, teaching us. But other than that, it's all free. And I need to I need to find the time myself. And the only must, or only non-excuse reason is my dog, because he has to be taken out. Can you repeat the question again?

**Interviewer** 08:34

It was what sort of things help people to move well during everyday life?

**Participant 3** 08:40

Oh, goodness. How do I know? I think it's the internal motivation and the feedback. To me feedback is really rewarding, like what I've I've always liked. I've grown into using these devices, and I find it motivating for me to see that-. I don't keep track how many kilometres I've done, not really, but to see the calendar that I have is, like, exercise marks on more than once a week. It makes me feel good. And it's motivating and it's kind of like motivating me to keep it up. And if I see that, "Okay, there's two weeks and I haven't done anything, I have to get into this". So I-. Rewarding. I mean, the feedback is rewarding to me. It's motivating. It's somehow-. But I also know that there's a lot of people who get frustrated about that, or they don't like that. The the feedback is the wrong kind, or they get upset about it or something, but I don't know. I somehow like a little bit of statistics, but I like to sometimes just go see like how many times I've done this sport and have I done anything else but walking? And it's kind of like, so for me, the internal motivation and feedback. And obviously the way I feel.

**Interviewer** 10:14

So the next part is a similar question where it says about doing exercise. So, we spoke about moving well during daily life. But then what about when you're actually doing exercise? What helps you to move well? And I know you've touched upon that a little bit. But, for example, when you're doing your inline skating or your yoga, or your swimming, is there anything that tells you about how well you're doing the exercise technically?

**Participant 3** 10:56

Not that much about the technique, but it's about that-. At least one, I'm just using this, our device, and this is an Ignite. It's not the most advanced with all the features. But what I follow is the intensity of the training. So I do follow that. Training zones and how hard I go and keep on track of that during the exercise if it's possible. Mostly this. And in the yoga I don't because it's so like, low [the intensity], but sometimes I just want the mark that I've done it.

**Interviewer** 11:34

So in that one [yoga], you have an instructor as well? So that kind of helps you move well?

**Participant 3** 11:40

Yeah, totally.

**Interviewer** 11:42

They give you feedback. Okay. It's not a device, but it's a person actually giving the feedback.

**Participant 3** 11:45

Yes, yes. But other than that I do, like, if I go skiing or jog or something, or I'm climbing the steps or something, I do take a look at my heart rate.

**Interviewer** 12:01

So what benefits do you think that people could have from improving how well they move when doing exercise? So, this would be performing an exercise with good technical ability. So, if they were to run better, or if they were to swim better, what do you think are the main benefits of doing that?

**Participant 3** 12:30

Well, if there is a way to somehow evaluate the technical part of the exercise, then it's not only doing better, but also maybe avoiding injuries, definitely. I guess those two [do the movement better and avoid injuries], doing better and doing on the right level if you have set the goals, not overdoing either. And so that you have a better chance to recover better if you have that. You have a programme or something.

**Interviewer** 13:12

So, again, we're talking about the feedback and we've already said that you value feedback when you're doing the exercise. And talking about intensity with the heart rate and, I touched upon calories and steps. When you're moving, and you're trying to evaluate yourself technically, what do you think would be useful information to have about how well you move? So if you're doing an exercise, what sort of information about your technique do you think would be good to have?

**Participant 3** 13:44

Depends on the sports I guess. Like, but how would you be able to measure? Like, if I'm just thinking about some exercises that I do on my own if I go to the gym and there's nobody to tell me that I have the wrong technique. Like, some feedback of your technique? Obviously that benefit if I'm doing something wrong, but how do you do that? I don't know. That is a difficult thing. Well, obviously like that's a simple thing, like when training for a long distance something, skiing or running or something, then the heart rate and the zones where are you practice are important. But that's related to the speed and power, how hard you are training, and that is obviously is easy to measure. Again, comes through the heart rate. The technique, I mean, yeah, probably would be probably useful. I just don't know how.

**Interviewer** 15:10

So that's just the first point we touch upon- [touch upon movement quality]. So, as I sort of mentioned, again, in the introduction, we're exploring ways to help people understand how they move, and how they can improve the way they move, during an exercise. So currently, there's no affordable, accessible and effective ways in which this can be achieved. So if you think about the opportunities that people have to move better, often they'll pay maybe a personal trainer, or they have like expensive lab based systems that's maybe reserved for elite sports, and they're not really accessible. You can't just go to a lab every time you do a training session. So with the use of wearable technology, what we're hoping to do is explore some of the ways we can use it, to measure movement, and assess movement, but also make the data you get from that movement easy for people to interpret, that they can implement it and improve how well they move. So, first of all, and again, we have touched on this already, but can you give some examples of feedback methods that you're aware of that inform people of their activity? First of all, about how much activity they do? So this comes back to some of the things we've already spoken about with intensity. Can you think of any examples, specific examples of how you might get feedback for how much you've done?

**Participant 3** 16:59

That's included in our products already. That's pretty easily accessible. Because we have the training diary, they [company devices] measure-, we already measured that, how many times per week, how long do you do and routine intensity. Those are the things that are already there and all the time accessible. But then, if I think about something that we don't offer, but some other companies do. I know that there was, I don't know if it's available anymore, but somebody tried to-. They had these sensors that you wear on top of your muscle. And that's for the gym kind of exercise that were there. They were measuring the balance. And the technique. But, I guess the muscle balance, that you're doing the same sort of exercise, both left and right side. That's one thing. And there's all kinds of sensors like Stryd and stuff like that. They measured the step length when you're running and stuff like that. We also had the Stryd earlier [reference to a former device feature].

**Interviewer** 18:20

Okay, yeah. Can you give some specific examples, perhaps, of the features within the devices that you maybe personally use? So, for example, it shows you your daily activity, and then maybe on the app, are there any specific features of the watch or the app that you use regularly?

**Participant 3** 18:46

Myself? On our device, I do take a look of the intensity levels of the training. I know that I should do a little bit more of higher intensity. Just walking the dog is good for the base and it's good for your health and circulation and blah blah. But like to be in a better fit-, like, few spikes here and there on the higher higher intensity zones [reference to brief moments of higher intensity exercise and higher heart rate during training] would be good for sure. I would need to do more gym, like, muscle exercise, not only cardio. I do follow what I've done and the quality, or different [heart rate] zones and different elements. Stretching, mobility, or how do you say? Stretching, other than yoga that's totally lacking from my exercise at the moment. And I do follow also the time and now during the summer I took part in this kind of, like, a walking competition. Honestly, it's kind of like a team thing that was a national thing that [company] took part as a team. It was very voluntary. I just signed in because I wanted to know how much do I walk with the dog actually, per month. So I've been measuring the kilometres and I'm just keeping track and-. Because it feels that it's such an easy thing to do, the walking. So I just wanted to see how many kilometres. It's actually pretty motivating to see that. Just little walks with the dog, I walk over 100 kilometres per month, just like that. So it was fun to see. But other than that, I don't think I measure other things or follow.

**Interviewer** 21:15

The next part was talking about measuring how people move. But actually, I think we've already touched on that one, because you've said about the Stryd app. And you said about the muscle balance. So obviously, we've already spoken a lot about feedback. Something that we, as a research team have looked at is the use of visual aids. So, first of all, how do you interpret that? What do you think I mean, when I say visual aids?

**Participant 3** 21:49

Like as a guiding element? I'm pretty active in Instagram. So I come across all kinds of like apps that are offering exercise programmes, and they obviously use a lot of visual guidance. They have little animated videos, and whatever. So I mean, they're pretty clear I would think. I've never tried one [laughter]. I would think that they are easy to follow. And pretty clear to-. Just a clear way to see what to do.

**Interviewer** 22:30

So what do you think people would think of visual aids as a way of giving feedback? Like, if you think customers, do you think they like the use of visual?

**Participant 3** 22:45

I think they would appreciate that. I think that would be a positive thing.

**Interviewer** 22:54

Would you personally like something that like that?

**Participant 3** 22:56

I would. I don't know how familiar you are with our products?

**Interviewer** 23:02

Quite familiar.

**Participant 3** 23:02

Okay. So there's-. We have this FitSpark programme that we had this little funny, funny little-.

**Interviewer** 23:11

Stick person?

**Participant 3** 23:12

Stick person. And it's better than nothing. I liked it. And I've done quite a few of those exercises. I got got bored of it, because they never changed. I wanted them to update more different programmes, but also I know that with a different kind of display, you could do a better job. So that would be more clear, more visually interesting. Obviously, I'm a very visual person, because I like-. That's what I do for work. But, I like visual things. And I think quite a few other people would benefit of this kind of like help. It's easy to understand, I think, if it's done well.

**Interviewer** 24:00

So we'll hold that thought for a minute because there's something you said that I want to get into. But there's a later question, which comes to that. So, you've already given some examples of visualisations that people use because you've spoken about the FitSpark and you also spoke about, like, actual videos. If someone was to get feedback on how they move, and if you think of the products that you're aware of, and some of the stuff you spoke about already, do you think it would be best to give people that feedback as just the visual aid? Or do you think there would be benefit to having it with some other information? So you can have some visualisation telling you how well you've moved, but maybe you'll have some audio or some text or that sort of stuff?

**Participant 3** 25:00

Of course, if it is interactive. I'm just thinking about the situation that I'm doing an exercise through YouTube or something, and it would actually communicate with me, by just somehow the camera would be able to track my technique and giving me feedback, or is that-. Maybe my heart rate is online somehow, and they would say that now you're going too slow, just speed it up. Of course, that be lifted up to next level. Practice, I think.

**Interviewer** 25:47

This is where I'm coming back to some of the things you said earlier, what sort of devices do you think will be would be best to provide feedback? So, for example, [company] have the watches, they've also got the mobile apps. There's also a desktop facility. So those are just a couple of examples. So, you said earlier about having more detail, maybe better picture? Which devices do you think are best for giving this feedback? Or maybe even different devices are better for different things. But what are your thoughts on that?

**Participant 3** 26:26

Easiest, obviously, is phone, because everybody has one. And it's always there. But to think about that, the situation of exercise, if I'm if I'm doing some sort of exercise at home, and I'm like, communicator. That communication comes through a display, maybe a pad [tablet device], a little bit bigger pictures so that I can see well enough not to be close. So maybe a little bit bigger screen than a mobile phone. Just like an iPad or some sort of pad [tablet device], maybe. A laptop or computer? Well obviously TV. I mean, if you have a possibility to do this stuff in front of the TV, that'd be even better.

**Interviewer** 27:10

Yeah. Great. So if you're doing the exercise, and you're wanting to get information about how well you're doing the exercise, how much detail would you expect to have?

**Participant 3** 27:24

As like instructed exercise? Or guided exercise?

**Interviewer** 27:31

Either.

**Participant 3** 27:35

Really depends.

**Interviewer** 27:42

Yeah, so remember-.

**Participant 3** 27:43

Hard to say, because I've never done such-.

**Interviewer** 27:46

Remember, there's no right and wrong answers. So maybe some people like less detail, some people like more. Can you maybe-?

**Participant 3** 27:55

It depends. If it's about technique, doing something wrong, I want to be corrected because I don't want to get injured. That's one thing. But then if there's a lot of information, I'll get frustrated. It's gotta be supportive [laughter].

**Interviewer** 28:17

So what I'm going to do now is I'm going to show you a few examples of visualisations that exist. Mostly visualisations that exist in general, are around quantity of movements that we've spoken about how much activity people do, and that sort of thing. But then there's others that consider movement quality. So I've selected three which show a different range of things, and varying degrees of information. So, I'm going to show you each one and we'll go through each one and you can tell me what you like what you've don't like. So, not to try and catch you out, but I'm going to show you, this is the first one [Figure 1]. Without me telling you what it shows. You just take a moment. Do you think you can work out what it's trying to show? And then we'll get into maybe the things you like or don't like about it?

**Participant 3** 28:31

This one [points at Figure 1]?

**Interviewer** 29:24

Yeah.

**Participant 3** 29:26

Just by looking at the picture, can I read the texts?

**Interviewer** 29:30

Just what do you think it's trying to show?

**Participant 3** 29:34

Oh goodness. If I had to guess there's some sort of intensity in this. Like, it's growing and then it's kind of coming back. Some sort of-. But what does the loop mean? I don't understand.

**Interviewer** 30:04

Okay, so if I explain it to you then and then we can discuss maybe things that you like or don't. This is somebody throwing a ball. So what they've done is they've worn a watch on their throwing hand. And this is the pattern as recorded by the watch for the throw that they did [points at multicoloured line on Figure 1]. The green line is the best throw, it's the ideal throw, the [multi]coloured line is the one that they actually did. So the colours tell you the speed at which the wrist moves. So if you can imagine a throw, it starts off slow, it's blue. And then as it goes back, it speeds up. This point is where you get to the end of the throw, in which point it slows down again. And then you accelerate through and release the ball. And then the pink area is a tolerance. So it shows if it was inside the pink, it's considered a good throw. If it goes outside, it's not a good throw. That's the concept and then its just got other features then to tell you the direction in which you're throwing. So first of all, is there anything you dislike about it?

**Participant 3** 30:22

It's very technical. Not that I know what it's telling or what is it analysing? I understand totally. I would say this too scientific, technical, to be used in consumer electronics.

**Interviewer** 32:05

And then is there anything about it that you like?

**Participant 3** 32:13

Well, I like that the colours are informative. It's clear. It's pretty easy to understand that the green is the ideal. And this is my movement.

**Interviewer** 32:32

Okay. So the next one, then, is this one [Figure 2]. It is the second figure. Obviously, it's an Apple Watch. Again, without prompting you, do you think maybe you could roughly work out what that's trying to show?

**Participant 3** 32:54

Activity it says. I don't have an Apple Watch, by the way, I don't use it. So this is purely a guess. I'm not familiar with this. But this, to me, this could be a summary of my activity levels. Could be. So, I don't know what the arrows mean. But I could guess that they want a little bit more of the lower blue intensity training maybe? I don't know.

**Interviewer** 33:38

So, basically, the red line or the pink line, that is your total activity. So the idea is that you come around and within the day you want to complete the ring. The green is intensity. Sorry, exercise above a certain intensity. So that pink when it's like total activity, that one is activity above the certain point. This one is just time standing. So you want to spend more time on your feet. And that's what they show. Again, what do you like about it and what do you dislike? I like the clarity. This is simplified. Simple, bulky areas. It's easy to read. Once you know what you're reading [laughter]. Yeah, I like the clear. Simple is always better. Not the easiest to do [for the manufacturer to implement] but the better. Yeah. Colour is-. The red one to me is an alert So I don't know why they used red one for completing your target or whatever. That's just my personal opinion. Red is a warning light in these kind of things. I would use another colour. Yeah, that makes sense. Okay, there's one more figure, which is this one [Figure 3]. Now this is the user interface. And this is obviously the person doing the movement. So the person is wearing wearable sensors. You can see there's one on his leg, there's one on his chest. There's a few more, but you can't really see it very clearly in the figure. But then, again, without prompting, do you think you're able to work out what it's trying to show?

**Participant 3** 35:58

There's an angle of the bending forward. Minutes? No minimum, maximum. Are these repetitions, maybe? Repeats?

**Interviewer** 36:24

Yeah, you've pretty much got it.

**Participant 3** 36:30

What does the green and red mean? Is that-?

**Interviewer** 36:34

So that marker there [points to black marker on circle on Figure 3] shows that the current angle. So it records it live. So as he does the squat, the idea is that his torso angle needs to stay within that green zone. If he goes outside, either way, it'll tell them-. Well I guess that way will tell them you're bending forward the other way probably says you're leaning back. And then the graph at the bottom shows the previous reps to see where you've gone. So you can see they're all okay, and then this one [the current rep] has gone outside. And then you have the ability then to change the view and what it's measuring specifically. So at the end, you could come back and say, "Oh, that was my torso angle", but then maybe you'll have the knee angle or something. So again, with all that in mind, what do you like about it? And what do you dislike about it?

**Participant 3** 37:33

If I would be familiar, I think this would be easy to read. Pretty clear. I didn't notice that black one. That black marker there. Yeah, I'm just thinking now it seems that he's out of the target. Or the optimum angle. So I might suggest changing this view in a way that's the green is on when I'm actually on the right angle. You know, I recognised this red. So I'm kind of like there is something off. But then confused that why there is a red and green. I would turn off the green at this point when I'm out. So when-. And then vice versa. Now I'm on the green, I would turn on the green and maybe turn off the red. It's not to have them both at the same time.

**Interviewer** 38:42

So that's the figures covered. So having seen that, have any of those changed your initial thoughts about some of the things we've discussed? Would any of them be something you would consider using when you're trying to move better?

**Participant 3** 39:08

Now obviously, now that I think of that, that it's pretty clear to-. With certain types of sensors on your body, you'd be able to analyse the technique. Whether I would need it or use it, I don't know. I would want to try if I'm doing such kind of remotely guided or online exercise, or something. That might be fun. I don't think I would use it all the time. But just sometimes to check out. Swimming is something that I don't do well, I'm still learning. So that's something. I've never gone-. Nobody's really taught me the technique. Few times some been experienced has been checking out my technique and giving little tips. But I'm constantly learning and I'm constantly thinking "what could I do better?" That would be something that I would love to get. I don't want to go on the classes. I'm just too lazy to join the one month course or something like that. If somebody could offer me a device for every now and then, put it on and then giving me feedback of my swimming technique. How to make it more effortless. That would be interesting. Even that I just swim for just for my fun and exercise.

**Interviewer** 40:48

So, how do you think the general population would find the use of visualisations? Again, thinking about the customer base and just normal people, how do you think they would find the use of visualisations? Not necessarily those ones specifically [Figures 1 to 3] just in general?

**Participant 3** 41:10

Well, I think there's a lot of people who are using exercises via YouTube already. So I'm sure there's definitely a certain crowd that would be interested in like this kind of exercise? I would think so. My guess.

**Interviewer** 41:32

And then what about the level of detail that they would want? So again, thinking about the general population. Do you think they would want quite a lot of detail in the feedback? Or do you think maybe simpler? or maybe somewhere in the middle?

**Participant 3** 41:49

There's so many different-. I mean, just looking at our customer base, there are some people who want everything, and a little bit more, and some just get, like, anxiety. Too much. So I think there's a variation. Something in the middle. Just based on my experience when just comparing me and my friends, for instance, I might-. I'm not an engineer, really not. And I'm not like-. But I somehow like information. It's kind of interesting to me. And I've always like been kind of interested of my health and in many levels, so I like to measure things. But some people want less. Or more. Hard to say. So many different people.

**Interviewer** 42:52

If something like this was to be integrated into a device, how do you think the general population would respond to movement quality feedback?

**Participant 3** 43:08

I would think in general, positive, because just seeing how interested people have been about the sleep quality, for instance. I'm sure there will be a certain amount of people that are interested also in quality of movement in exercise.

**Interviewer** 43:34

When they do the exercise, and they get the feedback, how do you think they would actually use that information?

**Participant 3** 43:49

Well, I would hope that it guides them. To do it better in the future, and then could they get that-? That's kind of like teaching. Teaching you of your habits, and then the way you do things.

**Interviewer** 44:09

So do you foresee any concerns that people may have about the information that they're provided? So if we're telling them how they've moved, do you think there would be any concerns that they would have?

**Participant 3** 44:29

Again, there's people who have concerns about just about everything. And there's-. Somebody might be nervous about where the information is used. Some of them like I said, they don't want to be guided, but I don't know if they would be potentially using that device then at all. That's probably the two things.

**Interviewer** 44:55

So obviously, we're looking at where wearable technology. Where do you think people would be willing to wear a monitor?

**Participant 3** 45:11

That's what we've been trying to figure out for a while [laughter]. Well, obviously, with the 22 years of experience in [company], the wrist is one spot in body that it's well accepted to wear something, as people have been carrying wristwatches so many years. But that is a very competitive spot I would say. There's a certain group of our customers that have accepted the chest belt. But I don't know, if the device on your body is light enough and it's easy to put on and if it manages to be not bothering you during your exercise, I don't know. I'm like [unintelligible]. Your arms, legs maybe? I don't know.

**Interviewer** 46:32

Where, when you say on the arms, were thinking like lower arm or upper arm, or?

**Participant 3** 46:37

This full space [indicates from wrist up to shoulder]. I think if it's not disturbing your exercise.

**Interviewer** 46:50

So do you see, first of all, any potential barriers to using wearables? When assessing movement quality? So, do you see any limitations in using them?

**Participant 3** 47:01

They have to be comfortable. They just have to be. Easy to put on. Super comfortable. And there's so so many different levels of sensitivity. You have to be prepared to have different materials on them, because people are allergic to just about anything. You can find somebody who's allergic to any material you're offering. So a lot of challenges I think. It's not easy. It's definitely not easy.

**Interviewer** 47:36

And then the next question is facilitators to using wearables to assess movement quality. So, ways in which we can make them more appealing, perhaps, or more accessible, to get people to use them when they're assessing movement quality?

**Participant 3** 47:57

If it can be seen, if other people can see it, it has to be-. It has to look nice, anyway, somehow. It's always appreciated if the device looks cool, nice. But more important than that it has to be functional. It has to be super comfortable. And I can't underline it too much that it has to be comfortable when it's on your body. And anywhere else than on your wrist. Wrist is-. People are so used to wearing watches that they somehow accept a little bit of uncomfortable when it comes to materials, but anywhere else where it's-. Where you notice it while you're exercising. There can't be anything that is bothering the person. So those three elements: functional, comfortable, and the looks is great plus.

**Interviewer** 49:10

So the final proper question is, what would your thoughts be on introducing this in [company] products? Something like this.

**Participant 3** 49:22

Interesting. Just one word. Definitely something that I'm sure a great deal of our customers would appreciate something like that.

**Interviewer** 49:38

And then finally, do you have any other thoughts or comments on this at all?

**Participant 3** 49:49

Not really out of my head right now.

**Interviewer** 49:51

So I'm just gonna ask one more question that's not on the sheet that I would just quite like to capitalise on your experience. So in your 22 years with the company, how have you seen the market changing? And where do you foresee it going in the future?

**Participant 3** 50:13

Well, definitely, when, since I joined the company, and where we are now, we're definitely making more consumer electronics. And we're not only-. We used to be the device for pro athletes. That's where we started. And it's been coming to the mass markets during the years and the looks of the product definitely has increased its meaning. And we're more like a lifestyle product nowadays than just purely a sport product. But functions, function-wise, still very much sport focused and wellbeing focused. And I think that's still increasing, and we should be. We are. And we should totally focus on going that direction, offering these features and this product to-. More accessible to masses. Like, the big big markets, where, actually, the wellbeing is more in the focus than sports performance. I think there's so many health benefits that can be pumped up through our device. And not only the exercise, but also the sleep aspect and just the quality of life. I think so. And we're-. We used to be-. We are, we have been sports technology device. Now we are-. We're not-. We're competing for the wrist space with smartwatches. But definitely from the sports angle.

**Interviewer** 52:26

Okay. That's the end.

# **Participant 4 – Research**

Fri, 8/19 11:21AM • 52:14

**SPEAKERS**

Interviewer, Participant 4

**Interviewer** 00:00

All right, so, again, as we spoke off the recording, there's no right and wrong answers. And it's all confidential and anonymous. Basically, we're just really keen to get your thoughts and perspectives on the project and some of our ideas. So before I get started with-. Actually, I'll tell you a little bit about what we're doing first. So typically, wearable technology has been around for quite a while now. But a lot of it focuses on how much activity you do. So it's like how many steps, heart rate, how many calories, that sort of thing. What we're looking at is more about moving well. So that's what this is about, and how we get people to move better. So, before I get started with questions, would you be able to just introduce yourself, tell me a little bit about your role at the company. And then also, maybe tell me a little bit about what you do outside of the company in your spare time.

**Participant 4** 01:29

Okay. I've be been working here for nine and a half years now, at [company] and I've been a researcher. My background is that I am a Bachelor of Health Sciences. I've studied medical technology at the University of Oulu. I haven't done my Master's thesis, but I have studied it already. I'm also a certified personal trainer. In [company], I've done a lot of different research activities. So I've been collecting data, analysing data, I've been making algorithms, I've been doing other research tasks. So a lot of variation in what I've been doing. Some topics which I've been working with I've been physical activity, swimming, and then algorithm-wise, quite a large variety of features here [at the company]. During my free time, strength training is my number one sport, I'm not an endurance athlete. And never will be [laughter]. But I do health related activity. I use my bike to come to work and that sort of thing. But strength training is the thing I plan to train. Also I have been doing equine sports, so horseback riding. It's something I've done in my past. And also now but not like in a serious way anymore.

**Interviewer** 03:22

Just for my interest with the strength training, is that just general, you go to a gym and lift weights or is it competitive?

**Participant 4** 03:31

Not competitive. But aiming to progress?

**Interviewer** 03:36

So do you do your typical big three? Is it squats, bench press, deadlifts? Or do you do the Olympic weightlifting movements?

**Participant 4** 03:51

I've done both. I haven't done Olympic weightlifting in a serious way, but I've trained the techniques. And I was really keen on training more of them, but then I had an injury in my ankle. So now it's been not so much right now. But hopefully, back to that again.

**Interviewer** 04:18

So something we're going to talk around a lot is movement quality. So, just based on that term, how do you interpret the term 'movement quality'? What does that mean to you?

**Participant 4** 04:35

I think to me it's like-. My view is kind of safety oriented. So to me, it's doing things in a safe way. You know, having a good technique which makes the movement safe to you.

**Interviewer** 04:54

Okay. So what sort of things do you think help people to move well, first of all, like during everyday life? So, if you think away from sports and exercise specifically, when you're just living your everyday life, can you think of anything that might help or encourage people to move well?

**Participant 4** 05:20

Help or encourage. Well, probably knowledge, if you know what to do, if you know how you should do things, it helps you. It's terrible if you don't know how to correct. I think seeing yourself in a mirror or in a picture or in a video, for example, in everyday life, if you see yourself sitting at your desk, you probably sit better than if you don't see yourself sitting there. So it might be just an image you see from the window or something. But usually seeing makes people understand.

**Interviewer** 06:07

And then sort of the same question. What sort of things help people to move well during exercise?

**Participant 4** 06:15

I think it's the same. Knowing what what to do, like you have read about it, or somebody has told you what to do. Then seeing yourself or something. I think seeing yourself is the easiest way to make them understand what you should be doing. But also if somebody is there to tell you what you're doing wrong and how you should correct it.

**Interviewer** 06:45

Okay, so we did touch on this a little bit already. But what benefits do you think people could have by improving how well they move during exercise?

**Participant 4** 06:58

Well, yeah, safety. So they would avoid injuries. But also, of course, they could get a lot more out of themselves, the performance, would be better.

**Interviewer** 07:09

So when you're trying to improve how well you move, during exercise, what information do you think is important to receive? So, if you went to the gym and if you use your strength training as an example, and maybe you're learning a new exercise, or you're just trying to improve something you've done or change your technique. What would you want to know to improve that?

**Participant 4** 07:53

I need to understand what I did wrong. Or get an image in my head what it was that I should be doing better. Either somebody would need to tell me that during that part of the movement, you did this [some movement fault]. And that was not a good thing. Or I should see it. Or maybe I should be able to compare it to somebody who does it in a good way.

**Interviewer** 08:19

So we're exploring ways to help people understand how they move, and how they can improve the way they move, during exercise. Currently, there's no affordable, accessible and effective ways in which this can be achieved. So if you think about current options that might be available. There's things like the lab based systems where they have the camera based systems. So you have to be in a lab, and maybe that's reserved for elite athletes, or it's just simply not feasible to go and do every workout with markers stuck on your body. Also, if you have a personal trainer, maybe some people can't afford the regular rate of having that, or even someone like a physiotherapist. So if you're trying to improve better to work around an injury or work through an injury. So what we're hoping to do is use wearable technology to change that and make it more accessible for people. So we're also exploring ways that makes the data easy for people to interpret so that they can implement it safely and effectively. So first of all, can you give me some examples of feedback methods that you're aware of, that inform people of their activity, first of all for measuring how much they've done? So this comes back to some of the earlier things we spoke about with like step counting, for example. Can you think of any ways of getting that information?

**Participant 4** 10:03

So how the user gets the information?

**Interviewer** 10:05

Yes.

**Participant 4** 10:05

What do you mean? Like, I can get it from my wrist unit, like in real time? Or I can get it from my mobile?

**Interviewer** 10:15

Yeah, that sort of thing.

**Participant 4** 10:17

Yeah. Well, yeah, I'm working at [company], so I'm thinking about our ecosystem. So I can take it from a wrist unit, I can take some information, real time. I can get some notifications, which might use vibration or voice. But then I can get detailed information by scrolling the device. So it's more like when I want to see it, not when the device wants to show it to me. Also the same from the mobile phone. I can check information there. But it can also give me notifications. The web system can also give me some notifications, but mostly they are visual or vibration or voice guided.

**Interviewer** 11:10

Are there any specific examples that you can think of maybe that you might use personally?

**Participant 4** 11:18

I might use personally? I use daily activity. That's the most. I watch my sleep times, but I don't want to know it in real time. I am the kind of person who can't get that information first thing in the morning because it might ruin my day, so I need to check it later on. I don't use vibrations or voice. I always put them off on my phone, from my device. Everything, it distracts me. So I don't want the device to rule my day that much. So when I get the information, but I want to get it when I'm ready to get it, not when the device wants to give it to me. Yeah, I think daily activity is most important to me. Also training information. I check something after the exercise, but mainly I watch the details later on.

**Interviewer** 11:19

Yeah, obviously the devices have quite a lot of features. So they'll give you sleep or heart rate or daily activity. Do you use any of them yourself? Okay. So next, then, similar question. Can you think of any examples of feedback methods for measuring how well people move?

**Participant 4** 12:54

I don't think I can think about some automated ones, which would already be available. But of course, if you have a coach, you might get feedback from them. You might get some visual-. They might take a photo of you doing that. And this was how it looked. And this is how it should should look. "Could you try to concentrate on that one when you train by yourself?" Maybe the wearable could give that sort of information, even some sort of like-. Draw the version, if you did some movement, and you could do it better. But also, I think-. Well something we already give as is, when it comes to activity, we classified into different intensities. It's not the quality of the movement in that way. But it somehow gives you the information that, "Did you do a lot of very variety in your movements? Or did you just do the same thing all day?" So I think that is something which I like, because some people when they, at least when they are beginning to move, they kind of "I'm gonna go walking every day", and they're not doing anything else. So, you do the one thing and nothing else. And it might be better for your body to do a lot of variety of things. So that sort of thing could be analysed from the intensity, but also from the movement. And it will be really easy to inform that. Maybe you could do something else also.

**Interviewer** 14:49

So we've been really drawn to the use of visual aids. So we've obviously spoken about that a little bit just now. So you understand quite well what that is. Do you think-? What do you think people would think of using visual aids to help them move better? Just in general.

**Participant 4** 15:19

I think most people would find it beneficial. Some people might find it, I don't know, even embarrassing. Not everyone likes to see themselves, at least if they are not good at what they are doing, they might not want to look at those visuals or, at least, if the image is thrown to your face, you're not looking at when you want to. So it might be a negative feeling for someone, but I think it is mainly a good thing and I think it's mainly something which would help to improve.

**Interviewer** 16:05

Yeah. Do you think you personally would like something like that?

**Participant 4** 16:13

Yeah. Yeah, at least to me, it's the easiest way to understand.

**Interviewer** 16:25

So can you give some examples, possibly, of visualisations that might be used to help people during exercise? So I know you've described some possible options already, but can you think of any that might already exist?

**Participant 4** 16:43

Already exist. Well, if you have a coach, or a person or a friend, they just take a picture of you with the phone and send it to you or show it to you or take a video. Some applications are able to analyse your videos, you just have to, set your phone in a certain position and do the movement, like, in a gym, in front of the camera. So they might do some sort of analysis to you already. With that-. I think there are some applications which use wearable technology. Like, you have sensors, for example, four of them in your body, and they draw some sort of picture of you after the movement or based on the movement, but I haven't been using those so I'm not really familiar with them.

**Interviewer** 17:44

So something again, that you've touched on a little bit, you were saying about having visual feedback. And then you've said about using other types of feedback as well. When we tell people how well they've moved and what maybe they could improve, do you think that the visualisation or the visual aid would be best given in isolation? So they just get a visual image. Or do you think it would be best with other types of feedback, like audio cues, or text or vibration?

**Participant 4** 18:35

Well, that depends, of course, from the person, but also, I think it depends on the skill level of the person. Yeah. So if you have like a professional athlete, they probably just understand the image, as well as a coach might. So they just see that, "Oh, yeah, that was it, and I'm gonna fix it." But then if you have someone who is just learning, they are a beginner or they have done it, but they are not pro in it, they might find it really useful to get some sort of explanation. And then the image is just there to make sure that they understand the explanation in a correct way. But they might not. Even though if they see the image and they might see what they're doing wrong, they might not know how to correct it, or somebody might need to tell it to them.

**Interviewer** 19:35

So, what sort of devices do you think would be best to provide feedback? So if you're doing some exercise, of course, we've got the watch. We've got the app on the phone, we've got tablets, we've got a desktop or a laptop. When actually giving this feedback, what sort of device do you think would be best?

**Participant 4** 20:12

It might depend on the user. Like, somebody would like to be able to see it from a bigger screen. And so the phone or a computer could be the best. If you think that you would like to get the information in real time, then phone or wrist unit would be the best. I mentioned to you that I have a past in equine sports. So for example, in that, if somebody wants to give me advice on my riding, during my, for example, one hour training session with the horse, I'm not able to get my phone and watch it during, when I'm on the horse, because it could be a safety issue for me to take my hands off. But the wrist unit is something I can use during that. But then if I go to the gym, then of course, I'm able to use my phone because I can just put it there [in a convenient location] and watch it when I need it. But if I go, I don't know, running and I need some technique advice. I'm not really sure whether I would be willing to take my phone even if I have it with me during the session. So then I would probably just see it afterwards. So when it comes to real time feedback, I think it needs to be some sort of wrist unit or something you have really easy access, or then the phone in some sports. But then after the training session, if you want to analyse it more, then a bigger screen than a wrist unit would be really beneficial.

**Interviewer** 22:03

How much detail would you expect the feedback to provide?

**Participant 4** 22:12

In real time, not so much. Or I would just [unintelligible] that way. I would appreciate if the real time feedback would be really narrow, just focusing on the key points, because otherwise, me, for example, during sports, I'm not able to concentrate if it's like a lot of things. I'm like, "Can you just tell me the point?" But afterwards, I kinda think that I am the person who would like to get more detail. I'll be key into reading more detail, but it should be somehow formed in a way that I can check the key points, or I can check the details, but it's somehow organised in a way that I can read the details if I have time, but I can just check the key points.

**Interviewer** 23:09

So in real time, it's simple, but then maybe retrospectively, you could have that additional information that you could get into a bit. So I'm going to show you a couple of examples of visualisations that either exist in in products or that have been developed. Most visualisations that exist are around movement quantity, but some do consider movement quality. And so I've got an example of a few. I'm gonna go through each one, but first of all, I'm going to ask you what you think it shows and then we can have discussion about what you like about it and what you don't like, after I explain to you what it shows if required. So we're gonna start on the top one [Figure 1]. So I'm gonna give you a moment and, just have a look at it and see if you think you can understand what it's trying to show. And I'll leave you with that for a minute.

**Participant 4** 23:19

Yeah.

**Interviewer** 24:43

So could you explain what you think it shows?

**Participant 4** 24:51

First of all, I would I would never use this sort of image [Figure 1] myself. So it is showing the movement of the hand during a throw?

**Interviewer** 25:06

Yeah.

**Participant 4** 25:11

It's too technical for me to analyse during my free time I would say [laughter]. So that's why I wouldn't be really keen on using an image like that.

**Interviewer** 25:25

So, you pretty much already understand it. But basically, the green line is the standard we're trying to achieve. The coloured line is the throw that they actually did. So it's a wrist unit, and it's the path that it followed, and obviously then, it's got the speed of the rest of the movement. And then this pink area is a tolerance. So the idea would be is that if you're inside that pink area, the throw is considered to be good and then if it comes outside, then there's some problems corrected. Is there anything you like about it?

**Participant 4** 26:02

I like to think that, if you're like a professional athlete, or you know already what you're doing, you get the idea when your throw was a perfect one. So you get that sort of-. What I don't like is that if it wasn't a perfect one, this image wouldn't give me any information what to do better. I might, be able to say that it was the starting point, which went in a wrong way. Or it was the end. But I wouldn't be able to correct anything based on it. I would just try again and see whether the curve is a more pretty one.

**Interviewer** 26:49

So moving on to the next one [Figure 2]. This is obviously an Apple Watch. Again, I'll just leave you look at it for a moment and see if you can have an idea of what it is showing.

**Participant 4** 27:03

I've never used an Apple Watch, so this might be like really hard for me [laughter]. Okay, yeah. So I think it is showing me that my goal would be to get the full circle for everything. So there is some sort of like a goal for calories burned and active time and standing time, which I'm not really sure if I don't understand why they want me to.

**Interviewer** 27:53

Yeah. So the outside ring is your total daily activity. So the top line is your your daily activity. And the green, that one is your activity above a certain intensity. And then that one [the blue ring on Figure 2] is actually the standing time. But you pretty much got the point anyway. What do you like about it? What do you not like about it?

**Participant 4** 29:03

I like that it is easy for me to see when I reach my goal. At least if the goal is to get the full circle, but that's how I interpret it. I'm not sure whether, if I was more active, if I would reach my goal every day, for example, in the afternoon, whether they would be able to show me how much I overreached my goal, whether I was being more active than it was expecting me to be. So if that is not shown here, then I think this would be like useful until you reach the goal, but after that, it wouldn't be. What I don't like is that there are several goals in the same image. So if I, for example, get the red and the green one already, but the blue one isn't, then I would be like, "Okay, so now I'm not like -. I shouldn't be walking, but I should be standing to be able to reach that goal". So it might like, look a bit of a mess to me if they are not going like hand in hand when they are in the same picture.

**Interviewer** 30:21

Okay. There's one more image, which is this one [Figure 3]. Also, this is what we're looking at [the app interface, not the man in Figure 3]. But, just to show you that this is a sensor network. So you can see he's got a sensor on his leg, there's some on is chest, there's some that you can't actually see in the picture. But he's obviously doing a squatting movement. Again, I'm gonna just leave that with you and see if you can determine what it shows and then explain it to me.

**Participant 4** 31:29

Yeah, I couldn't use this.

**Interviewer** 31:30

The idea being is that this is how much he bends over during the squat. So as he goes into the position and his torso angle changes, the idea, he wants this black marker [on outer ring on Figure 3] to stay-. So that would move around the circle. So he wants that to stay within the green [part of the ring]. And then if he comes outside, it would say, for example, "You're bending forward", maybe there would be "You're leaning back". And then you can adjust the settings to show different joint angles and different viewpoints. And then this shows the previous repetitions. So on this one, he obviously was okay, because he was inside the threshold, and then this one is probably going to be somewhere outside. So this repetition is not sure. No, so that would be the next one. So based on that, again, what do you like? What do you not like?

**Participant 4** 32:39

What I like is that there is a visual image of the person. What I don't like is that there is no clear explanation on what he's doing wrong. Or there is "Yes, you're bending forward". But there could be a visualisation of a perfect movement, or a better one, like how you should be, and this is how you are. For example, a shadow behind the person. What I also don't like is that the repetition, where the feedback is coming from right now, is not shown here. So it kind of shows red here and green here [on the ring on Figure 3], which was like a contradiction to me. Because I was like, "Is he doing it well or not?", because it's not here. So, I would need it somehow to be here. If the analysis is not ready, I would need it to be in a shadow or something like that so it is coming here. The repetition you're doing right now. And then I just think there are-. If it analyses every repetition, I think there is too many numbers or too much information to be seen in real time. So it could be somehow hidden that there are details if you want to see them, but if you just want to do repetitions, then you could just use the image and some little information to make it better and then analyse it later on.

**Interviewer** 34:27

Yeah. Okay. So that's all of them. So have any of those changed your initial thoughts on using visualisation for feedback? Has it given you any new ideas as to how you would want it to look?

**Participant 4** 34:54

New ideas? I don't know. I've always known that I like simplicity when it comes to information. I like that there is a lot of information, but I want it to be shown in an organised way. I'm not surprised that I didn't like some of those images, because there were so many numbers and so much information coming to my face at once. It's somehow clear to me the last picture [Figure 3] that it's not only the image of the person doing the movement, but I would also want to see the perfect image there, in a shadow or in a different picture, or somehow, like, "This is how you should be doing". So even though I know how he should be doing it, it would still make it easier for me to understand if I saw it next to the-.

**Interviewer** 36:02

Yeah, I think that sounds like you're going back to earlier when I was asking you about the different types of feedback and having it in isolation against the others, is that if you have the shadow, it's focusing on the visualisation and keeping it all in the same figure, instead of then having also the texts to say what you're doing wrong, and the joint angles and so on. So it maybe just keeps it that bit simpler, perhaps.

**Participant 4** 36:34

Yeah. And even though I think when it comes to text, the text what it was in the squat picture [Figure 3] you had you're bending forward, I think there I might have needed a more of... contextual. Or maybe it could be also a spoken feedback if you have-. If you're able to hear that, but just if you tell someone that you are bending forward during a squat, it might not really be something which makes it easier for them to figure out what they're doing wrong.

**Interviewer** 37:24

How do you think the general population would find the use of visualisations. Not necessarily specifically the ones I showed you, but again, thinking about how much detail they might want, and their ability to interpret what was presented?

**Participant 4** 37:42

I think well, in an ideal world, the person could be, or would be able to choose how they get the information. Like, I like the visual. Maybe somebody doesn't. Maybe they would need it in a textual, maybe they would like to just hear it, so they don't need to see any pictures. But I think that the most important in my-. My point of view is that you get the main things in an understandable way and you might be able to choose how you want to receive the information. So maybe if you don't really find the picture useful, you might just take it off, so there would be more space for you to see that textual image if that makes it easier for you. Maybe somebody would like to see the numbers, which didn't help me. So maybe that there is some sort of-. The customer would be able to put it in a way which makes it easier for them to understand. So we don't decide for them that "This is the best information for you".

**Interviewer** 39:07

So they just have those options then?

**Participant 4** 39:09

Yeah. And then also, I like the main points to be really simple. So you can just check it and then you can act on it. But maybe if it should be, I don't know, it's a hard thing to do that. To make sure that when you put something in a really simple form, that it is understood in a way that you want it to be understood. So that could also be a problem because if the skill level of the user is from the beginner to the pro athlete, then they might understand something in a really different way.

**Interviewer** 39:54

Yeah. How do you think the general population would respond to movement quality feedback? Do you think it's a feature that they would be quite receptive of?

**Participant 4** 40:14

I think most of the people would be. I think everyone would be if they had a choice whether they want to see it or not. Like, if you could hide it. Like, "I don't want to see this in real time", and you could put it behind other information and you might want to check it later on. But if it was something that would be, for example, if I go to the gym, and I've had a rough day at work, and I don't want to think about anything, I just want to do it, then I might be annoyed that it would put the information that "Try and do your squats better" [laughter]. So I would like to be able to put it behind other information, if I'm not like ready this day to improve my technique. But it would record it anyway and I could be able to see it later on if I want to.

**Interviewer** 41:13

Okay. So how do you think that users would use the information that they're provided? So if we tell them how they moved, how do you think they would go about implementing it?

**Participant 4** 41:29

I think if we tell them in a way that they can understand what we mean, then they probably would take the hints and maybe practice a little or maybe go in front of the mirror or at home and try that out. "Okay, I'm gonna try what what did they mean that I should be doing this" in a way, but if it is somehow hard to understand, it's might just make them lose their interest towards the wearable. Like, "I don't like this device, because it tells me stuff like this and I don't understand what it means and I don't want to use this anymore". So they might just drop their interests for every feature in the watch and not just that. So it could also be kind of risky. It's not like, if the way the feedback comes isn't thought really carefully.

**Interviewer** 42:36

Do you see any concerns that people may have about the information? So I think you've touched on it a little bit then about people maybe getting frustrated? But do you foresee any concerns about something like this?

**Participant 4** 42:53

Maybe, yeah. People might have some sort of injuries, or they might have some background or some sort of limitations. And then if they try to use an app, for example, which is done for a general person, and we try to give feedback on how to do something in a safe way, and if they don't really understand, or they haven't talked to a physiotherapist on how they should be doing, and if they don't understand that they might have some limitations that other people don't, then it could be some sort of a safety thing, or it might not be good for them. So it could-. Or it should somehow be, before they start to use it, it should be like verbalised there that "If you have any sort of limitations, then then we don't recommend you use this before you are like really sure that it's not-." Because that is something we can't really track with wearables whether you have something.

**Interviewer** 44:05

Yeah, of course. So where do you think people will be willing to wear a monitor to capture their movements? So where on the bod do you think they'd be willing to wear sensors?

**Participant 4** 44:17

I think it depends on the sport. As long as it doesn't-. As long as they are not in your way. For example, at gym, it is quite easy to use them. They don't really affect your performance in a bad way. But then, for example, a swimmer. Like, everything you put into a swimmer's body and they go into the water, it makes your hands weigh more in the water, or it makes you-. It is harder to move or it might even ruin your technique in some way if you have something extra on you, then it is a really, really hard sport to measure. So it depends on sport and, of course, the size of the device, or the weight of the device, or how comfortable it is to wear and whether it is easy to put on you. If you have to, think a lot that I have to put it this way and this side up and how it is and is it wrong? Or is it right? And if you have to correct it multiple times, then you probably are not going to use it. But if it's something like a wrist device, which is easy for you to put on the right way, because there is a watch in it, so that it doesn't disturb anyone.

**Interviewer** 45:50

Yeah. Okay. Do you see any potential barriers to using wearables to assess movement? Or can you think of any thing that could sort of encourage the use of wearables for assessing movement?

**Participant 4** 46:09

Can you explain the question to me?

**Interviewer** 46:13

So do you see any potential barriers to using wearables, first of all, so any limitations for using wearables, specifically, in the assessment of movement quality?

**Participant 4** 46:28

Do you mean like, based on the person's opinion that they're not willing to use it, or?

**Interviewer** 46:34

Potentially.

**Participant 4** 46:39

Well, I think if somebody is used to not wearing anything, like not wearing heart rate, monitor or anything extra on them, or, for example, I am not used to taking my phone with me to the gym, then it is somehow a big thing for me to change my habits to take my phone with me to the gym, or maybe then wear something on you. But I think if the information you get from it is good enough, then you are willing to change your habits. But if it is something you're not really sure of whether it will be beneficial for you then, it could be like something that people are not willing to do.

**Interviewer** 47:31

Do you think there's any way then we could maybe encourage them to wear them? What sort of things could encourage people to use wearables?

**Participant 4** 47:42

I think if the information is good enough, if they find it helpful, then they will. And if it is something that you get the information from every training, and you can somehow in a long term track your performance or track that whether you are, for example, doing your squats in a better way now than you were six months ago, then it would really encourage them to use the device. Also later on, even though if they are not watching the information or using it during every training, but if they see the trend that it might encourage them to track the exercises.

**Interviewer** 48:26

And then finally. The final question properly. What are your thoughts on introducing something like this in [company] products?

**Participant 4** 48:40

I think our customers would like it. I think quite many of [company] our customers are sports enthusiastic or they want to improve better, even though there are the persons who are already really athletic, and they appreciate every information and every data. So they might be more keen to see the quality analysis of the movement, maybe not so much about how we would-. Maybe not so much the feedback we would give them but the analysis and they would like to analyse it more by themselves. And then if more of the beginners who buy [company] devices, for example, to lose weight or to start doing sports after a long break from it or something like that, then they might be really keen on the feedback or getting something out of it just to support them. When trying to make the sports habit or learning new skills or preventing injuries or something like that. But then when it comes to maybe Apple Watch or something like that. I'm not sure whether it is such a good group of people, as the [company] customers are, because they might not be so into sports, I think it needs-. The customer needs to have some sort of interest to the quality of movement to start to use them. I don't think that it's something that people start to use if they weren't interested in the first place.

**Interviewer** 50:26

Yeah. So finally, do you have any other thoughts or comments that you would want to share?

**Participant 4** 50:35

I don't know, I somehow started to think now that maybe if we have some sort of quality analysis in [company] products, it could be something that should be visualised also in [company application used by coaches]. So it could be something that we might be able to give some feedback, or some analysis of the data, but it would be something that could also be later on analysed by the coach if an athlete has one. Or a personal trainer or somebody, but you could be able to give feedback also from a real person and not just the device.

**Interviewer** 51:23

So maybe that confirms, perhaps, what the devices suggested?

**Participant 4** 51:32

Yeah.

**Interviewer** 51:32

And then maybe gives that additional detail as required and can fix things in real time.

**Participant 4** 51:38

Yeah, and also, the real person might have some more information about the person that, "Yeah, do you remember this was the week when you struggled during that exercise also and then your squats looked really poor". So maybe you were just exhausted during that week, like in all sorts, or something like they could be able to give some more explanation which the wearable is not able to give them.

**Interviewer** 52:05

Yeah, okay. Great, that's the end of the interview.

# **Participant 5 – Research**

Fri, 8/19 1:50PM • 56:06

**SPEAKERS**

Interviewer, Participant 5

**Interviewer** 00:12

So just to give a general introduction, it's very informal. So we're just gonna have a conversation. There's no right and wrong answers. We're just keen to get your perspectives and your thoughts on what we're doing. Everything is confidential and anonymous. And then just to give you an introduction as to what we're doing. So wearable technology has been around for quite a while now. But it generally focuses on things like how many steps you've done, how intense was a workout, how many calories you've burned, so it's always how much. We're looking at how well people move. Before I get started, I just want to find out a little bit more about you. So if you could just tell me a little bit about your role at the company, and then also tell me what you'd like to do outside of work.

**Participant 5** 01:18

Okay. S,o I'm hired here because of my experience of coaching, almost forty years. Several sports and really good national and international success. And my background is technical tech. Master of Science, of Technology, Process Engineering, and Automation. And after that, I educated myself at the University of Jyväskylä Sport and Exercise department and I concentrated on Sport and Exercise psychology. And after that, I went into Oulu University Educational department, and there I was concentrating on education psychology. And now I'm preparing my PhD thesis about the psychology of coaching and the deeper idea of that is to develop the athletes identity. Just like that, the physical and the mental viewpoints are included into the identity of these athletes.

**Interviewer** 02:58

And then outside of work in your spare time, what do you like to do?

**Participant 5** 03:03

Pretty much the same thing as here at [company], because I'm a behaviour scientist here. And I'm studying how people should behave to enhance their health and wellbeing. So that is my spare time actually too, but among the athletes. Here, I'm developing digital applications integrated into our sensors, the same ideas. So in a way, I'm doing the same thing in the spare time at the work as well.

**Interviewer** 03:59

So how long have you worked here?

**Participant 5** 04:02

A little bit more than a year.

**Interviewer** 04:06

Are you physically active in your spare time?

**Participant 5** 04:09

Yeah. Especially the summer times because then I play tennis 10-12 hours a week. And winter times I'm just walking, cross country skiing, and sometimes cycling at the forest.

**Interviewer** 04:30

So we're going to talk a lot about movement quality. So how do you interpret the term 'movement quality'? What does that mean to you?

**Participant 5** 04:44

It's a kind of optimal way of doing things according to your body anthropometry, according to your age, and weight, and performance level, but only-. Of course, the skills of doing different kinds of sports and I'm usually looking forward to things what I can do in the best way compared to my background, and there is of course, karate, and tennis. Both of them requires pretty good body management.

**Interviewer** 05:48

So, what sort of things help people to move well, first of all, in everyday life? So, that's just coming to work when you're in work, when you do your food shopping, and just living everyday life, what helps people to move well?

**Participant 5** 06:07

What helps people move well in their everyday life? Basically, the capability and performance level according their their age, and as far as I know, strength training is the key issue to give the mobility in its appropriate level. But of course, the aerobic performance level is important to recover from the strength training, but as older you get, the more strength training you should do. But in a way, of course, the body balance and the skills of manage your own body, for example, in case you fall down, it's very important. So, in the same time, motor skills and the strength training should be connected together.

**Interviewer** 07:36

And then, specifically, when you do exercise, what sort of things help people move well?

**Participant 5** 07:51

Complicated and different kind of-. You should not only run or bike or swim, but do many things at the same time, or to train those different things at the different days. So you have to do complicated trainings. In that way you can enhance your cognitive capacity and the cognitive capacity is connected into the motor capacity. So those two things are combined together.

**Interviewer** 08:36

So, what do you think influences how well people move during exercise? So, that's external factors that might influence how someone moves.

**Participant 5** 08:50

What influences to what?

**Interviewer** 08:53

So, what external factors might influence how well people move?

**Participant 5** 08:59

All the external? The whole of the environment? Both physical and mental challenges, there is both challenges. We should talk more about the behaviour change methods, and theories are complementary, that you have to have competence, opportunity and motivation to do something, that the behaviour happens. So, for example, opportunity means that you have to have a certain environment, which leads people to get curious to do different things. For example, you have to have a gym, or place to bike, or ski or whatever, both nature and the exercise centres. So, that is opportunity, and the capability is that, okay we have these watches and of course, [company] applications and other things, they should realise-. Cultivate your different people, that they know how capable they are of doing things in that environment which is available. So, when you combine those two things, you get self-esteem to behave in a certain manner. And I don't divide people and environment into the different entities because, all of them, they are connected to get in each other.

**Interviewer** 09:02

Yeah. So, what benefits do you think people could have by improving how well they moved when they exercise?

**Participant 5** 11:21

What benefits? They have physical and mental wellbeing benefits, motor benefits, skills, and enhancement. Social skills. Everything will become physiology psychology and sociology. Cognitive, social and psychological benefits. Word relationship theory. That is the benefit what you can get by exercising.

**Interviewer** 12:15

Can you possibly, thinking of the psychological benefits, the social benefits, the physical benefits, can you perhaps give some specific examples?

**Participant 5** 12:37

That could be a very long story. Some examples. You have a conscious, which is a core conscious, and expanded conscious. And core conscious is something that you know you are, so you are reacting in different situations. But the expanded consciousness is something that you are acting proactively. Could you tell me again the question? I will give a more precise answer. What benefits do you think people could have by improving how well they move during exercise? You gave the examples of social benefits, cognitive benefits, physical benefits, but I was asking can you give some specific examples. All those are connected into the consciousness, and consciousness, by your consciousness, you are thinking about your identity, which is goal-oriented personality. And all the goals are connected into the physical, mental and cognitive issues, in a way. But by your consciousness, you have to-. I don't remember the right word, but what does it mean when the people are behaving proactively? So, when you learn to read, you have to look at the words proactively And when you move you have to more proactively. So, it means that all of those three different things gives you the possibility to anticipate, that was the word, it gives you the possibility to anticipate different things. What is coming from the future? And because of the future, you are steering yourself and your personality into some kind of a goal. And that needs anticipating. And the anticipating contains two different possible options. One is the person you want to be and the one is the person you don't want to be. You have to avoid kind of negative results. And all of those three things helps you to understand and realise what the environment contains. And according to that you anticipate the most wanted result of your actions in the future.

**Interviewer** 16:15

So, when you do exercise, what sort of information do you think is important to receive when trying to improve how well you move? So, specifically, if you are doing some exercise, what information, what type of information, do you think will help people move better?

**Participant 5** 16:41

Okay, we have to use, again, cognitive information of the exercise session. And the cognitive information is "What kind of effect does this exercise have?" The effect is that it could be an aerobic, or a strength, or speed enhancing training, and the content of the training session, it contains the information, "What kind of effect it has in your body?" That is the cognitive side of the training system. The social side of the training session is that, can you do it among the other people? To compare yourself to the other people. That how good are you according to the other people. So, if you are isolated into the place to make the exercises, there are no one else, it is probably that you don't need to or have to, or there is no [unintelligible] of doing exercise without the other people. That is the social side of the point. And the main downside of the point is that people usually get mentally ill if their body breaks down physiologically. So you have to plan the one's exercise session so that you don't break your body, but it gives you in a good fit. And at the same time, you can have a good mental wellness.

**Interviewer** 18:53

Yeah. Okay. So we're exploring ways to help people understand how they move, and how they can improve the way that they move during exercise. Currently, there are no affordable, accessible and effective ways in which this can be achieved. So if you think of some things that might help people move better, like you could have a trainer, a personal trainer. Obviously, you have to pay and then maybe it's not so convenient, because you have to go to that person. Maybe if you've had got an injury, you'll go to a physiotherapist or something, so you have to pay them. Or maybe even if you're an elite athlete, they might have access to a lab. But of course, for most people, that's not really an option. So with the use of wearable technology, we're hoping to change this and make it more accessible and affordable for people. So we're exploring ways that makes the data we get from the devices about how people move-. We're hoping to make the data easier for people to interpret so that they can then implement it safely and effectively. So, first of all, can you give me some examples of feedback methods you're aware of to help inform people of their activity, first of all, how much activity they do, but then also how well they move?

**Participant 5** 20:18

Okay. So, this is the core question for the whole [company] strategy because [company] knows what to measure and how to measure, but [company] does not have an answer to people, when they ask from themselves,"Why to measure and why to exercise?" And, in a way, this "what, why, and how?", people-. Those questions represents the ideas that, what means your surface level, your cognitive level and your awareness of what is happening now? And what is, that you can see your heart rate graph, or you can see numerical values of your sleep, or maybe someday stretch or whatever. And the second level, why is the deep levels at the subconscious level that you have an explanation to that. Why you have bought the watch, and why you are following the instructions of training systems, and why you are following the data? How and what kind of training systems produces that data? But for the people who do not have that kind of idea, "Why should I use these watches? Because they are meaningless. The most of our life contexts, because we are not athletes or we are not keen on exercise". So, the significance of the sensors is irrelevant. They don't have the questions of why to use these activity devices. But after that, if you can raise up the reasonable doubts, for example, health and good eating and sleeping behaviour, you can tell them how, and that is a learning and innovation level of personality. So, you have surface conscious and you have deep conscious and innovation level of your conscious. So, that "why and how?" It gives you that, what you should do when after, when you are aware of all kinds of risks of your life behaviour. And in that case, how these kind of devices can help you. And then the people might get the idea of the meanings of this device. Okay, we have eyes and ears and nose and the skin feel and everything. Those are senses. There are five senses. But this watch is sixth sense. And it gathers the data from the internal environment, which we are not aware of. We are only aware of our internal environment according to our our emotions, and according to our results of the actions, for example, exercise, or not doing exercise, or, for example, after taking alcohol and eating hamburgers every day. But that is subjective information from our inside, but this [a smartwatch] gives objective information, and that is totally new thing, because we can see, by our own eyes, objective information about the environment outside, but nothing else can give objective information on inside but this sixth sense, and that brings on into the people's surface, deep, and innovation level of the conscious. That's my idea how to use these [smartwatches].

**Interviewer** 25:43

Okay. So something we've been really drawn to is the use of visual aids. So, if you think of the devices, like things that you actually see. So, first of all, what do you understand by that? If you had a visual aid, what does that mean to you?

**Participant 5** 26:07

Yeah, when you get visual information, for example, the heart rate curve or some kind of graph, that leaves you in the situation that you have to-. Let me think about it. It's the situation of self help. You have to reflect all the visual information according to your own expertise, experience, and possible guess. You have to guess what that information means to me. From those start points, you are, in a way, doing a kind of science of your own. Or it's not accurate information to you, if you don't know how to interpret those visual data. So it goes into the area of self help. And that is very dangerous to the people, because if they don't understand what that visual information means, they might do things that is not healthy for them. And we need those visual help also written or some other kind of information, which creates a kind of dialogue between the information and the user, that they can share information together about one particular information source.

**Interviewer** 28:29

Okay, so actually, moving on a little bit, because you've just brought that up. So you're suggesting that instead of just having visualisation in isolation, it needs to be accompanied with other types of feedback.

**Participant 5** 28:46

[Nods in agreement]

**Interviewer** 28:47

So, what sort of types of feedback were you thinking? Because if you think of the devices, they can give audio cues, you can have text you can have vibration. So, do you envisage a combination of all of them?

**Participant 5** 29:06

Yes. Not only the combination of those, but it has to be combined into the information what is meaningful for that certain particular person, and that person must be recognised in a way that is he old, young, short, long, thin, fat, man, woman, whatever? And including that person must have a tool that he can configure himself by self. That he can give us an information that we are not aware of what to ask from that person. So that person gives us an information what we don't know. Or we are not aware that that we should gather that kind of information from from the people because of the different cultures. I don't understand how people are thinking about themselves in Thailand or China or Afghanistan or United States. So it's a big mistake to think about people, how they think themselves in Finland, and try to figure out the questions according to that base.

**Interviewer** 30:56

So what do you think people would think of visualisations? So if you think about customers, do you think they would like visual aids?

**Participant 5** 31:10

Yeah, yeah. And combining it to the person himself. Personal information.

**Interviewer** 31:19

And then, would you like something like this? Would you personally like something like that?

**Participant 5** 31:30

Of course. Can you think of any examples where visualisations might already be used to help people do an exercise? Examples, for example big digital applications or what?

**Interviewer** 31:52

Any examples. So that would be-.

**Participant 5** 31:58

To do, for example, exercise?

**Interviewer** 32:01

Yes.

**Participant 5** 32:06

Yeah. Of course, the video clips are important to how to do, for example, bench press, or squat or whatever. That is important. But not the people who are not interested in such movements. They might be interested in chopping wood. So can we do video material for all people according to their needs. So, in this case, I would say so that we should allow the people to do their own video material. And after that, they might-. The people who are thinking the same way that "I don't like to do bench press, but I'd like to chop the wood and do some strength exercise like that." And the people are doing these sorts of videos, for example, on YouTube. It's a huge social media environment. But when it's used at [company] environment, then the same kind of people who has the same idea, they will gather together and produce their own, for example, video information to use. So we are support not to do any kind of information for the people, but the people should do information for us and the others when they are profiling themselves into the different groups.

**Interviewer** 34:12

Okay. So, focusing specifically on devices when you're giving feedback to people, what devices do you think are best for giving feedback?

**Participant 5** 34:28

Mobile phones.

**Interviewer** 34:30

Why?

**Participant 5** 34:31

It's a portable, handheld device. Watch is too small. You don't have the kind of a user interface as convenient or helpful as mobile devices. And PCs and computers are too big. You cannot take them with you every place. But handheld devices, smartphones, are best for that.

**Interviewer** 35:07

So how detailed would you expect the feedback to provide?

**Participant 5** 35:14

How the date? Yeah. So, it must be so accurate, so detailed, as the user wants it to be, because we have to create a dialogue and get rid of self help situations and interpretations of, for example, graphical information that will be totally wrong for the people who does not understand what that information contains. So dialogue is the only way to civilise and educate people to understand what [company] wants or suggest to do because of [unitelligible].

**Interviewer** 36:07

Okay, so I'm going to show you some examples of visualisations that exist. Generally visualisations at the moment are used to quantify movements. So, you'll get like a visualisation for how many steps you've done, and so on. But there are some which consider movement quality. The examples I have, are a bit of a mix, but I'm going to show you them one by one. And then we're going to have a conversation about them and try to establish what you like and what you don't like. Okay, so the first one is this one [Figure 1]. Looking at that, first of all, without prompting, do you think you could work out roughly what it shows?

**Participant 5** 36:59

So something is throwing something? Somebody's throwing something? This is not for the average people. This is for the scientists.

**Interviewer** 37:19

Yeah. So just to explain it a little bit. I think you pretty much understand it, but the green line is the 'best' throw, the ideal throw. The coloured line is the actual throw that they did, it's the path of the wrist. So you can see there as the speed information where it gets faster. Blue is where it's slower. And then it has this pink area, which is a tolerance. So the idea you want the throw to be within that, and if it goes outside, it not a good throw. What do you like about it? And what do you dislike about it?

**Participant 5** 37:57

I'm just imagining that this would be a throw of a spear or using the tennis raquet to hit the ball, for example. And I can see that the areas [pink area], not the green line, is not so in a critically effect. Okay, energy is pouring out in this area [outside the pink tolerance band] and this [the green line] is the optimal way to do, for example, the tennis hit, tennis ball hit. This picture is useful for Roger Federer, because he's going to come back [from injury] and maybe he has lost something of his motor ability. We don't know how much, but this kind of picture can give him an idea of how to learn back the best and optimal way to hit the ball

**Interviewer** 39:18

So I think what you're saying is that you you like it, but you think it's maybe reserved for elite athletes?

**Participant 5** 39:26

Yes.

**Interviewer** 39:26

Rather than for general people?

**Participant 5** 39:30

Yeah. If you want to want to give this kind of information to average people who want to learn, for example, good running skills, you should put a person into this movement line here. How the angle is doing different kind of a movement in different phases.

**Interviewer** 40:04

Yeah, so that would be an evolution. It could have the orientation maybe of the wrist.

**Participant 5** 40:12

Yes.

**Interviewer** 40:12

I'm just gonna move on to the next one then [Figure 2]. So this is obviously an Apple Watch. What do you think the picture is showing?

**Participant 5** 40:23

Maybe something like that [the red ring] being an average running training system that aerobic level is-, and the amount of aerobic running is this red one, and green one is something over the higher threshold, which enhances your aerobic capacity, and blue one is maybe some kind of heavy exercise. I'm not sure what that would be.

**Interviewer** 41:08

So, the red one is basically how much activity you've done in a day. So the idea is you want to take the ring and complete the ring. So once you go all the way around, you complete your activity for the day. The green one is doing it above or at a certain intensity. So you were right with that one. So it registers when you're doing something a bit more intense. So you have to try and close that one [the green ring]. And then the blue one is just standing time. How much time you've actually spent standing. What do you like about it? What do you dislike about it?

**Participant 5** 41:43

I don't like at all, because this is the same idea than when you try to civilise people to do more, or to eat more healthy way. These could be some kind of calories, and the people don't like to measure calories.

**Interviewer** 42:11

That is actually what it's for. The outside ring [the red ring] measures your activity through calories.

**Participant 5** 42:18

You know, the people 100,000 years ago gathering and hunting. So this tries to affect the people gathering and hunting. A sense of deep level conscious, whatever, but phase because we have totally different kinds of calls and aims of our our desires and needs. Time to calculate steps, or calories, or whatever.

**Interviewer** 42:55

Is there anything you like about it?

**Participant 5** 42:58

It's simple. But the context is wrong. You can you can use simple information by one clean spot, but the context has to consider very, very accurately.

**Interviewer** 43:14

Right, the next one, and the last one is this [Figure 3]. So, as you can see, there's a man doing some squats. He's wearing some sensors. You can see one there [thigh]. There's one there [chest]. There's a few more but the picture isn't very clear. And then this is the mobile phone app that gives him the feedback.

**Participant 5** 43:37

He can see himself here in the red bar.

**Interviewer** 43:40

Yes.

**Participant 5** 43:41

This is good.

**Interviewer** 43:42

Yeah. So if you could just explain to me what it shows.

**Participant 5** 43:50

It shows that the movement is a little bit wrong according to lower level of his back. Back should be straightforward. Up, I mean. Not in this position, because this man can hurt his back.

**Interviewer** 44:24

So yeah, you've pretty much got it. So, it obviously shows a region within which it's good, the black marker should stay inside this green section [of the outer ring] and then the red [red section of the outer ring], when he goes there is obviously wrong. And it also shows where he was for the previous repetitions. So you can see the last four repetitions showed he was in the range, so he was okay. This one is the fifth one, which obviously hasn't registered there [on the graph] yet the middle of it, but that then shows that he's outside of the range I think would get notified if you're bending forward

**Participant 5** 45:02

This person should push his knees forward a little bit and straighten the back upside and, I don't know, the sensors, if they can measure also production of power, how much? How much outcome? What is the outcome of the power level? I don't know what he's doing, maybe squats.

**Interviewer** 45:30

Yeah, he's squatting.

**Participant 5** 45:35

This person is ruining his knees, also.

**Interviewer** 45:41

Same questions. What do you like? What do you dislike?

**Participant 5** 45:47

This is very informative.

**Interviewer** 45:49

Yeah, that's something you'd like?

**Participant 5** 45:50

Yes, I like, but people should learn what all of these-. Okay, you explained to me what this means and what this means. But the point is that there is no dialogue in this picture. It means that if you touch this point from the picture, you get the dialogue. What can you see from here? What this mean? And if you touch here, you get the dialogue what this means. You have to educate people. If you have this, this is good, but you have to educate what is has happening here. And then this person should have the opportunity to give feedback to the system. And after that, the same picture gives what you should do or what could be the next thing to do to get more benefit from from this exercise?

**Interviewer** 46:55

Okay. Right, so moving on from there. Having seen those visualisations, has it changed any of your initial thoughts about using visualisations or not?

**Participant 5** 47:12

Sorry, I was just thinking to previous idea.

**Interviewer** 47:15

Okay.

**Participant 5** 47:17

No, no, no, once more.

**Interviewer** 47:20

So, has this changed your initial thoughts of using visualisations? How and why? So, having seen those, does it change any of your thoughts at all?

**Participant 5** 47:33

Actually did not change my initial thoughts, because I have been thinking of similar kinds of applications or ways to give people information. Not like the first and the second picture [Figures 1 and 2]. But you have a good idea [points to Figure 3], because I have been thinking about the same things. Same ideas.

**Interviewer** 48:07

Okay, so how do you think the general population will find the use of visualisations? Do you think it's something that they would appreciate?

**Participant 5** 48:20

Of course, they have the sensors, they can see themselves. Actually, it's a kind of avatar. A digital me. And it's a kind of self-constructed social comparison. You can compare yourself socially to yourself from that device?

**Interviewer** 49:02

And I know, we've already discussed this a little bit, but what about the level of detail and their ability to interpret what is presented? Do you think the general population would be able to process a lot of detail? Or do you think simple is better?

**Participant 5** 49:18

Yes, they can, because they say see themselves from that [points to Figure 3]. The Apple picture [Figure 2], it's not you. It's something totally different. When you can see yourself like a mirror, it's a very cognitive way to give them the information and start the dialogue.

**Interviewer** 49:40

Okay. And how do you think they would respond to movement quality feedback? So if someone bought a [company] device and they get their feedback, do you think they would like having the feedback?

**Participant 5** 49:53

Of course, because everyone wants to compare themselves to other people or to himself to be better self, to be identical of yourself. And that's why we have Facebook and Instagram and everything and picture manipulation tools.

**Interviewer** 50:22

How do you think that users would use the information that they're provided about their movement?

**Participant 5** 50:28

How would they use that? Of course, to enhance themselves. That's identity development process. To better yourself.

**Interviewer** 50:39

Do you foresee any concerns that people may have about the information they provided?

**Participant 5** 50:45

In the case, when they are living such an ideal self-bubble, which is not even close to a real sense, and when they get the truth of themselves, it could be quite a cruel situation.

**Interviewer** 51:05

When wearing sensors, where do you think people would be willing to wear a monitor on their body?

**Participant 5** 51:13

Where?

**Interviewer** 51:18

So, of course, the wrist is an obvious option. Ankle and-.

**Participant 5** 51:23

Chest and knees and maybe ankles Yeah, all the major joints.

**Interviewer** 51:32

That would be perhaps where you think you'd need them, but what about where people would want to wear them? Do you think people will be comfortable wearing that many sensors?

**Participant 5** 51:40

Yeah, why not? For example, Verity Sense is a very clever device.

**Interviewer** 51:47

Do you see any potential barriers, or limitations with using wearables?

**Participant 5** 51:55

Yeah, that's interesting.The price. And maybe it's a marketing or selling issue. For example, Whoop activity ring, you can get it for free, when you buy a coaching licence for a year. That's 300EUR. And you can get whatever kind of rings you'd like to, but the easiest way to use those devices is that when you put your clothes on and the device is there, already.

**Interviewer** 52:46

Do you see any ways in which we could encourage people to use wearables? Like a certain feature or that we could-.

**Participant 5** 52:56

Of course, self-esteem is the most important parameter to tell people that if you use these devices, you will be more attractive, you will be more efficient, you will be more accepted in social-, or whatever. Just like people are wearing something at Instagram and so on.

**Interviewer** 53:26

So is that almost like saying that there's an appeal to wearing the device?

**Participant 5** 53:30

Yeah.

**Interviewer** 53:30

As in the device looks good.

**Participant 5** 53:31

Yeah.

**Interviewer** 53:32

Interesting.

**Participant 5** 53:32

Yes.

**Interviewer** 53:34

Finally, then, last proper question. What are your thoughts on introducing this in [company] products?

**Participant 5** 53:44

Now I didn't-.

**Interviewer** 53:45

So what would you think about introducing something like what we discussed in [company] products?

**Participant 5** 53:53

To introduce our ideas to [company]? Everything?

**Interviewer** 53:58

Yeah. Well, something to assess movement quality. Do you think that it's something that could be put into [company] products?

**Participant 5** 54:08

All what you have done over there, it's important, because we are pretty much at the same duties, or we have the same kind of work for [company]. Now, I noticed that we are-. Maybe you agree with me, so we are thinking about things the same way. Those things are very important to put into [company], especiality the mobile applications.

**Interviewer** 54:41

Finally, then, do you have any other thoughts or comments or final things to say just before we finish?

**Participant 5** 54:52

Yeah. It's very important to combine object and subject issues of human life. And for now [company] has measured only objective and quantitative issues or parameters. It's a quantitative work what [company] has done so far, but the qualitative and subjective things should be connected into this historical and [company] culture, previous [company] culture. Because, it could be the second big thing after inventing the heartrate monitor, to invent emotion monitor and combining these two things together. Yep, that's it.

# **Participant 6 – Research**

Mon, 8/22 12:43PM • 31:09

**SPEAKERS**

Participant 6, Interviewer

**Interviewer** 00:11

Okay, so just to give you a general introduction, it's a very informal conversation. There are no right and wrong answers. We're just keen to get your perspectives and your thoughts on this project. All will be confidential and anonymous. And basically, what we're trying to do is we're looking at ways we can use wearable technology to basically see how well people move and help them to move better. Before I get started with the questions, I'd like to get to know a little bit more about you. So if you could just introduce yourself by telling me a little bit about your role at the company and then also what you'd like to do outside of work in your spare time.

**Participant 6** 01:05

Okay, so I work as a Senior Researcher in [company], and I work with our new prototypes and new things which are coming to the products, and help with the product line, if they have any problems. That's generally what I do. And in my spare time I, for example, now is hunting season starting, some hunting, and lots of cycling, especially with lift access. What's it called?

**Interviewer** 01:53

Mountain biking?

**Participant 6** 01:53

Yeah, mountain biking, but you use lift to go up and then down, and that's I do a lot. And other things I do is camping and staying in nature. That's my favourite.

**Interviewer** 02:13

So just as an example, the first question is, are you physically active in your spare time? So, yes. Typically, so you've said about being out in nature and hunting and mountain biking, how often do you do those sorts of things?

**Participant 6** 02:34

Well, I would say that something like three to five hours a week, at least. Sometimes it's more, can be like 10 hours.

**Interviewer** 02:48

Okay. So one of the things we're talking about is movement quality. So without prompting you too much. Someone someone's talking about movement quality, what do you think they mean?

**Participant 6** 03:08

Well, if you do some sports, there's always a technique. So, for example, in mountain biking, you should have a straight back and use your legs, not your hands or anything. So there is always technique. So I would understand it like that.

**Interviewer** 03:31

Okay, so what sort of things do you think help people to move well? First of all, during everyday life? So that would just be coming to work, doing your shopping, when you're in work? Maybe when you're with family? What sort of things do you think might help someone to move well?

**Participant 6** 03:55

I wouldn't say that move well, but stay healthy. I mean, if you always don't sit straight, or your back is always bent the wrong way, you will get something wrong in your back and then you have problems and then you cannot move. So I would say that's most important thing.

**Interviewer** 04:20

And then, specifically when doing exercise, can you think of anything that might help people move well during exercise?

**Participant 6** 04:29

Yeah. Well, I think it depends a lot what you're doing but, for example, I could think about if you go the gym, you do for example, deadlifts or something like that. If you do it wrong, you can hurt yourself. And also running and walking and cycling. If you have wrong position, you can hurt yourself and then it's like, you will go in a circle. If you have pain you cannot move, and it goes around and round.

**Interviewer** 05:06

So you've given the example of going to the gym and things like that. Can you think of any examples that might help somebody to move better when they're in the gym?

**Participant 6** 05:18

Well, I would think that you should look at your back. If your back is bent the wrong way, you can hurt yourself very easily. So I think that's the most important if you're in tune.

**Interviewer** 05:37

So can you think of any things that might influence how well someone moves, like external factors? So, I'm trying to think of an example. But you're obviously-, you're moving, whether that's in daily life or doing exercise? What influences how somebody might do a technique?

**Participant 6** 06:03

That's a hard one. I couldn't think. If, for example, if we were cycling, and you don't have sunglasses, and the sun is shining, you're in the wrong the whole time, because you're trying to avoid the sun.

**Interviewer** 06:17

So that's really, like, environmental factors?

**Participant 6** 06:20

Yes.

**Interviewer** 06:24

So what benefits do you think people could have by improving how well they move during exercise?

**Participant 6** 06:32

Well, that's same thing, like health, you can stay healthy and then you can move more.

**Interviewer** 06:41

Yeah, that's the cycle you spoke about?

**Participant 6** 06:44

Yes.

**Interviewer** 06:44

So what kind of information do you think is important to receive when trying to improve how well you move during exercise?

**Participant 6** 06:54

Well, I have had problems in my knees, so I would like to know, when my knees are wrong position. I mean, like, rotation and things like that. It would be really nice to know when I have done something wrong. For example, during the exercise, there would be some flags that you're getting when you do something wrong. Like, "Your knee is now rotated inwards. Don't do that" or "Your back is bent. Don't do that".

**Interviewer** 07:33

So we're exploring ways to help people understand how they move, and how they can improve the way they move during exercise. Currently, there are no affordable, accessible and effective ways in which this can be achieved. So if you think of some things that might currently be available, perhaps you have a coach or a personal trainer, in which case, you usually have to go to a facility, you'd maybe have to pay quite a lot of money for that regular session. Or maybe even a lab where elite athletes might be able to go there. But for most people, they can't do their training sessions in a lab, so they don't have access to those sorts of systems. And they're also very expensive. So we're looking at wearable technology, to hopefully change this. We're also exploring ways that makes the data easy for people to interpret, so that they can implement it safely and effectively. So we do some assessment using wearable technology. And then we provide feedback to people and hopefully get them to move better and safer. So first of all, can you give me some examples of feedback methods that might inform people of their activity? First of all, how much they do? So we're not talking about how well they move yet. We're just talking about how much activity they might do.

**Participant 6** 09:12

How much? Well, it's kinda-. You can do it, even now with sport watches or anything like that. You can see how many minutes you have moved and your heart rate. So you can make conclusions from that. So, I think that that side is kind of handled.

**Interviewer** 09:39

Yeah. So then, what about how well people move? Can you think of any examples that might inform people how well they've moved?

**Participant 6** 09:48

Well, you can have a goal that you want to improve; your breathing, or heart activity or something. You can choose your heart rate, but I don't know, there's not so much else.

**Interviewer** 10:09

So we've been really drawn to the use of visual aids. So, first of all, what do you think I mean by visual aid?

**Participant 6** 10:22

Well, actually, you have some mobile phone app or sport watch or something. Maybe you could see from there the exercise, how to do it.

**Interviewer** 10:35

So that's kind of what we're thinking of, is that someone would do an exercise or some activity and then they get feedback in some sort of imagery, or-. It would be visual anyway. What do you think people would think of that?

**Participant 6** 11:00

It would be really interesting. And I see that, for example, in gym, you would benefit from that kind of thing. If you could see, for example, that you are doing a set of squats or something, you would see the joint angles and your back isn't bent and things like that. And then you could see how many times you did it correct.

**Interviewer** 11:30

So would you like something like that?

**Participant 6** 11:33

Yes.

**Interviewer** 11:35

Why?

**Participant 6** 11:35

Because it's really hard to know when you are doing something wrong, especially if you're tired or something.

**Interviewer** 11:46

When you say tired, do you mean like, tired, "I want to go to sleep", or are you thinking fatigue during exercise?

**Participant 6** 11:55

Fatigue. If you think, at the gym, you have a long set of reps, for example, squats, and in the last reps you could lose your form and you don't know it. But you're hurting yourself.

**Interviewer** 12:11

Yeah. Can you think of any examples of visualisations used to help people during exercise? And that doesn't have to just be about how well they move?

**Participant 6** 12:25

Well, there could be something where, again, squat is a good example. For example, when you start to rise from the bottom, that you go deep enough. There would be some sound or something.

**Interviewer** 12:45

So if we're giving people visual feedback, do you think that would be enough to give them on it's own? So just a visual image, some sort of visual feedback? Or do you think it would be best to give that feedback with other types of feedback, like audio, like text, like vibrations?

**Participant 6** 13:11

Well, I would think that if you have visuals, you have to have big screen. So there could be problems. So maybe audio or vibration or something would be better. But of course, you have to know what that means. So maybe, all of those.

**Interviewer** 13:37

So what sort of devices do you think would be best to provide feedback?

**Participant 6** 13:46

Well, sport watch is easy because it's always with you. I don't know mobile phone, it's a bit annoying, because you have to see somehow. But I can see also separate sensors could vibrate or something. They don't necessarily need a screen.

**Interviewer** 14:14

How much detail would you expect the feedback to provide?

**Participant 6** 14:21

If it's during the exercise, you don't have time to analyse any deep insights in that moment. So it should be really short or not combining much information.

**Interviewer** 14:39

Okay. What about after? If you could look back, would you then expect to see more detail? Would you want to see more detail?

**Participant 6** 14:47

Yeah, I would like to see more detail. Of course, it should be the system that it's on should be easy to use. There's always a problem. There's too much. You can get lost.

**Interviewer** 15:02

Yeah, so I'm gonna give you some examples of visualisations that exist in products or at least some concepts that exist to date. So, generally visualisations in products today focus on movement quantity, so a lot of activity. I've got a couple of examples of both visualisations that look at movement quantity, but also how well you move. I'm just going to present them to you one by one, and we can discuss what you like, what you don't like. But first of all, so I'm going to show you-. This is the first one, this is Figure 1. Without me telling you, I'm going to give you a minute, just see if you can work out what you think it shows. And then we'll have a conversation about it. So have you got any idea what you think it shows?

**Participant 6** 16:25

Is it some-. You throw that-. Disc throwing, or that big steel thing [shot put].

**Interviewer** 16:36

So basically, what it is, is the coloured line that you can see is the path that someone's wrist has followed when throwing a ball. So they've worn a wristwatch. Or it's actually an accelerometer, it's not actually a watch. And they've thrown a ball and that colour line shows the path that the throw followed. The green line is the standard that we're trying to achieve. So that's like the perfect throw. The coloured line, you can see it has a speed. So it shows the speed of the movement throughout the duration. So you can imagine it starts off slow, and then it gets faster. And then as it gets to the furthest back position, it stops as it goes blue again. And then it comes back through and it shows it accelerates, then it gets faster. The pink area is a tolerance. So if you're within the tolerance, it's a good throw and then if it was to come out, it's a bad throw. So first of all, what do you like about it? And then what do you not like about it?

**Participant 6** 17:59

It really seems to show do you have a good throw. But I don't know how to interpret it. If I do it wrong, what I should change?

**Interviewer** 18:13

So you've got the image but you don't know then what to do with the information?

**Participant 6** 18:17

Yeah, yeah. If I go too high, should I-. What I should do then? If I were to shoot here-.

**Interviewer** 18:29

Then how do you fix them?

**Participant 6** 18:31

Yeah.

**Interviewer** 18:33

The next one is this one [Figure 2], which is an Apple Watch. Again, have a look at that and see if you can work out what you think it shows.

**Participant 6** 19:18

Well, is there some different levels of activity? I don't know what the arrows mean, but-. You're moving fast or slow or something?

**Interviewer** 19:36

So the idea on this one is that the outer ring is your total activity in a day. This green one is your activity above a certain threshold, so it's like moderate to vigorous activity. And then your blue one is your standing time. So within each hour, you have to stand a certain amount. And then the idea is that you try to complete the rings throughout the day. So once you've done enough activity or enough standing, you complete the ring. What do you like about it? What do you not like about it?

**Participant 6** 20:18

It's kind of nice. There is-. If you just know what those mean, you have lots of information in a simple form.

**Interviewer** 20:30

Anything you dislike about it?

**Participant 6** 20:43

I think it's nice, but I don't know. Is it like-. How do you activate this screen? It could mention a bit about that.

**Interviewer** 20:53

Yeah, I think you'd have to go into the device and actually look for it specifically.

**Participant 6** 20:58

Okay, that could make it that bit worse. But otherwise, it looks really nice.

**Interviewer** 21:03

Okay. And then finally, this is the last one. So this is Figure 3. This is obviously somebody doing a squatting movement. Just to indicate the relationship with that and what is shown on the mobile phone screen. The individual's wearing sensors. You can see there's one on his leg, one on his chest, and there's a couple of other sensors that he's wearing, but you can't actually see it in the figure. And then they track movement. And this is the user interface. So again, without giving you too much, what do you think that shows? Yeah, so you can see, if your back is bent.. Is there some threshold? You can see also, I think, the overall bending? Yeah, so the idea is this top section here, this black marker, you want to stay within the green zone. And then when it comes outside of that green zone into the red zone, it gives you your feedback. In this sense, it gives us as text to tell you here, for this specific movement, you're bending forward. I suppose if you went the other way, it'd be like "You're leaning back". And then it shows at the bottom, a graph that indicates what happened for your previous repetitions. So the current rep would eventually go down there once he stands back up. You can also change the angles that are being shown. So, for example, this shows the back, but maybe you can adjust that to show the knee angle, for example. And you can also change the view as well. What do you like about it and what do you not like about it?

**Participant 6** 23:23

Well, this is really interesting in a sensor point of view, but I think this gives lots of information, so I don't like that. During the exercise, you might have problems to observe all these different kinds of measurements in same time. But after the exercise, you can really see what you're doing wrong.

**Interviewer** 23:57

Yeah. Okay.

**Participant 6** 23:59

So I like it, yeah,

**Interviewer** 24:01

So would this be something you like the ability to look at it, maybe, retrospectively, but perhaps not in real time?

**Participant 6** 24:10

Yeah, that would be-. There could be some vibration or sound or something.

**Interviewer** 24:20

Would that be in place of the figure?

**Participant 6** 24:22

Yeah. And this [Figure 3] would be afterward, post-exercise.

**Interviewer** 24:29

So having viewed those figures, have any of those changed your initial thoughts on using visualisations for feedback?

**Participant 6** 24:47

Not really.

**Interviewer** 24:51

No, okay. How do you think that the general population and customers would find the use of visualisations?

**Participant 6** 25:04

I think post-exercise or if you use during exercise, you would see how to do the movement.

**Interviewer** 25:17

Any thoughts around the level of detail? And also, how able would they be to interpret the information they're given?

**Participant 6** 25:28

Well, during exercise there should be really little information feeding the person. But after exercise, there could be lots of information.

**Interviewer** 25:48

How do you think they would respond to movement quality feedback? So if we provided a facility that would allow them to get movement quality feedback, do you think they'd be quite happy to receive it? Or do you think there would be negativity about?

**Participant 6** 26:09

That depends on the people. But yeah, if you are-. You can change yourself easily, they would be happy. But if you are-. You have done something for, like, 30 years, and then somebody says that "Ah you're doing it wrong", there could be a problem [laughter].

**Interviewer** 26:35

So on that note, do you foresee any concerns that people might have about the information that's provided?

**Participant 6** 26:46

Yeah, of course, everyone is individual, even in movement. I think it might be hard to say something universal, because people have different kinds of health issues and things like that.

**Interviewer** 27:09

So if people were to wear a monitor or sensors to capture their movement, where do you think they'd be willing to wear a sensor?

**Participant 6** 27:22

Yeah, that's always hard. So people are used to using watches, and also maybe those straps around your chest. Other places are-. There is more work to put them every time. So, I think there is a really sharp incline in not to use them if you put more than maybe two things on you.

**Interviewer** 27:56

If I was to give just a couple of examples of places, maybe you could just say yes or no, if you think people would generally be happy to wear them. So you've said about wrist?

**Participant 6** 28:08

Yes.

**Interviewer** 28:09

Chest?

**Participant 6** 28:10

Yes.

**Interviewer** 28:11

Ankle?

**Participant 6** 28:13

No. Upper arm? No.

**Interviewer** 28:18

What about around their waist?

**Participant 6** 28:25

Yes.

**Interviewer** 28:27

What about on their leg? Like maybe their upper leg or like around their their calf or something?

**Participant 6** 28:38

That depends a lot. Does it need to be on the top of the clothes? If it needs to be under the clothes then no. Otherwise, yes.

**Interviewer** 28:50

Okay. So do you see any potential barriers to using wearables to assess movement quality? So that's any limitations to using wearables for this?

**Participant 6** 29:03

They should be really easy to use. Easy to put them on.

**Interviewer** 29:10

And what about ways in which we can encourage people to use them?

**Participant 6** 29:18

If there is high-. Like, you will get something precious, people will do more. So if they will get really precious information, they will put, even if it's more work. Under clothes, some sensors.

**Interviewer** 29:42

So final question. Final proper question. What are your thoughts on introducing something like this into [company] products?

**Participant 6** 29:53

Yes, it iwill give more information and I think it's needed.

**Interviewer** 30:03

And then finally, do you have any other thoughts or comments at all?

**Participant 6** 30:23

Yeah, I don't know. It would be-. It's always like, if you have more sensors, you will get more information. But people don't easily use more sensors.

**Interviewer** 30:38

So it's finding that sweet spot of "How many is enough to get good results without being too many?"

**Participant 6** 30:46

Yeah, and the sensor itself doesn't do anything if you have to have some data analysis, and then feedback to the user. That's really important.

**Interviewer** 30:59

Yeah. Okay, great. That's the end.

# **Participant 7 – Software Architecture**

Tue, 8/23 4:22PM • 32:01

**SPEAKERS**

Interviewer, Participant 7

**Interviewer** 00:12

Okay, so again, as spoken off the recording, there's no right and wrong answers. We're just keen to get your perspectives and thoughts on things related to our project. Everything is confidential and anonymous. So anything that you say that might identify you will be omitted from any transcripts and the recording will be kept on a locked laptop, as will your consent form. So, before I get started with the questions, I'd first like to get to know a little bit about you. So would you please be able to introduce yourself by telling me a little bit about your role at the company, and then also what you do outside of work in your spare time?

**Participant 7** 01:13

Okay, so I've been working for [company] about six years now. And, actually, I can't tell you much about my current role, because I just moved to a new team. And I don't yet know exactly what I will be doing. But it will have something to do with architecture, so software architecture. That's what I've been doing for the last few years in a different team. And outside of work, I like to try to be active. So I walk quite a lot and occasionally run. I used to do a lot of cycling, but recently, I lost motivation. Basically, try to be active, and also I like to read a lot.

**Interviewer** 02:16

So a lot of what were looking into, and what we're going to talk about is the concept of 'movement quality'. So, just based on that term alone, what does 'movement quality' mean to you? How do you interpret 'movement quality'?

**Participant 7** 02:37

Quality. Related to what?

**Interviewer** 02:42

That's kind of the thing. So how would you interpret that? When we're talking about-. I'll give you some context then. When we're talking about being physically active, whether that's through formal exercise, planned exercise or not, What do you think movement quality refers to?

**Participant 7** 03:05

I guess. for me, it sounds like, if I'm being active, I'm doing some kind of movement, is it a good quality related to my health, and does it improve my health? Keep me in good shape.

**Interviewer** 03:29

So what sort of things do you think help people to move well? First of all, during everyday life, so you're coming to work, you're in work, maybe you're at home, and you're cleaning the house or you do your food shopping. When you're just living everyday life, can you think of anything that helps you to move well?

**Participant 7** 04:01

I guess, some kind of reminders during the work day to get up and move a little bit.

**Interviewer** 04:08

Is that from technology?

**Participant 7** 04:11

Yeah, could be. Could also be another person. But I was thinking mainly about technology.

**Interviewer** 04:18

Okay. And then specifically, then, what about when doing exercise? What do you think might help you to move well?

**Participant 7** 04:27

If the goal is to do some kind of planned exercise then there should be some kind of guidance from maybe a watch or something.

**Interviewer** 04:38

Are you familiar with any methods of guidance during exercise that might help someone move well? Do you have any examples specifically?

04:48

Obviously, I'm used to [company] devices and the target-based exercises there.

**Interviewer** 04:55

Okay. So subtly different question. What do you think might influence how well you move during everyday life and also during exercise? So possible external factors that might influence how you move.

05:17

One thing that comes to mind is, if you think about commuting, if the city infrastructure is not good, for example, cycling, that might affect-. So in Oulu, for example, there's very good cycling road network, so I guess it will be easy to commute by bike here.

**Interviewer** 05:45

Yeah. Okay, so what benefits do you think people could have by improving how well they move?

05:55

Health benefits, of course. Maintain good health.

**Interviewer** 06:02

So, when you are doing exercise?

**Participant 7** 06:10

If I'm doing exercise.

**Interviewer** 06:11

If you're doing exercise, or not even specifically you, just anyone, what information do you think is important to receive about how well you move? And this is specifically during exercise?

06:32

First thing that comes to mind is heart rate guidance, which we have on the [company] devices. But when I think about the word 'quality of movement', it doesn't really sound like that, to me. It's more like if I'm doing some sort of movements in correct form.

**Interviewer** 06:59

Yeah, that's kind of the way we're thinking. So, on that note then, if you're doing some exercise, and you want to do with good form, what type of information would you want to get back to encourage you to do it with good form?

07:23

To have to know what the good form looks like.

**Interviewer** 07:28

So that's some sort of target that you're you're aiming to achieve?

**Participant 7** 07:32

Yeah, and then how is your movement compared to that target.

**Interviewer** 07:40

So we're exploring ways to help people understand how they move, and how they can improve the way that they move during exercise. So currently, there are no affordable, accessible and effective ways in which this can be achieved. So, just to give some examples of what you might have available to you already, things like having a coach or a personal trainer. They're not necessarily readily accessible. So, you might have to go to a facility to get access to that. There's usually an hourly rate or some ongoing fee that might then be too expensive and price you out from being able to use those services. Same with also, if you have an injury and you're coming back from that injury, something that might help you move better, you have a physiotherapist, for example. But again, you have to use that facility and it costs a lot. You may even have like a lab based system. So, you go to a lab and you have a camera based system. But that then is reserved to elite level athletes. You can't do your everyday training sessions in a lab, so it's not practical. So what we're looking to do is use wearable technology to change this and make it more accessible, make it more affordable. But also we're exploring ways that people can take the data that's collected from the wearables and make it easy for them to interpret so that then they can implement it safely and effectively and improve the way they move. So, first of all, are you able to give me some examples of feedback methods that you're aware of that help inform people have their activity? First of all, how much activity they do. So this one isn't specifically looking at movement quality. It's looking more at how much activity they do.

10:01

Well, of course, there's these activity trackers, which we also have on these [company] devices. What else? I don't know.

**Interviewer** 10:16

Maybe you could give me a specific example, then, of the activity trackers? Like, what did they show specifically?

10:28

Well usually there's targets for certain level of activity. You get a percentage of how much you achieved that. Maybe reminders if you also sit too long. Stuff like that.

**Interviewer** 10:41

And then coming back to what we're talking around, talking about movement quality. Can you think of any feedback methods that might tell someone how well they've moved?

10:57

I don't know if it's available on any devices, but one thing that comes to mind is, for running, the devices might be able to estimate somehow, maybe your running form or something.

**Interviewer** 11:18

But you don't know-. That's like something you've theorised rather than something you're aware of, but they do exist?

**Participant 7** 11:28

I don't know. They might.

**Interviewer** 11:30

So something we've been really drawn to is the use of visual aids. So before I move on, what do you understand by that? How would you interpret what a visual aid might be?

**Participant 7** 11:50

It could mean a lot of things. Are we talking about during exercise?

**Interviewer** 11:57

Specifically, in this case, probably, but doesn't have to be.

12:08

If it's in a gym or something, then there could be a video screen showing maybe some guidance and maybe be showing you as well. Maybe side by side, something like that.

**Interviewer** 12:22

So basically, when we're talking about visual aids, we're thinking of some sort of feedback that uses imagery or video, something that you see to get the feedback rather than other methods that might exist. What do you think people would think of having visual feedback to tell them how they've moved?

**Participant 7** 12:52

I think it would be useful.

**Interviewer** 12:54

Okay. Do you think you personally would like something like that?

**Participant 7** 13:00

Yeah, probably.

**Interviewer** 13:02

Why? Why would you like that do you think?

13:08

I might be interested, in how's my running form. Maybe it could be more efficient or something?

**Interviewer** 13:19

Can you think of any examples of visualisations that might be used to help people during exercise? I know you suggested possibly a video type of thing, but maybe any others?

13:35

If we're talking about outdoors, then that's obviously not going to work now. It depends so much on what kind of movement we are talking about, what kind of visual aids would help them. Maybe if we talk about running, for example. Maybe, for example, if you're going for a certain cadence and you're beyond, or going too fast, you could get some kind of indication on the watch.

**Interviewer** 14:09

So, if someone was doing exercise and we wanted to give them feedback on how they've moved, do you think it would be best to have that visual feedback in isolation? Or do you think that it would be better to have the visualisation with some additional type of feedback? So sound, vibration, text Do you think it'd be better on its own or-?

**Participant 7** 14:44

I think maybe combination.

**Interviewer** 14:47

Okay. Why?

**Participant 7** 14:52

For example, if we stay with the running example, you don't necessarily look at your watch all the time. So you might want to hear a notification, or feel it vibrate and look what's going on.

**Interviewer** 15:07

Okay. So if we were to give visual feedback, or not even necessarily visual feedback, but if we're going to give feedback, what devices do you think would be best to give that feedback?

**Participant 7** 15:24

During exercise?

**Interviewer** 15:27

Just in the general sense of having feedback about your exercise. So there may be a scenario where you want it during exercise, possibly even have it after exercise once your session has finished. So there might be a preferred device for each stage? Or you might just want one throughout?

**Participant 7** 15:55

After exercise, I think a mobile phone would be best because everyone has it, usually, with them, and it has a big enough screen to actually show some relevant or useful information.

**Interviewer** 16:10

What about during exercise?

**Participant 7** 16:12

Probably their smartwatch.

**Interviewer** 16:16

So if you were to do some exercise and you wanted to get feedback, how much detail would you expect to have?

**Participant 7** 16:27

I think it depends on the type of movement and also the level of the of the person or how experienced they are.

**Interviewer** 16:38

Could you expand on that possibly?

16:42

If I'm a beginner, I think I would need, want, as little information as possible that's actionable to me right now. And then if I'm more experienced, I want more detailed information.

**Interviewer** 16:59

Okay. So what I'm going to do now is I'm just going to show you some examples of visualisations that exist. Typically, visualisations are used, or have been used, for quantifying movement, just because that tends to be what most devices seem to focus on right now. It's like, how many steps and how many calories you've burned and so on. What I've done is I've tried to get a bit of a mix. So what I'm going to do is I'm going to show you this-. There's three visualisations. I'm going to show you each one, one by one. I'm going to let you look at each one, see if you can work out what it's trying to show, and then we'll have a conversation about it, whether you like it or whether you don't, and what you like and what you don't. So this is the first one [Figure 1]. So I'm just going to leave that with you just for a minute and see if you can work out what you think it's trying to show. Have you got any idea what you think it might what it might be trying to show?

**Participant 7** 18:26

I'm guessing throwing something? So maybe frisbee golf?

**Interviewer** 18:34

So, basically, the the coloured line is the wrist trajectory during a throw of a ball, specifically, in this case. So the coloured line is what you actually did. The green line is what we are trying to achieve. That's our best throw, our gold-standard. And then this pink area is a tolerance. So, effectively, if you stay within that pink area, it's considered a good throw, and then if it deviates from it, you have some issue that needs to be corrected. The coloured line also then shows you how fast the wrist was moving at the specific point, so you can see it starts off slow and then it accelerates so the speed increases. When you reach the back position, it stops and it goes slow again and then as you accelerate it speeds up and goes through. What do you like about it? What do you not like about it?

19:47

I'm not sure if I like it or not, but what I noticed is that this pink area is very large and it's kind of hard to see-. It looks like it could be a really large range of different movements and I would think the optimal would be narrower.

**Interviewer** 20:17

Yeah. Is there anything that you like about it at all?

**Participant 7** 20:24

I think it's quite understandable.

**Interviewer** 20:28

Okay. The next one is this Apple Watch [Figure 2]. Again, just take a moment. See if you think you can work out roughly what it's trying to show. So what do you think it shows?

**Participant 7** 20:48

I'm guessing it shows your level of different intensities of activity?

**Interviewer** 20:55

Yeah, so what this shows is that the outside ring, the red one, is your total activity. The green one is activity above a certain threshold. So once you've reached a certain intensity. And then the blue is standing time. And then the idea of is, as you do more activity, or spend more time standing or so on, you try to complete the ring. So that's your goal for the day, is to try and complete the ring. Based on that, what do you like about it? What do you not like about it?

21:38

I would have guessed that the red one, it would be high intensity. So at first glance, I didn't understand what it means.

**Interviewer** 21:49

So perhaps, is that because of colour selection?

**Participant 7** 21:54

Maybe. Maybe the colours.

**Interviewer** 21:57

Okay. So you were thinking, perhaps, the blue would be low, the green would be medium, the red would be high?

**Participant 7** 22:03

Yeah.

**Interviewer** 22:04

Okay. Is there anything you'd like about it?

**Participant 7** 22:07

I think it's quite obvious that you want to complete the circles. So it's pretty intuitive.

**Interviewer** 22:20

Okay. And then, the final one that we're gonna look at is this one [Figure 3]. So this guy is doing a squatting movement. He's wearing a couple of wearable sensors, you can see the one on his leg and see there's a chest strap. And there's a couple more that you can't actually quite see from this image. And then this is the app user interface that he uses to get his feedback. What do you think it's trying to show, first of all?

**Participant 7** 23:06

I have no idea. I mean, obviously, the person's image here is he's showing what the movement is like, but then probably some of it is some kind of target, but I don't know.

**Interviewer** 23:24

Okay, so this is this figure here, in the middle of the circle is a real time depiction of what he's doing. The circle then tells him, in this specific sense, his torso angle. So there's a black marker there. So while he does the exercise, that black marker will rotate around the circle, and he wants that black marker to stay within this green section. So as he bends forward, that marker will move, and he wants to ideally stay inside that green section. It's come outside, so that's telling him "You're bending forward", and that he needs to change his technique and stay within the green. The bottom portion shows him his previous repetitions and the angle to which his torso got to during that movement, and then these show the thresholds. So you can see his previous four repetitions were considered to be good. This one, because he's gone outside of it, he'll get feedback that will indicate that was not good. What do you like about it? What do you dislike about it?

**Participant 7** 24:46

I dislike it because I didn't understand at first what it means, but now that you explained it, it seems clear. And probably means that when, before you will navigate to this screen, there's something that tells you what this is about. So it's about your torso angle, was it?

**Interviewer** 25:07

Yeah.

**Participant 7** 25:07

So having that context would help a lot in interpreting this.

**Interviewer** 25:16

Is there anything you'd like about it specifically?

**Participant 7** 25:20

Now that you explained it, I think this angle indicator is quite-. It's good. It shows you about where you are now and where you want to be.

**Interviewer** 25:35

Yeah, cool. But perhaps, just at first glance, there's quite a lot going on and it's maybe not as intuitive as something else?

**Participant 7** 25:44

Yes.

**Interviewer** 25:45

Okay, so, have any of those, having seen them, changed your initial thoughts on using visualisations for feedback?

**Participant 7** 25:58

Not really.

**Interviewer** 25:59

No, okay. How would you think the general population would find the use of visualisations?

**Participant 7** 26:10

What is the general population?

**Interviewer** 26:13

So, your average person. I'll go a bit more specific and I would say, prospective [company] customers. So if I just narrow that in a little bit for you, we're thinking maybe people who want to be active or are active that would buy a [company] device.

**Participant 7** 26:42

Yeah. I think they would like it. I can imagine that someone, maybe someone who was was interested in yoga or something like that would find this very interesting. And maybe also some more experienced athletes in sports where the form is critical.

**Interviewer** 27:12

Do you think that it would be more experienced athletes that would benefit from this moreso than somebody trying to learn the movement for the first time?

**Participant 7** 27:23

I think both.

**Interviewer** 27:25

Expanding on that a little bit then, have you got any thoughts on the level of detail that they might want, and how easily they would be able to interpret the data?

**Participant 7** 27:39

Like I said before, more experienced athletes and people, not necessarily even athletes, but people used to [company] devices and using mobile devices and so on, for sports and movement, would probably want more more detail.

**Interviewer** 28:12

How would you think the general population would respond to movement quality feedback? So if we provided them with something, do you think it would be well received or negatively received?

**Participant 7** 28:26

I think positively.

**Interviewer** 28:29

Okay. If we give users feedback on their movement quality, how do you think they would use that information?

**Participant 7** 28:43

To help them learn the movements better. To improve their performance in sports. Yeah, that's it. I can't think of anything else.

**Interviewer** 28:59

Do you foresee any concerns that people may have about the information they receive?

**Participant 7** 29:10

Maybe if the feedback isn't accurate, then it might guide you to do the movements wrong. That could possibly lead to an injury or something.

**Interviewer** 29:30

Where do you think people would be willing to wear a monitor to capture their movements? So where on their body do you think they'd be willing to wear a sensor or sensors?

**Participant 7** 29:44

The wrist, obviously, because the watch is already there. Maybe as earbuds, especially if they would also work as well for playing music at the same time.

**Interviewer** 29:58

Yeah, that's interesting

**Participant 7** 30:02

Maybe a headband. Maybe a band around your upper arm, legs, maybe?

**Interviewer** 30:09

Where on the legs do you think?

**Participant 7** 30:11

Maybe it would be mounted on the shoe, or maybe around the ankle.

**Interviewer** 30:18

Okay. Do you see any potential barriers to using wearables to assess movement quality? Any limitations?

**Participant 7** 30:27

I can't think of any.

**Interviewer** 30:31

Okay, what about facilitators? So ways in which we could encourage people to use wearables to assess movement quality?

**Participant 7** 30:43

What do you mean?

**Interviewer** 30:44

So can you think of anything that might make it more acceptable for people to wear wearable technology when they're assessing movement quality?

**Participant 7** 31:02

The sensors should be small and nice looking, so that they're not-. Others wouldn't be able to see so clearly that you're wearing some kind of device.

**Interviewer** 31:27

And then the last proper question is, what are your thoughts on introducing something like this into [company] products?

**Participant 7** 31:37

I think it's a good idea.

**Interviewer** 31:40

Okay, so, before we finish, do you have any final thoughts or comments at all?

**Participant 7** 31:49

No, not really.

**Interviewer** 31:51

Okay. That's the end.

# **Participant 8 – User Experience (UX)**

Thu, 8/25 6:47PM • 1:01:26

**SPEAKERS**

Participant 8, Interviewer

**Interviewer** 00:47

Okay, so just before we get started then, just to give you a general introduction. It's a very informal conversation. There's no right and wrong answers. You do not have to answer every question. You know, you might not have an answer for every question, so that's fine too. Basically, we're just keen to get your thoughts and your perspectives on some of the ideas that we're going to discuss and on the project. Everything, as I say, is confidential and anonymous. So just to give a bit of context about what we're doing specifically, without giving you too much detail ahead of time. What we're looking to do is look at movement quality, and basically how we can use wearable technology to aid in the assessment and measurement of movement quality. So before I get started with the questions, would it be possible, please, for you to tell me a little bit about yourself? Specifically, your role at [company] and also a little bit about what you like to do when you're outside of work in your spare time.

**Participant 8** 02:11

Okay, so in [company], I've been working here for five years now, first as a UX [User Experience] designer, but now I've been working as a usability specialist in our UX team, and that means I'm doing a lot of these kinds of user research, and interviews, and questionnaires and studies. And we are looking, like, doing research about our users and possible future users, and testing our software and devices with the end users. Yeah, so I'm collecting data and analysing it and reporting lot. And interviewing also.

**Interviewer** 03:00

Just before we move on to the spare time question, what sort of demographic are you focused on mostly? What sort of ages? What types of people?

**Participant 8** 03:15

Depends a lot about the scope of the research. So, if we are testing a software, or piece of software, it doesn't matter the demographic so much, but if we are testing special sports, or sport feature, I'm looking for participants that are in in our, like, age and sex that usually use those which are in [company], something around 30 to 50 years old, and mostly males. Maybe like 70% males and 30% females, etc. So it depends a lot, what we are-, what we want to study. So a lot of but if we are testing software, it doesn't matter so much usually, if it's just like basic usability test, so then the participants can be quite wide range, so, well depends also that what we are studying. And are also on my free time I have dogs and I'm doing agility [dog agility courses] and then I also go riding, horse riding, and CrossFit and skiing and running and all kinds of sports. And being with family also, I have two kids.

**Interviewer** 04:42

Okay, cool. When you say doing agility is that like with the dogs? Yeah, I know what you mean, in the in the-. So going on to the questions properly then. So we're going to talk around the term 'movement quality'. How do you interpret the term 'movement quality'?

**Participant 8** 04:45

Like, when I'm personally doing exercise or just theoretically?

**Interviewer** 05:29

Generally. I suppose it depends on your interpretation of it, but I suppose both in a way.

**Participant 8** 05:37

Movement quality. So I guess-. I'm not sure what do you mean? Can you specify a little bit?

**Interviewer** 05:53

I suppose then, if-. I've tried to do it without giving the answer really. If someone was having a conversation with you, and they were discussing the quality of the way that you move.

**Participant 8** 06:11

Okay, okay. Maybe, well, I think it's quality-. Maybe that you are moving, exercising, maybe, or moving in the right pace and right way, so you are not doing too much or doing it wrong, like physically, movements from if you're at the gym or doing cross training, so you are doing the exercises correctly. And the weights and all the pace and speed are good for your fitness level, not too high, or maybe not too low, also, so that you won't get injured, for example, it's not too heavy. That's good quality, that there is no injuries and it's not too hard for you, your physical state.

**Interviewer** 07:07

Okay, so what sort of things do you think help people to move well? First of all, during everyday life. So we're thinking, you go to work, you do your food shopping, you're, maybe, playing with your kids, your dogs. When you're just living everyday life, what might help you to move well?

**Participant 8** 07:36

Well, that it's easy. Easy to do. Like, it's easy to move. There is no limitations. I mean, in your everyday life, there is this things like children, etc. So it's not easy to go for a walk or for a jog, but-. So easy access. But also, if you are at the gym, or doing cross training, or something like that, somebody is advising you or you have some training plan, maybe. That also makes it easier to follow some advice.

**Interviewer** 08:22

So slightly changing the question, what do you think influences how people move? And I think you've kind of touched on this a little bit when you said about being busy, but can you think of anything else that might influence how you move?

**Participant 8** 08:38

Oh, my every day, rest of my everyday life, like, how busy I am at work and at home? My kids are very small. So I have to take the time [to exercise] whenever I can. Yeah, but also, that it's easily accessible. So, I don't know, people are maybe going somewhere far away to do exercise, but for me, currently, it's, I step out of the door, and then I go for a run or skiing. So it's as easy as possible. And then the gym is quite near so it is easily reached. And it's only five minutes away. And then maybe also that I'm motivated enough. So I like the things I do, so then I also find time when I'm motivated enough. So I'm not forced, or somebody told me to do something. It's what I want to do myself.

**Interviewer** 09:48

Yeah, that makes sense. So what benefits do you think that people could have by improving how well they move specifically during exercise?

**Participant 8** 10:02

Well, to avoid injuries from the exercise, but also from outside of exercises. If you have more strength, strength and more durability. And I have better energy, like, more energy in my other life [daily life outside of planned exercise], if I'm exercising well and more being correctly. So I have energy. And then also it's a positive side to my-. It clears my head also. So that's important side of moving well.

**Interviewer** 10:54

So we're going to start tapping into your expertise a little bit now. When you are trying to improve how well you move during exercise, what kind of information do you think is important to receive?

**Participant 8** 11:11

If I think about my CrossFit or cross training exercise, because that's like what specific sport-. Well, it's important that the instructor told me how to do the movement, and how not to do the movement, because my back is aching easily. So I want to know how to avoid to injure my back, then how to do the movements correctly and what are the correct weights, so I don't put too many weights. Too much, or too little. And also, if I go by myself, without the instructor, I have some paper or the instructions about the movements and why I have to do these kinds of things. Also the motivation behind that exercise. So, why I need this. Is it like for gaining more strength? Or aerobic exercise or?

**Interviewer** 12:24

So, we're exploring ways to help people understand how they move, and how they can improve the way that they move during exercise. Currently, there are no affordable, accessible and effective ways in which this can be achieved. So if you think about some of the examples that exist already, there's things like personal trainers, where you have to go to a facility in most cases, you have to pay an hourly rate or some ongoing fee. Maybe if you've got an injury, you have to pay a physiotherapist, and again, you have to go to that place. Or even if you're an elite level athlete, then you might have lab access, but for most people, they don't have that lab access or that facility access that they can go to for every training session or for a regular movement assessment. So with the use of wearable technology, we're hoping to change that a little bit. So we're exploring ways that we can use wearable technology but also ways that makes the data easy for people to interpret, so that then they can implement it safely and effectively and improve the way they move. So first of all, before, I'm going to show you some some examples, but are you able to give me some examples that you're aware of feedback methods that might help inform people of their activity? First of all, how much activity they do? So the quantity of exercise rather than movement quality at the moment.

**Participant 8** 14:13

Okay, with wearable or in general?

**Interviewer** 14:16

In general, so could be either.

**Participant 8** 14:20

Something else than watch? Well, of course, our [company's] running programmes or you have the running-. In [company] watch, that I'm having an exercise and it tells me that you have to go at this pace and this speed and you have now gained so many kilometres, so that tells you that now you have been running for five kilometres. And also the heart rate tells me that it's too high. Okay, I have to define myself what's my heart rates zones and when they are too high and when they are too low. Probably there's no low enough, but anyway. But I don't know, I'm not using any kind of training programmes with wearables or with my mobile phone with tells me that-. I don't know what else.

**Interviewer** 15:31

Yeah. So to be honest, those are common examples that have been reported previously. Are you aware of any [examples] for then assessing movement quality, rather than quantity?

**Participant 8** 15:50

What did you mean by being aware?

**Interviewer** 15:53

So we're talking about feedback methods. So ways in which people can get information about how they're doing activity. Can you think of any examples for movement quality, rather than quantity?

**Participant 8** 16:09

Well, when I'm cycling with the Wattbike, it tells me that how well I'm pedalling. So is it correct in both legs, both feet? And is my power good enough? So that's kind of telling me about the quantity of that cycling. So that's what I've been using.

**Interviewer** 16:31

That's really good, actually. Yeah, that's the feature with the peanut [Wattbike pedal profile visualisation feature], yes?

**Participant 8** 16:38

The peanut has to be quite wide, not like an eight. So it tells me if I'm eight [peanut visulaisation shouldn't look like a figure of 8], it's not good enough. Movement is-.

**Interviewer** 16:50

So that's a good example, actually, to lead on to the next questions, because something we've been really drawn to is the use of visual aids. So, forgive me, because some of these questions, particularly for someone in your position, are quite simple questions. But what what do you understand by that? How do you interpret visual aid?

**Participant 8** 17:28

How I interpret like, can you-? I didn't understand your question.

**Interviewer** 17:36

So that the term 'visual aid'. What does that mean? What would you expect it to be?

**Participant 8** 17:53

Okay, yeah. So it has to be easy enough to understand in the moment, because I'm like, on the Wattbike, I'm cycling, and if it's too complex, I do not have time or energy to start analysing it. So it has to be the-. The visual effect has to be very clear. Very easy to understand. The usability has to be, even if I'm a professional, is still like, in that moment I'm exercising and doing sports, I do not have brain cells to start analysing what I'm doing. If I have the information when I'm on the bike, or during the physical movements. So with one glance, I have to analyse it easily. Did I answer your question?

**Interviewer** 18:52

Yeah, and then some, actually. We'll come on to a little bit more of that in a bit. But first, what do you think that people would think of using visual aids to help them move better?

**Participant 8** 19:15

Well, again, to come to that fact that they have to be easy to understand and easily approached. Like, easily accessed. So when I'm doing exercise, I don't want to have anything extra. That's what I've been doing in my latest study. So people have a lot of gadgets and stuff in their hand and in their pockets, smartphones, etc. So when people are exercising or moving-. Some people probably, but most of the people I've been interviewing, they don't want to have these beeping machines in their hands and they want to have some peace and quiet. So if I have something that shows me my exercise, I want to have it like quick and fast and easy and simple enough, so I don't have to-. I don't have time to scroll or tap and read the manuals, etc.

**Interviewer** 20:18

So expanding on what you said there a little bit, do you think that type of feedback, visual feedback, would be best provided in isolation, or do you think that it would be best delivered with other types of feedback? So with vibrations, with audio, with text?

**Participant 8** 20:42

Well, it depends a lot what kind of device you have. Like, screen size, probably. But I prefer if it, like, isolation, but I'm not sure. Depends. When I'm running, the vibration is good feedback, because I do not have time and energy to start [unintelligible] my devices or wearables. So a little bit depends of the situation. But I would prefer the isolation first, and then maybe start combining, or have several. But with very careful-. Lifestyle. Because the situation you saw kind of sensitive or so-. There is so much going on in the physical movements. So that's why it's difficult to have more. If you have to have vibration and sound and visual, some images or something. So, too many things going.

**Interviewer** 21:54

So what sort of devices do you think would be best to provide feedback? And I know you've just touched on that a little bit. But if we're thinking with [company], obviously, there's the smartwatches, there's the apps as well, we've got the [company apps]. There's a desktop facility as well, so that you can access that on your computer or your laptop. What sort of devices do you think will be best to provide feedback for getting movement quality information?

**Participant 8** 22:33

Do I want the quality information during the exercise? Or afterwards? Because that also differs, if there is like-.

**Interviewer** 22:42

Yeah, so if you think both, then, perhaps you could explain the context in which one might be better than the other?

**Participant 8** 22:54

Yeah, quality of movements during the exercise, so during my-, when I'm moving actually. Personally, I don't like to carry my mobile phone when I'm on the run, or, you know, all my smart devices beeping in my pocket, and kids calling me and somebody's sending emails and all kinds of chatting, chat applications beeping, so I don't want to carry it. And during my exercise, I don't want to gaze it all the time. And that's why. So mobile phone, no thank you. But wearables, I'm too biassed to answer this as, like, what else there is than watches, wrist devices? But it could be maybe something in the ear, but also personally, my ears are very sensitive. I don't want to wear anything in my ears, or goggles, for goodness sake, no thank you. So what kind of wearable it can be, rather than anything else than the watch?

**Interviewer** 24:04

Yeah. So that's something we'll come on to later. What about after the exercise then, just carrying on with that. So, if you were to do the exercise and then afterwards you want to assess it, would you then consider a different device?

**Participant 8** 24:24

Yeah, I can consider different because in wearables, the screen size, if there is any, screen size is small or if there is no screen, so I can-. If there is some kind of analysis of my movements or the quality of my movements, I would rather check that later when I have time and the situation is good. Because usually either I'm in the gym, so I don't want to start analysing my movements there, or I'm running or skiing. So I don't want to be outside and you know, it's Finland, always cold. So I want to come home, you know, have my snack or lunch or whatever, eat a little bit, then I have private time or time to check what kind of exercise I actually did have. So it can be web or mobile, or whatever, wherever the screen size is big enough that I can analyse, read it easily. Probably check the analysis if there's pictures or graphs and curves to show me that the results, I can check them easily. So screen size, big enough. So not right after exercise. A little bit later, later in the evening, or next day.

**Interviewer** 25:58

So how much detail would you expect feedback to provide? And I know, again, you've kind of touched on this one a little bit.

**Participant 8** 26:09

Well, on what I'm doing, like my sports, I just want to know that distance and speed and pace, like the basic information. And that's not maybe the quality of the movement so much, but of course, that heart rate graph. So how did I-. How did my heart-. How heavy was the exercise? Or how did I achieve to stay in the heart rate zones, if I was doing a run or skiing and I have to, you know, I cannot go too high. So that's like the quality of my movements, what I'm checking currently. But sometimes if I'm like-. What I would like to have is, when I'm riding, horse riding, I would like to see that when we are trotting and when we are galloping, and so I can divide the exercise in different parts, but that's like somewhere else in the future, not [company] style.

**Interviewer** 27:12

So what I'm going to do now is I'm going to show you a couple of examples of visualisations that exist. Some of them consider movement quality, some consider movement quantity, but it's really just to put something on the screen that you can see and then interpret it and see what kind of features you like and what you don't. They're a bit mixed. Just going to try and share a document. So can you see that?

**Participant 8** 27:57

Yes.

**Interviewer** 27:57

So without me, prompting at all. Can you take a moment, have a look at that [Figure 1] and see if you can work out what you think it's trying to show.

**Participant 8** 28:14

It's some kind of graph about, I'm throwing something, maybe a frisbee or something that has a round movement, or my hand is doing, maybe, javelin? So, probably, the velocity is like metres per second. So it's the speed of the throw? But I didn't know the band that indicates the tolerable amount of error, I don't understand what that means. I don't know what that is about.

**Interviewer** 29:16

So I'll explain a little bit then. So, basically, the, as you said, the coloured line is the person throwing. In this context, they were throwing a ball. This is the path [coloured line] that the wrist followed. They wore a wearable on their wrist and it's the path that their wrist followed. The green line is the ideal throw, so it's what they're trying to achieve. And then this pink area, then, is a level of tolerance. So the idea would be if throw came outside of this band, then it will be considered a not good throw. When it's within it, it's considered to be a good throw. So that's the general idea with that. Is there anything you like about it? And is there anything that you dislike about it?

**Participant 8** 30:19

Well, it was quite easy to understand like, that it's, okay, because it's written there, that it's like, curve of my javelin throw, or throwing something. But I didn't understand the meaning of that purple [pink] area, or that indication. Now when you explained, yes, I got it, but from the visuals, I didn't get it. But I like the 3D style anyway, that's quite nice.

**Interviewer** 30:54

Okay. Is there anything in particular that you don't like about it that you would change or-?

**Participant 8** 31:01

Well, maybe I don't go to design like the gold standard versus the participants throw. They are visually too close to each other, so gold-standard should be presented in some other way than having this. So in visual, they are a little bit too similar, but it's not easy to, now this is the gold standard versus the participants throw. But otherwise I-.

**Interviewer** 31:39

So, if I move on to the next one, then this, one on the left [Figure 2]? This is-. Obviously it's an Apple Watch. Are you able to roughly work out what you think it's trying to show? And then we'll have a conversation about it.

**Participant 8** 32:00

Well, I was actually trying this Apple Watch before summer, and I couldn't find out what these curves were about. And I didn't want to find out from any kind of information source. So I'm not sure what they want to show me. But it's something that maybe the red one, the outer curve is about when I'm running or, you know, the high intensity level ofmy activity today. I don't know how the levels are defined. But anyway, that's like the high intensity part of my daily activity, then the green one is like the middle activity level, and the blue one is the resting level of my daily activity. But I don't know about the arrows. Like why there is this one in the red one is showing to the right and green is two arrows showing to the right, I don't know what they are about.

**Interviewer** 33:05

So before I tell you exactly what it shows, could you tell me why you think maybe the red is the high intensity, the green is the medium? What is it about it that makes you think that?

**Participant 8** 33:19

Oh, that's easy to answer because I'm biased by [company] colours, and we show those activity or the heart rate zones with these colours. So red one is high zones, and green is middle zones, and blue is our activity, like all activities. Our activity colour, so-. And also blue is for sleeping. You know, that kind of colour that is easy and relax and sleep or something. I'm a little bit [company] oriented here, so that's why. And red is, of course, colour that shows alertness and is something to do with high intensity or I'm alerted or you know-.

**Interviewer** 34:10

So the red one actually shows your total daily activity. So how much activity you do in a particular day. It's actually the green one that shows more intense activity. So intensity above a certain threshold. So that's why it's got the two arrows, because it's supposed to be more brisk movement. And the blue is time spent standing. So basically, every every hour it gives you-. You're supposed to stand like five minutes and as you hit those targets, it goes round. And then obviously the aim is to try and complete the ring throughout the day. What do you like about it? What do you dislike about it?

**Participant 8** 35:00

Well, I'm a little bit too professional to say that I didn't like that the red one is showing the whole day activity, because red as colour is indicating to me that it's too much and it's a danger and it's not-. It's a negative colour. Or it's showing me high intensity levels in my world. And I didn't get the arrows at all still. Blue, probably, this one's [the arrow] showing up, in the blue is like stand up maybe? I'm not sure. So I didn't like that so-. But what I liked is that they have been combined here. All their activity levels in one graph. I don't like the colours and it's not very self explanatory, because, I don't know, still I have to all the time analyse that "What was red? And what was green? And what was blue?", because there is nothing showing me what the colour is for. But what I liked is that they are having all in one, in one round or whatever circle this is.

**Interviewer** 36:26

So then moving on to the last one [Figure 3] here on the right, so this guy is doing a squatting movement. You might just be able to make out he's wearing a sensor on his leg, he's wearing one on his chest. He's wearing a couple of other ones as well, but you can't really see them in the picture. And then this is the app user interface that is then showing his movement and giving him feedback. So if you were to look at that. What do you think it's trying to show first of all?

**Participant 8** 37:09

Well, I think, that the circle is quite clear. At least what I maybe understand is that the red one is showing that you are bending forward too much, like, there is this black sign showing that where is your bending? What's your angle right now in the circle?. And that's over the good movement limit. And the limits are here on the-. They're good movement limits, if it's correctly said, I don't know, are here signed with the green colours. So you have to stay inside those green areas to have a decent or good movement. So if you are bending over it, you are bending too much. And if you are bending this way that, like, then it's definitely not a good movement probably for squatting or-. And then do you want me to analyse the graph?

**Interviewer** 38:22

Yes, please. Yeah. So you pretty much got it. So the maximum, mean, and the minimum is obviously from what he's done. So the maximum he's bent over up to this point is 43.92 degrees, the minimum is 38.5 [degrees], and then it gives his mean value. The red tolerance area will correlate with the circle here. So that is basically showing his previous repetitions, as you've said, and whether or not he was within the boundary. Obviously, he's in the middle of this repetition, so when he finishes this one, it will go down there, and it will probably tell him that he's gone outside of that limit to 64.18 degrees presumably. Yeah, the plane, I think, will vary depending on the view. So this is showing a side view, and then you can change, then, the angle that you're looking at. So this is looking at his spinal flexion, you can change it to show, like, his knee angle, and so on as well. So, again, same questions, what do you like about it and what do you dislike about it?

**Participant 8** 38:23

Okay, now it shows me that with the orange or this yellow, this is giving me the angle of my bending. I don't know, where do I need that information that it's 64% [degrees] probably, like the angle he's bent? I don't know, where do I need that? And I don't know what plane on the left side in the yellow or orange. But then spine flexion. So probably I've been doing four repetitions, four times bending, and it shows me that-. I don't know why there's three, two, and four and five [reps], because it's saying to me there is four reps, so it's not from the number one but-. That my maximum bending is 43% [degrees] and minimum is 38 [degrees] and I should be somewhere in between these red lines. Or is that my mean? I'm not sure. Should I stay between these red lines? Or is it just showing me the means. Maximum and minimum and the mean, you know, it's just calculating that this is that-. I think they circle, red and green circle, is very clearly showing me that you should stay, at least I understood, because the red one is a strong colour. So don't go to the red zone and stay in the green zone. I'm not sure what's the lighter greens and then the darker green there? Probably it's that the darker green shows me that means, I've been in this area. Like, you just said the word, anyway.

**Interviewer** 42:21

Tolerance?

**Participant 8** 42:22

Yeah, and then this range, like, lighter greens are the maximum range? I don't know. But anyway, I like that there is this easy, easily-. I can see that, now, this black one is showing me that I'm outside of the zone and I'm in the red zone and probably I shouldn't be there, because the green one is one that I should be. And then the graph in the bottom is okay. I'm not sure what I would do with that. But I like graphs and maths. So I would like to see some figures and curves and bars, so-. Probably, these would tell me why I need that information. Or like, why do I have to collect this information? So that's what I'm missing here a little bit.

**Interviewer** 43:25

So, having seen those visualisations, has it changed your thoughts at all on how you might use visualisations for feedback?

**Participant 8** 43:52

Well, maybe if I'm doing those exercise my physiotherapist has given me for my back, because sometimes I had to do that kind of therapy movements, and they are very exact and precise and-. That would actually help me a lot, because I just told you that I don't like to have carry my mobile phone and I don't specifically want to follow any training programme during, [when] I'm exercising, but in that case, I would use it because now it has been like that, that he's telling me these movements when I'm in the physiotherapist visiting him, then I go to the gym and like "There's, like, three moments I have to do" but still like, "Oh, what did he say?" and then maybe he gives me a piece of paper and I forget it at home and then I have it [the mobile phone] with me and, you know-. So that would help me a lot that it tells me while I'm moving that, "Too much bending".

**Interviewer** 45:02

So just to clarify on that one. So are you suggesting that you possibly would use a mobile phone? During exercise? If you're doing that type of movement where you're in the same place rather than going for a run, for example? So maybe whether or not you use a mobile phone in real time depends on the type of exercise you're doing?

**Participant 8** 45:29

Yes, it depends on the exercise type. Not while I'm running, I don't want to have it analysing my quality of my steps, running steps, because too much information, but in the gym, doing my physiotherapist exercises, yes. But I would also need those wearables to-. But that's probably the case that you are solving here is that, how can I wear that? What kind of wearables I could have on my, thighs and back?

**Interviewer** 46:08

So how do you think, then, the general population and the customer base would find the use of visualisations?

**Participant 8** 46:21

Maybe it's quite limited. So probably big audience, not so much. But these kind of specific sports like weight training, or physiotherapist exercise, where it's really-. Specifically, you have to check the quality of your movement is cleaner. So, I cannot answer your question, but it's just a guess.

**Interviewer** 47:03

So I think we've covered the next question. It's asking about the level of detail, and how easily they might be able to interpret the information they get. So I know you said earlier about keeping it simple is best during exercise because you don't want information overload. But do you have any additional thoughts on that? The level of detail, and how they might be able to interpret it?

**Participant 8** 47:35

Like what you showed, where that was the circle and the graph [Figure 3], the graph is too much when I'm doing the exercise, but that's just like my personal opinion. Right now, what did you saw? Like, what did I just saw, because when I'm doing the bending and the exercise, I don't want to have the graph and statistic telling me, I can maybe slide if you can switch the view, but I just want to see the the man inside the ball. Like, as simple as possible.

**Interviewer** 48:21

Yeah, yeah. Cool. How would you think that the users would use the information that they're provided?

**Participant 8** 48:35

During the exercise, or-?

**Interviewer** 48:37

At any time.

**Participant 8** 48:38

At any time? Well, in that case, like, my aching back, I would check that the movements are correct, and also counting the movements, I like that, that it tells me that four repetitions made [Figure 3]. So I have some statistics telling me that I've been doing this exercise and so many reps, so I can follow the progress. Not only during the exercise, but then after it has that statistics that I can follow. So because my back is aching, and I have to do this exercise for some time, and then I had to call the physiotherapist and I can show him to prove that I made my home lessons, you know. But anyway, following the progress.

**Interviewer** 49:32

Okay. Do you foresee any concerns that people might have about the information they get? So we have a feature, it gives them feedback about their movement. Do you think they'd have any concerns about having the information?

**Participant 8** 49:51

Well, maybe they have a little lack of trust. So is it valid enough? Because it's a machine. So is, it valid enough? Is it really true what I did and how is it interpreting it to me? So, is it correct?

**Interviewer** 50:12

So where do you think people would be willing to wear a monitor to capture their movement? So you said earlier, you pretty much just like the wristwatch. You don't want anything in your ears or on the goggles or things like that. But would you consider any other locations, and do you think other people would consider any other locations?

**Participant 8** 50:40

Well, if it's this kind of wearable, wear it in my hip or in my thigh, maybe. Because then it would show me the movement. I know that you cannot show the movement without the device or it cannot magically be showing my movements if I don't wear anything. So some kind of band. Nothing in the head. But I have been following like this future-. I have a couple of students on one course from the university, they are planning the future devices, these kind of tasks that they have to design and not implement, but designing future wearables, and they are always showing me that they have to wear an overall or something, and I know that's not going to happen in reality.

**Interviewer** 51:46

So I've got my own thoughts on that as well. But could you expand on that point a little bit? Why do you think they wouldn't want to wear a complete suit?

**Participant 8** 51:59

Because it's sweaty. It's hot and sweaty and you have to wash it afterwards. You know, it's these kinds of practical things that makes it-. You have to remember to take it with you, you have to you have to wear it to the locker room, and, you know, try to wear this tight suit, because it has to be tight, because it cannot control you if it's loose. And then it's hot, and it's sweaty, and maybe it's that kind of material because of the technical devices integrated, and so it's not very flexible, and it's not very thin. So that's like the case that the material is not light enough to be easily wear [worn]. So it has to be, like you said, [a] band. That it's easy to wear.

**Interviewer** 53:00

Yeah. So I'm saying this out loud, almost more for me to note as well. But I think that when you were a complete suit, as described, like, if you were to do a certain movement, the fabric might pull across a certain part. So say, for example, you open your arms up, the fabric might pull across and move the sensor because it's all interconnected. Whereas if you have just a band, you could do that [open the arms apart]. And it's not going to move on the arm relative to what it's doing in other places. So it's not all so interconnected. Do you see any potential limitations to using wearables to assess movement quality?

**Participant 8** 53:49

Well, maybe-. I'm not professional in material techniques, but the wearable has to be tight enough, but still allow the movements, so it's going to be tricky, so that's probably the problematic case. I probably didn't answer your question.

**Interviewer** 54:21

I think it is in line with what I'm trying to understand. What about ways that we can facilitate people using wearables. So ways we can encourage them to actually use it and say, "Yes, this is beneficial", and get them to actually wear them?

**Participant 8** 54:45

Yeah. So what was the question how we could-?

**Interviewer** 54:53

Encourage people to use them.

**Participant 8** 54:59

I don't know. To tell them that "If you wear this wearable, like a sleeve or trousers, or the band, you will get really good information about your movements that we really-. For your exercise or your health, it's good to wear these wearables to have your health data.

**Interviewer** 55:33

So it has to just be something good that they'll use to justify actually buying a wearable or wearing multiple sensors? Yeah. So what are your thoughts on introducing this in [company] products?

**Participant 8** 55:44

Yeah, so they have to be sure that wearing these, even though it's a little bit cumbersome to wear those, it's good for their health, or they will get so good data about the health situation or the movements or it's, "You have to do it, you have to wear it". So I'm motivated, not only because of the data itself, but I'm gaining something good from wearing this. Yeah, sure. My back is healing because I'm wearing this. What do you mean?

**Interviewer** 56:31

So, what would your thoughts be on introducing some sort of feature that would help people to move better in [company] products?

**Participant 8** 56:42

Yeah, I was thinking, did you mean like wearable, like these sleeves or bands and-. I think it would be great. We are always discussing it here in [company] that we are-. It's too much about just showing the raw data to the users. So this kind of qualitative data about my exercise during what I'm exercising, this is quite qualitative. So it would be really beneficial for the end user to see and know how they are moving and what's the quality of their exercise, not only after, showing the repetitions or their heart rate and their-. So it could have some-. Our data would have some-. It would be much richer, you know. Not only heart rate data, but something counting more in that [assessing movement quality].

**Interviewer** 57:56

Okay. Yeah. So that's something we're trying to look at is that it's not just about quantifying how much people do but like giving them something else to think about, because movement's only so good if you're also doing the movement correctly. So that's kind of trying to complete the picture really.

**Participant 8** 58:20

It would be nice to have some qualitative data during the exercise, not only quantitative will always giving me the heart rate and the speed etc. But nothing like-. It's not analysing my exercise, my movements, in any way. Just showing the raw data currently, so this would add some advantages on that.

**Interviewer** 58:51

So before we finish, do you have any final thoughts or comments or suggestions that might align with project?

**Participant 8** 59:03

Well, I hope that the wearable, because if you have to have this extra wearable, it would be easy to wear. So I don't know how else can you have that qualitative kind of data without anywhere extra wearables, so I hope that you can combine these two easily.

**Interviewer** 59:30

Yeah. I just going to ask one last question. Just because I want to ask you about working in your UX position. How would you actually expect some sort of visualisation to look? Have you got any ideas or experience in how you'd actually expect it to look on a screen?

**Participant 8** 1:00:03

Well, it's if it's a wrist device, it's very tricky to show it on the screen during the exercise or even after that exercise. So I do not have a good answer for that. I know that the screen size is so small that it's really, really a tricky case to show it on this device. So I wish you good luck. But in mobile or any other screen, like bigger screen, just like again saying that it has to be very easy. Very, very easy. Not like having both the visual and the graph [as per Figure 3]. You have to divide it to the very simple parts, because during the exercise, I don't want to check it in details. I just have to glance it, like, I have my whatever screen I have, then I like do this [demonstrates a quick look at a watch], and then I continue. So easy to access, very fast. Like, I'm going to watch it one second, maybe two, so-. The colours and the visuals has to be very simple.

**Interviewer** 1:01:13

Yeah. Cool. Okay, that's the end of the interview.

# **Participant 9 – Customer Experience**

Mon, 8/29 1:54PM • 35:20

**SPEAKERS**

Participant 9, Interviewer

**Interviewer** 00:07

Okay, so as discussed off the recording, this is a very informal conversation. There's no right and wrong answers, you don't have to answer any questions that you don't want to. Basically, we're just keen to get your thoughts and your perspectives. Everything is confidential and anonymous. So anything that you've said that would identify you is for my eyes only, and in anything that will be published or presented to others, your identity will be anonymised. So yeah, again, I'm the only person that will see that. Before I get started with the questions, I just want to get to know a little bit about you. So if you could just introduce yourself by telling me a little bit about your role at the company, and then also what you like to do in your spare time outside of the company.

**Participant 9** 01:03

Okay. So I'm [name omitted], and at [company], I'm a lead designer for the services [Customer Services], so I help plan the services and how they work, and what is the experience that the users get from it. So the business of the Customer Services, so [company application], in both web and the mobile, so that's kind of my area of action. So I end up dealing with the researchers and with the developers and with the users, we kind of try to bring everything together.

**Interviewer** 01:42

Okay. Is that through interviews and conversations like this? Or are you more into feature development?

**Participant 9** 01:51

It's both. It depends on what stage we are. So we conduct research interviews with users that we're trying to figure it out what they what they need, and it might be in a very generic level, in the way, the service as a whole, or we might do it tied with a feature as well if we have a goal of developing certain features. And in that way, we're usually cooperating within the research.

**Interviewer** 02:20

And then in your spare?

**Participant 9** 02:21

In my spare time. My spare time, yeah, I have two kids and a dog, so it kind of takes most of my spare time. I don't practice any kind of goal-oriented sport, but I do like to go to the gym maybe once or twice per week and during the winter I try to play some team sports with co-workers as well, so-. But nothing too serious, but keeping alive and healthy as much as I can.

**Interviewer** 03:00

How do you travel to work?

**Participant 9** 03:02

Usually by car.

**Interviewer** 03:05

So something we're going to talk about is 'movement quality'. So how do you how do you interpret the term 'movement quality'? Someone's speaking to you about 'movement quality'? What are they talking about?

**Participant 9** 03:21

So I would assume that it has to do, well, whether or not we are performing the movement in a way that it's not injuring you in any way, so doing the movements, right. Or if it's quality, perhaps like if we are doing it to a benefit of some sort. But that's as far as I would read into it.

**Interviewer** 03:51

So pretty much. Yeah, you've got it. It's all obviously about doing the movement correctly and proficiently to prevent injury, to increase performance, so on and so on. What sort of things do you think might help someone to move well, first of all, during everyday life? So, you're with your kids, you're doing your food shopping, you're walking the dog. What things might help you to move well, in everyday life?

**Participant 9** 04:25

Yeah, that's quite intriguing in a way. I think I have never considered quality of movement within everyday life. I do consider it in the in the gym context. There I have a conscious effort to do it right. Of course, not break myself, but in everyday life. I don't know actually.

**Interviewer** 04:50

What about in exercise?

**Participant 9** 04:53

In exercise, yeah, I tried to use the muscles appropriately, and that is the help I've been getting in a way, like, I have a trainer [personal trainer] with me to be able to do things in a proper way movement-wise. But in everyday life, I guess it's kind of simulating the way, like, if I'm lifting grocery bags that are heavy or something that I kind of approach it in a way-.

**Interviewer** 05:24

So it's the transfer from what you've maybe-.

**Participant 9** 05:26

From the exercise, yeah, exactly.

**Interviewer** 05:29

Okay, so subtly different question, but what do you think might influence how a person moves? So maybe external factors that could contribute to how well you move?

**Participant 9** 05:45

For me, in particular, the weather is an issue. For instance, I strain myself quite a lot if I'm working on ice. That's not a natural thing for me. So I have noticed that, I can notice that something is pushing me there. But I guess whether or not you can afford to be cautious about it, I guess. So if you're like in a rush or trying to get kids out in the morning, or something like that, you might not do anything wrong or right, in a way.

**Interviewer** 06:29

Okay. So what benefits do you think that people could have by improving how well they move during exercise?

**Participant 9** 06:40

Usually prevention, that would be one. And then of course, they can get the full benefit of the exercise, that you're doing what you're supposed to do.

**Interviewer** 06:49

Okay, so we're exploring ways to help people understand how they move, and how they can improve the way they move doing exercise. So currently, there are no affordable, accessible and effective ways in which this can be achieved. So to illustrate that point, if you have a personal trainer, for example, you have to pay them an ongoing hourly rate, and usually have to go to a facility to use that service. You may also have a physiotherapist if you're trying to work around an injury or work through an injury. Or you may even have a lab-based system, which could have expensive cameras, you need a lot of space for it, and it's not really practical to the everyday person to go to a lab and do their training session in that environment. It's more reserved for elite athletes. So with the use of wearable technology, we're hoping to change this. So we're looking at how we can use wearable technology to measure and assess movement quality, but also we're looking at ways that we can make the data we collect easy for people to interpret, such that they can then implement it safely and effectively. So are you able to give me some examples of feedback methods that you're aware of that could help inform people of their activity? First of all, how much activity they do. So can you think of any examples where people get information about how much activity they do?

**Participant 9** 08:36

Yeah, I guess all these training load measurements like we [company] have, and general activity quantity measurement. So you can get, basically, mostly from heart rate and gyroscope based ones. I think that's probably the most common about how much activity. I wonder, anything else that I can remember? Probably not.

**Interviewer** 09:14

So, then, what about measuring how they move?

**Participant 9** 09:21

Yeah, quality that-. So measuring not instructing?

**Interviewer** 09:28

Getting feedback. So forget about the method by which that feedback is delivered. Think in a broader sense as well.

**Participant 9** 09:39

Yeah. Because, yeah, I have seen services that provide, for instance, like video instruction for an exercise, that they will focus very carefully in the movements, but I think the feedback is not that direct. So it's just an instruction in a way. So getting shown is the way to know. And for personal use, I don't know if there is any. I can't to remember. Well, maybe some foot pads that has the right-left balance, and things like that.

**Interviewer** 10:22

So something we've been really drawn to is the use of visual aids. So how do you understand that? What do you think I mean by 'visual aid'?

**Participant 9** 10:32

I guess it could be several ways. Could be, like an augmented reality type of thing. It could be, like, afterwards, if you managed to get your exercise recorded personally, and analysed that way. Or, I guess, if you would have distributed sensors, you could [unintelligible], like, are they ideal-. Like, the positioning the right way, so signal produced-.

**Interviewer** 11:14

So effectively, what we're looking at is some sort of way of delivering some visual feedback to the user through the use of imagery, or video or animation, something along those lines. So something they see. What do you think people would think of something like that?

**Participant 9** 11:38

Would they be related to, or really connected to what the user is doing?

**Interviewer** 11:45

Yes.

**Participant 9** 11:45

Yeah, I think that would work. Yeah, I think that would be interesting. I think people are used to having that kind of imagery, without the feedback. So I think if it's with proper feedback, would be yes.

**Interviewer** 12:00

So would you like something like that?

**Participant 9** 12:04

Yeah, I think it's good helped me.

**Interviewer** 12:07

Why?

**Participant 9** 12:09

And again, because I don't know if I'm doing it wrong. So it could be helpful if it tells me and shows me how.

**Interviewer** 12:20

Can you think of any examples of visualisations that help people during exercise?

**Participant 9** 12:29

Yeah, there's, for instance, and it's probably easier to think in terms of strength training, but they have these visualisations that show you like in a certain movement, what kind of muscles you are activating. Something like that. Those are interesting as well. So I guess it would be interesting to know if you are doing the wrong movement. I have not seen it this much outside of the labs for, like, running technique and things like that. But it could be interesting.

**Interviewer** 13:03

So if we were going to provide visual feedback, so some sort of imagery or video, however that might look, do you think that would be best delivered in isolation? So you just get visual feedback. Or do you think it would be best if it was accompanied with something else, for example, text or audio or vibration or something else?

**Participant 9** 13:29

I think that, usually, text and video are a good solution. It brings up different aspects. If it's tied with my movement directly, so like a feedback towards exactly my movement, then that connection, if that connection is strengthened, it might be useful either by the vibration or visual cues or any way that you kind of understand that those do match.

**Interviewer** 14:07

Okay, so if we're giving feedback to people on their movement, what sort of devices do you think would be best for that?

**Participant 9** 14:16

I think probably the phone is one, that it's always there, and you have a nice size screen and the capabilities to get with it. In the wrist [smartwatch], potentially, but I guess it depends a little bit on how detailed this feedback would be.

**Interviewer** 14:42

Yeah, so that's actually my next question. So how much detail would you actually expect the feedback to provide?

**Participant 9** 14:53

I would probably go for more detail rather than less. But I think it's a journey in time. So If I would get better detail at the beginning, I guess I would be able to live with less detail as I learn how the feedback is structured and what should I do. So in that way, it might be a little bit-. Kind of different stages as well.

**Interviewer** 15:17

So what I'm going to do now is I'm going to show you a few examples of visualisations that exist, either in products or as a concept. Generally, visualisations are used to quantify movement, typically. So, if you think of [company] devices, there's the activity ring on the watch, the heart rate graphs on the phone, for example, with the [company] app and that sort of thing. I've tried to use a mix here. So there's three [figures], one of which shows-. But I'm not going to tell you actually too much. But first of all, this is the first one, so this is Figure 1. Just have a look at it for a second and see if you think you can work out roughly what it's trying to show, and then we'll have a conversation about it.

**Participant 9** 16:23

Okay, so this seems to be-. It is a throw, so is it a baseball, cricket, throw, something like that? It seems to be tracking a swing movement of some sort. And so it does kind of-. Yeah, he does the gold standard curve and then apparently how well they match. That sort of deviation,if you're further away [the throw trace compared to the gold-standard], it's kind of red. And then it-.

**Interviewer** 17:02

Yeah, so you're right, the green is basically the gold standard that you're trying to achieve. This is somebody throwing a ball, and the curve is the trajectory that a wrist-worn device followed. So that was their throwing hand. The coloured line shows the actual throw that they did and the colours show the speed at which it was moving at that time. So that's what that graph is [Velocity bar to the side of the main Figure]. So if it's blue, it's slower and then if it's red, it's faster. And then this, this pink section then just gives a tolerance as to-. So basically, when you're inside the tolerance, you're fine. If you deviate outside, then something's wrong. What do you like about it? And what do you dislike about it?

**Participant 9** 17:55

I mean, at the end, with the text help, you're able to understand it. And I wouldn't be sure how to translate these in any other way, but what I dislike is this-. I don't know. Maybe very technical.

**Interviewer** 18:28

Yeah. So that's a negative?

**Participant 9** 18:31

It's a negative and a positive, I guess. I guess if, I don't know, if you used to this kind of representation in the sport, it should be okay. But if this is a new representation, like if people are used to seeing-. If you're watching Formula One on the television, and there is always a lot of graphs, if you can translate those quite easily, but-.

**Interviewer** 19:04

It's probably something, then, that you think needs that bit of familiarisation first?

**Participant 9** 19:09

And maybe if it would be-. If the movement-. If it would come with movement, so it will be like a video-based graphic, it might be more understandable for a new user.

**Interviewer** 19:24

So rather than a static thing, it would actually show-.

**Participant 9** 19:26

Exactly, like you could see the turn [rotates arm to demonstrate rotation].

**Interviewer** 19:32

Okay. Moving on to the next one, then, this is Figure 2. So this is an Apple Watch. Again, what do you think it's trying to show, first of all?

**Participant 9** 19:44

Yeah, I do remember that this had something to do with the activity and sleep and training perhaps. And it's basically, it should be like, complete ratio, 100 percent curve or, I don't know if it goes to more than 100%. But they seem to fill up the circles. And how I would read it would be that, of course, if you you're balanced, then the circles would be filled in, like in a constant way. Like the three bars [rings], would fill at the same time. But if you are more towards one thing or the other, it might not. So I guess the purpose is to show you a balanced lifestyle.

**Interviewer** 20:35

So yeah, so basically, the outer ring shows you your total daily activity, the middle ring shows activity above a certain intensity threshold, so that was more like brisk activity or running or something. The blue is time spent standing. So it's a reminder that every so often you have to stand because you-, otherwise you're sat down all the time. And then as you've suggested, then the rings are supposed to go to 100 percent completed. What do you like about it? What do you dislike about it?

**Participant 9** 21:16

It's simple in a way that the completion part is a simple idea and you get it. So I like it. I didn't know what those icons [arrows] meant. Now that I know, they make a little bit more sense. So there's something of a learning aspect to it as well. But I think it's simple, so that's good. It's kind of a clear thing.

**Interviewer** 21:57

If I move on to the last one, then. So this is Figure 3. So this guy is just on here to represent what's going on. So he's wearing a couple of sensors. You can see there's one on his leg, there's one on his chest, he's wearing a couple of other ones as well, but you can't really make them out on this figure. And then this is the user interface of the app. So obviously, as he's wearing the sensors, that information feeds into the app. Looking at this app, again, what's it trying to show? Do you reckon you can work it out?

**Participant 9** 22:37

Okay. So it understands that he's having the back straight and the position, but it seems to be a little bit-. I guess, I suppose that it should be in the greeny, I guess. So it's a little bit too low. So you have some sort of a goal. I don't know if this is kind of in movement or not,-.

**Interviewer** 23:17

Yes, so this is basically the movement he is doing [the avatar on Figure 3]. What about the graph at the bottom?

**Participant 9** 23:35

Okay, so is it the angle of the flex of the spine? So I wonder, actually, what he needs to do? Does he need to flex within the green [green zone of Figure 3 circle].

**Interviewer** 23:56

Yeah, so I'll break it down for you then, and we can discuss it. So as you suggested, this black marker, ideally, would stay within this green zone. So it's to do with the angle he's bending over while he's doing the squat. So if he bends too far forward, he will then get a notification to say "You're bending forward". It would be very difficult to do, but if he was too far the other way-.

**Participant 9** 24:00

Then it would be a very awkward squat.

**Interviewer** 24:16

This just shows the previous repetitions. So this is obviously the current rep, so it hasn't gone down there yet. But it shows on the previous repetitions the angles that he got to, and then this red line represents the limits on the ring as well. So it shows that the last few are fine. This one probably would end up outside the red limit [on the graph] because he's gone that far forward. And then it tells him the maximum angle that he got to, the minimum, and the mean as well. And then you also have the ability to change the measurement that you're looking at. So this one showing his spine and it's showing a side view, so you could maybe change it to, I don't know, show the knee angle or something like that instead. Again, same questions. What do you like about it? What you dislike about it? Well, it seems to work as intended, I guess. I don't know if I would be analysing to this depth what I do, but-. Is that because you think it's too much?

**Participant 9** 25:55

I don't know. Usually, I'm just going for it quickly, and, you know, get it done and out [of the gym]. So in a way, I would probably not analyse that thoroughly what I'm doing. But on the other hand, if you're getting the feedback in a way that could be the first level of iteration that I would go for. That, am I doing it wrong, or I'm kind of bending forward? So I would probably pay more attention in that part. And then, of course, if I have made everything wrong, it's another thing, but I would not probably go into that much detail with the actual angles or anything if they would be presented.

**Interviewer** 27:09

Right, so having seen those, has that changed your views at all on, or your initial thoughts on using visualisations for feedback?

**Participant 9** 27:23

I still think that it would be useful.

**Interviewer** 27:27

Okay. How do you think that the general population or the customer base would find the use of visualisations?

**Participant 9** 27:39

That I wonder if, would you need to consider yourself kind of serious in your exercise in order to kind of get that extra step of analysing? Or the analysis can be that layman-. That you would learn the deeper benefit without needing to go with the deeper analysis, so that there is where I have kind of-. If I think how I would do it with a coach in a way, she would just telling me like, "Hey, you, you're doing it wrong. Can you adjust this and there?", but I would not be cautiously analysing the whole thing. So from that perspective-.

**Interviewer** 28:40

Have you got any thoughts around the level of detail that the general population might want, and also their ability to interpret the data?

**Participant 9** 28:50

I think the most important thing would be how you doing good or doing wrong, and whether or not that would require the graphical representation, I don't know. And then they still have "What should I do if I'm doing it wrong?". In that way, then you would need it, you would need to have like the writing structures. And I think that worked well, because you had the the red ball.

**Interviewer** 29:21

Sorry, that's Figure 3, the third one I showed you?

**Participant 9** 29:24

Yes, exactly. So the app, the quality of movement app. So in that sense, it would work well if you don't have any other way or to kind of get the information out. How should I do it right? And I think you even-. Yeah, I think a general person would, if you get advice that "Hey, you're not doing it right". And you don't [unintelligible], you would analyse it and try to figure out like, "What should I do to do it right?". So yeah, in that sense, yes.

**Interviewer** 29:56

So how do you think that the general population would respond to move on quality feedback? So, do you think that they would like to receive the information? And how do you think they would actually use the information?

**Participant 9** 30:19

I think they would. I think it's a common fear in a way that you are doing this movement wrong and you might injure yourself or-. So I think if you have it in an easy way, I think it would be welcome.

**Interviewer** 30:41

Okay. So do you foresee any concerns that people might have about the information that they're provided?

**Participant 9** 30:54

What information, no, I don't think so. Might be more like whether or not it's handy enough to can you where the whole apparatus? And things like that. But the information I think people will be happy with it.

**Interviewer** 31:12

Okay. Where do you think people will be willing to wear a monitor or a sensor to capture their movement? So, for example, the wrist is an obvious one.

**Participant 9** 31:24

Yeah, wrists are easy. I guess, legs or ankles could be possible as well. And torso, usually, people have also sensors in torso.

**Interviewer** 31:41

Where?

**Participant 9** 31:43

Under the abdomen. Heads could have potential, but I think it's a little bit more dodgy.

**Interviewer** 31:51

Yeah. Do you see any potential barriers to using wearables? So any limitations that might stop someone using wearables?

**Participant 9** 32:04

Well, price might be one. If you have like a lot of them [sensors]. Discomfort in general, if they are heavier, or clunkier, or if they require quite a lot of time to strap on. Or maintenance, if you are getting sweaty. And then in some sports, there might be barriers. Like if you have contact sports, it's a problem. Or as it happens with the wrist, even with our watches, if you're lifting weights in certain positions, they are a bit uncomfortable to wear.

**Interviewer** 32:57

So can you think of any ways that we could facilitate the use of wearables? So encourage people to use them?

**Participant 9** 33:08

If they are small enough, and they don't require-. So if they allow some tolerance of where they are, in a way, so that we can push them a little bit up or down the the arms or the legs. If they're very light and thin, it could be okay, I guess. There is always people that would be willing to get like a more uncomfortable or-, for the benefit, in a way. But if you want to grab as much people, like I said, it should be pretty much almost invisible and unnoticeable, I guess.

**Interviewer** 34:01

Okay. What are your thoughts on introducing something like this into [company] products?

**Participant 9** 34:11

I mean, we have already the one wrist. So I think that would be interesting things to do if we would have anymore-, two or three locations [for sensor placement].

**Interviewer** 34:23

What about a visualisation of movement feedback method just in general?

**Participant 9** 34:33

Yeah, I think that couldn't be fitting for us quite a lot. So, yeah, obviously, we have interesting use cases for that. Not only in the strength training side, but also the running technique or cycling techniques and things like that. So it would be interesting to have that.

**Interviewer** 34:56

Okay, so finally, do you have any other thoughts or comments on this project at all? It's interesting. Yeah. And I hope we can play with it.

# **Participant 10 – Product Management**

Fri, 9/2 2:33PM • 43:10

**SPEAKERS**

Participant 10, Interviewer

**Interviewer** 00:10

Okay, so, again, now that we're recording, there's no right and wrong answers. We're just really keen to get your perspectives and your thoughts. Everything is confidential and anonymous. And just to sort of give a brief introduction to what we're doing, we're looking at the use of wearable technology, and how we can use wearable technology to measure and assess movement quality. So before I get started with the questions, if possible, could you just tell me a little bit about your role at the company, and then also what you like to do outside of the company in your spare time?

**Participant 10** 00:52

I've worked at [company] for 11 and a half years, so quite long already, always in the product management. I work currently in the business Product Management team and under my responsibility, we have the sensor products and b2b products, and I also deal with a lot of smart coaching stuff. So that's me in a nutshell, what I do here at [company], I represent the business perspective on what are we going to build and when? Where do we aim towards? And that kind of stuff? Then me in the spare time, that was your second question, right?

**Interviewer** 01:37

Yes.

**Participant 10** 01:39

I used to be a volleyball player. But that was years back before kids and all that. In this second life that I have now, with the kids. I love sports. But it's not that I would do them as a primary thing anymore, but more to take my brain away. So normal family life, plus some cross country skiing, Les Mills exercises, and a bit of jogging and a bit of everything that I find a chance to do in terms of sports, and then I listen to a lot of audiobooks as well.

**Interviewer** 02:21

Okay. Cool. So, something we're going to speak about is movement quality. So, before I tell you what that means, how would you interpret the term 'movement quality' ?

**Participant 10** 02:38

Movement quality? You mean what do I think it is?

**Interviewer** 02:42

Yeah.

**Participant 10** 02:45

Movement quality, brings things like your technique, how efficient is your technique? That's probably the first thing that comes into my mind. And then secondly, I started thinking about the overall how important it is for your mind and body to keep yourself moving and that sort of a thing, like the quality of life that comes through the movement. Might not be at all, kind of-.

**Interviewer** 03:19

Yeah, it's, as I say, there's no right or wrong answers. It's just your interpretation. So when we're thinking about trying to move well, what sort of things do you think help people to move well first of all, in everyday life? So you're coming to work, you're at home with the kids. What might help you to move well, just in everyday life?

**Participant 10** 03:50

Well, I think there are two aspects to that. 'Move well', I think, in the first phase, it means that you are simply moving, you know, you keep yourself moving. And then secondly is how you do the movement that you do in the first phase. I think it's really important for people to find something they like, so by this I mean that if you try and force yourself to go running but you hate it, it's not something you will continue to do long term. So, finding something that brings joy and delight in your life, that's the first thing. And then kind of the 'move well', in terms of the forming the stuff right. That's the other aspect of moving well, but there, for me, that means coaching, instructor or somebody making sure you have learned the techniques in a right way. So you can then continue doing it in a right way.

**Interviewer** 05:09

Okay. So, you touched on it a little bit then, but this is subtly different question. What do you think influences how people move? So possible external factors that might influence how people move? I think you kind of alluded to that one anyway, so I might be answering my own question on this one, because you spoke about movement quality in terms of instructions. So you have that external coach or somebody in how well you move. But then, in everyday life, you also have-. You said about just making sure you are active, that then contributes to how well you move. So if you're inactive, it'll probably impact your movement quality as well. I don't know, do you have any other extensions of that?

**Participant 10** 05:42

For me, it's really natural to think through, you know, I've learned some things, I have participated in something where people have told me how to do things. And then, on my own, I'm then able to repeat it. In volleyball, you have a coach, in group exercise you have the teacher there. And that's kind of how you learn to do the things. And then you can do them on your own as well, like I do. So the coach aspect is pretty natural. But then again, of course, the performance of the day, and the quality of your movement depends on many things. Let's take an example. I'm planning to do some BodyPump today after work, but I have a headache. So then my technique might not be perfect because of that, because of the conditions that I am in that very moment.

**Interviewer** 07:10

Yeah, I suppose then it's looking at illness or stress, or those sorts of things that, obviously, then would influence how someone was moving. And I think, again, we've touched on this a little bit, but what benefits do you think people could have by improving how well, they move doing exercise?

**Participant 10** 07:34

How well? Well, it becomes more pleasant. Like, it feels better. It's more efficient. You're not wasting your energy, but instead, you're getting better results and faster. I'm thinking about myself cross-country skiing, when speaking. So the more efficient, the better the quality of your movement is, the better results you will get, the better it feels. Me cross-country skiing is another thing than me running. Cross-country skiing I've done for years, I know what I'm doing. Me running, it's a pain, it's always a constant pain, because I don't have the proper technique, probably, I don't have the efficency, I don't have the routine solved.

**Interviewer** 08:31

So we're exploring ways to help people understand how they move, and how they can improve the way that they move during exercise. Currently, there's no affordable, accessible and effective ways in which this can be achieved. And what I mean by that is that when we're speaking about affordability, perhaps if you have a coach, you have to pay a retaining fee, it can mount up to being quite expensive. Accessibility as well, with that one, you have to go to a facility normally to get that. So it's not just something readily available. And maybe also, you'll see the lab systems where they have cameras, and they're expensive, and they're reserved for those elite athletes then, so it's not really practical to go to a lab to have your movement analysed every time you exercise. Nor is it cost effective either. They cost £1000s. So what we're looking to do is use wearable technology, and hopefully change this. And we're also looking at ways that we can take the data and make it easy for people to interpret so that then they can implement it safely and effectively when trying to improve how well they move. So first of all, can you give me some examples of feedback methods that you're aware of to help inform people of their activity, first of all, for how much activity they do?

**Participant 10** 10:06

Well, our [company] watches, the whole service that we offer. Mobile phones have the accelerometer. And that will help and provide you with some information. And there's the whole world and your phone is full of information if you're seeking for it, I guess. And then of course, you can also track it yourself. Use a diary to measure it. Stuff like that.

**Interviewer** 10:32

So you said about, like the mobile phone and the [company] devices. Are there any specific features in mind that you could call upon?

**Participant 10** 10:43

Like, that measure how much you are moving? That's your question? Well, the daily activity percentage, for example, then we can record your trainings, as sessions, you have all of them in in your [electronic] diary. My personal goal, for example, at the moment is to collect those trainings in my calendar. I don't care so much about, you know, what was it? As long as I have it in the calendar. And it felt nice.

**Interviewer** 11:12

Cool. And then the next question, feedback methods for measuring how people move? So this is coming to the movement quality side of things. Can you think of any examples where people might get feedback on how well they move?

**Participant 10** 11:29

And now we're talking about the measurement side of things. Because the first thing that comes into my mind, but this is not tech really, is to participate in group exercise and the instructor tells you how well you're doing in a sense. But now moving this into the wearable measurement context, I know that there are systems that provide you that feedback. Stryd, for example, the foot pod that you attach to your shoe gives that kind of quality information on your running technique. I could assume there's something similar for the cyclists, as well. And perhaps also for some other sports. Also [company] the running power, for example, is a feature that you can use to have that sort of interpretation. So those are the first things I can think of.

**Interviewer** 12:37

So something we've been really drawn to is the use of visual aids. So first of all, how would you interpret that? What do you think I'm thinking of when I'm talking about visual aids

**Participant 10** 12:52

I see a picture of me in a digital format, running, and showing what I do wrong. Kind of having me visualised, and then showing that your stride length is too long, for example, or you are moving, you are using your left leg more than the right one, stuff like that.

**Interviewer** 13:27

What do you think that people would think of that as a feature?

**Participant 10** 13:33

That what I just described?

**Interviewer** 13:34

Well, any sort of visual feedback really, on how they move?

**Participant 10** 13:38

I think me and my jogging or running, attempt to run, is a good example. I wouldn't want to ruin my jogging session by all the time looking at all the errors that I'm doing. I just still want to have the 'go with the flow' feeling. But then afterwards, or then perhaps before starting [the next session], it would be amazing if somebody would remind me on the technique. Of course, to be able to do that, you would still need to measure throughout the workout, but giving feedback either afterwards, or before the next session, that would be the ideal for me. And then I think I'm not the only one. You asked more generally what would people think and I'd believe there is a demand for that.

**Interviewer** 14:44

Okay. Specifically, feedback after the exercise?

**Participant 10** 14:49

That's how I see this part. Of course, depends on on the activity that you do. Another example, I don't know if this would be possible in theory, but lets imagine another scenario. I would be playing volleyball and my coach is saying that I'm always late with my jump and my hand is not in an appropriate position perhaps, then a more live type of tracking on, you know, I do one jump, and then we see what I did and I do the next one. So, it really depends the occasion as well.

**Interviewer** 15:32

Okay, so I'm moving forward ahead of the shedule a little bit, but while you raised it, I want to dig into this a little bit more. Why are you leaning towards feedback after the event, rather than during it?

**Participant 10** 15:56

It will depend on, obviously, how is the information available, because now I noticed that I'm thinking about an app and having to watch the app to get the feedback. And then I'm thinking of, I don't want to fall or trip to anything that's there while I'm running. Or neither I want to ruin the moment of not having to look at any screens by having to have the screen there. So that's why I'm saying that I would like to take a look at it after and not during.

**Interviewer** 16:32

Yeah. That's quite interesting, actually. Because I think what I'm taking out of that, and please correct me if I'm wrong, is that when you're actually doing the exercise, and there's a pleasure element to that, that perhaps then you don't want the constant feedback to distract you from that. There's obviously more to it than that. But potentially, that's a factor. It's like, "Let me do my thing now and then let's worry about what I did later on, and I'll just try and keep that in mind for next time. But there in the moment, just let me do my thing."

**Participant 10** 17:01

Yes. Yeah, yeah, you're reading correctly. And I think this is why it's in my feedback so heavily, I think it also has to do with the fact that I work in [company]. I think about the measurement stuff and this technology angle the whole other time that I'm awake. So I really try and-. It's my time to give my brain a rest. And I love to see the results afterwards, ut only after. I would silence any beeping or anything. I do look at the heart rate, though. But it's only for my information. I go with the flow nowadays. And I do realise that this is a bit contradictory to what we're trying to tell people, but that's, you know-.

**Interviewer** 18:17

But then I suppose, this is the difference between elite level and potentially the more general person where this is actually probably more targeted towards. So talking about the visualisations again, and, again, building on what we just spoke about, do you think that feedback will be best given in isolation? So you just have some visual image, some animation, a video, something like that. Or do you think it would be best to combine that with things like audio cues, or text, like a sentence or some words, or vibration, or something?

**Participant 10** 19:18

Hard question, in a sense, because I could see the other elements like the audio or vibration or stuff like that to be used during [the exercise] to avoid the need to look at the screen. But then simply, the animation seems like a really efficient way to deliver a lot of information instead of having to read stuff. Yeah, animation together with some efficent graphics that would, with the time span, the focus span that we tend to have nowadays, I think that's simply the most efficient way to deliver information to a person. I imagine me after my jog looking at the movement quality reports, taking a look at three, five second animation on "This is what you did right and this is what you did wrong", would seem really efficient compared to having to start reading text.

**Interviewer** 20:30

So what sort of devices do you think would be best to provide feedback? I know you've said about your phone, but-.

**Participant 10** 20:40

What kind of devices? I didn't even really think about any other devices than phone. That's my first reaction to this one. But of course, we could have the watches that could provide you something. And of course, whatever sensors you would be holding, those could have a display as well. But the phone is the most natural place that comes into my mind.

**Interviewer** 21:11

Okay, so how much detail would you expect the feedback to provide?

**Participant 10** 21:17

From the quality of movement? Not too much, but it's because I'm not an athlete. Basically, I would want to know what I'm not doing correctly, spot the things, and then give me some guidance on, you know, "Use more of your right leg", or "Take shorter steps" or whatever would the feedback be. So not really detailed, and only like a couple of things that you can really try and correct instead of having too much.

**Interviewer** 22:01

So what I'm going to do now is I'm going to show you a couple of examples of visualisations. They're all different, so it's just get your thoughts on them. So, some of them are about moving quality, there's some that are more quantity. So it's more just to gauge your interpretation of them. So the first one is this one, this is Figure 1. You just have a look at that for a moment, see if you think you can work out what it's trying to show, and then we'll discuss it a little bit more.

**Participant 10** 22:49

Okay, so there is the correct movement [points to green line]. And then there is the movement from the person who is trying to perform the movement [points to multicoloured line]. And then you can compare yours against the one that is the correct and try and learn from it.

**Interviewer** 23:10

So yeah, essentially. So the movement, the coloured line, it also tells you the speed during the movement that you did. And this is somebody throwing the ball. And this is the trace that the wrist followed. There was a device on the wrist, so that's the pattern that the wrist follow. This pink area basically just gives a tolerance. So if you were to come outside it, it would be no good. If you're inside, it's good. And then the thing you're aiming for, then, is that green line. So that's the idea. What do you like about it? And what do you dislike about it?

**Participant 10** 23:54

The tolerance area, it's a bit hard to understand, I think, and the fact that things go on top of each other [the two lines cross], make it hard to really know where I am at. I would say then, on the other hand, now that you tell me what the movement is, that you're throwing something, I can imagine that this is exactly how the movement goes. That is the good thing. It's really easy to see from the green and kind of the red lines there and figure out what is the part of the movement that, you know-. But then somehow the tolerance stuff is just really hard to understand.

**Interviewer** 24:50

Okay. Moving on to the next one [Figure 2]. So obviously, this is an Apple Watch. Again, if you just look at that, see if you can work out what it's trying to show.

**Participant 10** 25:05

This is a good test, because I am not familiar with how Apple interprets things. I probably should be, but I'm not. But I could guess. This is my guess, that similarly [to company products], then, we have the heart rate zones, where your 50-60 [%], 60-70 [%], and all the intensities, the intensity of the activity is in different categories. I can imagine that this is something similar [to company products]. So this is, is this now the other way around? Then we have this activity would include a lot of the high intensity [points to red ring], and then less of the lower intensity [points to green ring], and then only a little of the low intensity [points to blue ring]. I don't know.

**Interviewer** 25:11

That's why I chose it [laughter]. Okay, so the outside curve is actually your total daily activity. It's how much you've done in a day. The green line is how much activity you've done above a certain [intensity] threshold. So that's actually your more intense activity. And then this blue one is standing time. So you're supposed to spend a certain amount of time standing each hour, and basically, if you stand up enough time each hour, that accumulates, and then of course, the idea is to complete the ring. So what do you like about it? What do you dislike about it?

**Participant 10** 26:51

Well, I don't like the colours, but it's maybe me and the [company] aspect again here, because, for me, red means either high intensity, or you're doing things incorrect. So red colour, I wouldn't use it. If this would have been like blue, or something, green, or something, that would have-, I wouldn't have misinterpreted. So that's the first thing. What I do like about it is that it has a goal. Try and fulfil the circle, that would be my goal. So that is-, it gives me the purpose for things. That is what I get from the shape. The circle shape.

**Interviewer** 27:41

Cool. And then finally, this is the last one [Figure 3]. And now this individual here, is doing some squats. And you can see he's wearing some sensors. So there's a sensor on his leg, there's one on his chest as well, he is wearing a couple more, but you can't actually see them clearly in the photo. So it's not just the two, there's a few on there. And then this is the app user interface then, which is telling him about his movement. So again, without telling you too much, see if you can interpret what that is saying.

**Participant 10** 28:21

Interesting. "You're bending forward". So for me, that would seem like "Don't do it". You're bending forward too much. And there's quite a lot of red here as well. So, to me, it seems that the movement is not perhaps as good as it could be. Then, what are these here? Some sorta degrees? Or can it be how, to what decrease you're squatting? I don't know. And these would be the repetitions then. I don't know.

**Interviewer** 28:22

I'll break it down for you, it's okay. So, specifically here, this is showing his back angle. So when he's squatting, it's him bending over. This black marker [on the circle in the top portion of the app user interface], the idea is this should stay within this green zone.

**Participant 10** 29:21

Okay, so he's not doing it correct?

**Interviewer** 29:23

Yeah, so he's gone outside [the tolerable range]. And he's gone too far that way [too far forward], in which case, you get the feedback that "You're bending forward". I'm guessing if you were there [too far the other way], you'd be leaning back, but actually you'd probably just fall over. This at the bottom [graph at the bottom of the screen] shows the data that's in the circle, but for the previous repetitions. So these red lines align with these [end ranges of the green section of the circle], and then that shows, the bar then shows, the back angle that he got to from the previous repetition. And then gives the minimums, the mean, and the maximum.

**Participant 10** 29:59

Yeah, this side [the graph at the bottom] I had no clue. Here I wasn't-, yeah.

**Interviewer** 30:04

So, the other thing you can do as well in this particular app is you can change the view, and you can also change what's being measured. So this is measuring his back angle, I guess you could change it to be, like, knee angle or something, and then you can change the view from the front or the side or wherever. Again, same questions. What do you like about it? What do you dislike about it?

**Participant 10** 30:25

Well, I'd have to say, according to what I did understand and what I didn't like, the picture here [top half of Figure 3], and especially the text is spot on. It's really easy to get what I'm doing wrong. And because of the text, especially. And then this part [bottom half of Figure 3] is just not easy to get. Without your explanation, I don't know. I wouldn't have understood it. If I had the app here, I could follow it while doing, and then I could see the bars filling up here more, then perhaps.

**Interviewer** 31:07

Yeah. So we like the simplicity of the top section, but then perhaps there's when you get into the statistical analysis of the bottom, it's a bit too much?

**Participant 10** 31:18

Yeah. And all the degrees here, it's too much. But then, again, this figure [animated avatar on Figure 3] here and the circle, and personally, the text, that's clear.

**Interviewer** 31:27

Cool. So having seen those, has that changed your perception at all of using visualisations for feedback?

**Participant 10** 31:40

Yeah, yeah. Well, the simple text was great, unlike I first thought that it would not be needed, but there in that [Figure 3] it was really kinda, without the text, there would have been nothing really. Then, still, the less numbers, basically, the more interpretation that you need to do on top of the numbers, the better it would be.

**Interviewer** 32:12

Yeah, cool. So basically, we just want the minimum effective dose. It's keep it as simple as we can to get the message across, nothing more.

**Participant 10** 32:27

Yeah, for the guidance part. Then, of course, I know that, the more into that you are, the more athlete type of a person you are, the more you want to dig into the details as well. But just for the normal people, the simpler, the better.

**Interviewer** 32:45

So do you think, then, if I use Figure 3, that last one as an example, potentially, then you have the top part where the avatar is doing the movement as the real time feedback, and then the data might not be useless, it might be something useful for later but potentially not on the same screen that you can just call on when you want it. Not, like, there's too much on the screen at once.

**Participant 10** 33:17

Yeah, the top part could definitely work.

**Interviewer** 33:23

How would you think that the general population would find the use of visualisations? And I know, we've spoken about this one a bit before. And also, what sort of level of detail do you think they'll be able to, or level of detail they would want, and how they'll interpret it.

**Participant 10** 33:41

General people, I'm immediately thinking of my Mum and Dad, type of people. They do quite a lot of cross-country skiing as well. And I'm talking about them because they might represent the 'not techie' people. The most challenging people to get the message across. But still, if this would be a part of an app that they're using, to, let's say, track the kilometres that they are skiing, then as a feedback, also this type of very simple visualisation, "This is the training that you did, here are the kilometres that you did, and here's how your technique was this time". It would be a really good match.

**Interviewer** 34:38

Great. So, you've kind of answered the next question as well, which was how do you think they would respond to it? Do you think that the general population be quite receptive to having this information?

**Participant 10** 34:53

Yeah, definitely. But it would really need to be as simple as possible. I think because there's fine line there. If you go into too much details and represent the numbers and all that technical stuff, it becomes confusing. And then, you know, might even lead into "I'm not getting it, I ditch it because it's just too much and makes things complicated", even though we're trying to do just the opposite. So simple. Simple in terms of the information that we offer. And the avatar, I think it's a great idea, you know. You immediately get what the information is about. So much more instead of a table. It's a completely different thing.

**Interviewer** 35:36

Yeah. Cool. We've spoken a little bit about this one as well. But how would you think that users will use the information that they're provided? So if we were to give them the feedback? How do you actually think they would then go about implementing it?

**Participant 10** 35:53

That is interesting. And it also makes you think, when should you give that information to the person. I was quite a lot talking earlier about getting the feedback after my training, so that I could reflect back and think like, "Oh, did I do that?" But then, I'm pretty sure that if we make it clear, visual, easy enough to understand, then next time I go running, or my father would go skiing, there would be at least a tiny thought in my head from the last time reminding me to not take long steps this time, or-. But it really depends on, or makes you really think on, what's the best occasion to give the feedback to the person? So that it's not all also annoying. You know, somebody nagging on your technique while you're running, trying to just get a bit of load of yourself, that would not be that perfect moment at all.

**Interviewer** 37:03

Yeah. Okay. So extending on from that quite nicely do, foresee any concerns that people might have about the information that they're provided?

**Participant 10** 37:15

Yeah, I think the negativity is, for sure, something that comes into my mind, you know. "Is this device now telling me that I'm not good at what I'm doing? And why is it so negative?" So there would be the encouragement and the motivation, and that kind of positive feedback would also need to be there. Like, looking back to the picture that you showed me [Figure 3]. Now, it was telling me the error that I did, but in order to give a positive impression, it would also need to have something encouraging. So that kind of negativity is the concern.

**Interviewer** 38:00

Yeah. Okay. When we're talking about wearable technology and capturing data, where do you think that people will be willing to wear a sensor or some sort of monitor on their body?

**Participant 10** 38:18

It really depends on the size of a sensor and the sport that you're doing. But as such, if the sensor is small enough, then I think it gives a lot of tools to play with, because under your underwear, in your shoes, basically, wherever I would say, if the sensor is small enough. That's really the key. If you're trying to attach a device the size of your mobile phone to your leg, it doesn't really work. But if it's the size of a small coin, yeah, why not?

**Interviewer** 39:06

Do you see any potential barriers or limitations to using wearable technology? Specifically for assessing movement quality?

**Participant 10** 39:18

For instance, if I think that the limitations, well, the distraction or the fact that not that-. Firstly, there are people who don't want to be distracted, and secondly, there are occasions when you don't want to be distracted. I could think that that would be-, the distraction part would be the biggest limitation.

**Interviewer** 39:50

And then any potential facilitators to using wearables, so ways we can encourage people to use them to assess their movement?

**Participant 10** 40:01

What do you mean by facilitators?

**Interviewer** 40:03

So ways in which we can encourage people to use wearable technology, so something that we could do that makes someone think "I want to assess my movement quality. Yes, wearable technology is the way forward."

**Participant 10** 40:18

Yeah. Well, it comes down to the communication and communicating the benefits that this brings to you. And then making sure the device does what it what it promised.

**Interviewer** 40:30

Yeah. So there's that trust element as well?

**Participant 10** 40:33

Yeah. And the communication, so that it's very clear for me to understand "What do I get with this device? And what am I paying for?" Basic stuff that we hear, think, every day. But really, it's the basis for everything.

**Interviewer** 40:50

What are your thoughts, then, on introducing something like this into [company] products specifically?

**Participant 10** 41:00

I think the movement measurement overall, would really well complement our offering. Because it's there. We're doing sports, and part of sports is also the movement. But I see a lot of, especially in the hardware technology side, a lot of challenges that we still need to overcome. And when I talk about these, I mean, things like the sensor and sizing, the price of it, the communication of the sensor, or the sensors between each other, so that instead of just numbers, we can really get an overall picture. So the hardware side is another, size and prize and all that. It's something that we would need to find solutions to, and then the whole interpretation layer that the visualisation, what do we get with it? What what do we do with the data, basically? But by this, by finding so many challenges, I don't mean that it wouldn't fit just the opposite. I think it would fit really, really nicely.

**Interviewer** 42:24

Yeah. Okay. And then finally, any other thoughts or comments that you think might be useful?

**Participant 10** 42:35

I don't know if I have anything useful for you. But I do want to mention that it was nice to see the visual sketches, and especially the third one that you had [Figure 3]. It made me think even more the role that this could have for [company]. So, as always picture tells more than words.

**Interviewer** 42:57

That's why they're in there of course. Just just something to get the mind working.

# **Participant 11 – Sales**

Thu, 9/8 11:12AM • 58:39

**SPEAKERS**

Interviewer, Participant 11

**Interviewer** 00:11

Okay, right. So just a general introduction to tell you that there's no right and wrong answers. We're basically just really keen to get your thoughts and your perspectives on this project. Everything's confidential and anonymous. And then just to tell you a little bit about what we're doing, basically we're looking at using wearable technology to measure and assess how well people move. Before we get started with the questions, could you please just tell me a little bit about your role at the company? And then also, tell me a little bit about what you do outside of work?

**Participant 11** 00:55

Yeah, well, I've been working for [company] almost 15 years now, and I take care of all our B2B business in Finland. That includes team sports, or the national teams in football, ice hockey, etc. and, of course, all the way down to small juniors, who want to purchase our products. And besides that, of all our education products, which are sold to schools, and maybe to military, and to universities, and so on. And also other B2B health related projects in companies or corporates who want to buy our products to their employees. So I take care of all that. There's lots of different trainings, so I teach a lot, like, professionals, but also end users from a company, or coaches, how to utilise the products. Quite big role. And I also have our marketing, with PR things when needed. And outside of the job I like to do different sports, of course, it's kind of natural in this work. My background, I'm a PE teacher. So I am used to doing all kinds of things and-. Golfing and ice hockey, I just played ice hockey this morning, and gym and all kinds of stuff. Of course, just normal [unintelligible] and gardening and stuff like that.

**Interviewer** 02:46

So something we're going to talk about is the concept of movement quality. So just that term itself, 'movement quality', how do you interpret that? What does that mean to you?

**Participant 11** 02:59

First, my mind comes to quality or the intensity of the moment and what the people are actually doing and how intensively they are physically active. That that's my first impression of that phrase. I don't know what you're actually meaning with it.

**Interviewer** 03:22

So I'm not trying to tell you, it's more how do you interpret it, but it all comes round ultimately to having people moving well, again, we'll dive into that and it'll become a bit clearer as we go through this. So speaking of moving well, what sort of things do you think might help somebody move well, first of all, during daily life? So we're talking there about, you know, you just come to work, and you're at home, I think you just said about gardening, that sort of thing. So when you just living everyday life, what sort of things help you to move well?

**Participant 11** 04:04

Yeah, I think that it all starts with the basic physical activity and I give lots of lectures about this. So my strong opinion that the key point is that you need to have your basic physical activity in certain level before you can even start dreaming about being fit or getting into shape. You have to have your daily routines so that you are active enough and you need to pick those small pieces of physical activity during the day. You can choose, it's always a choice. You can chose to be, so that you walk or you take the bus, or you can ride your bike, or you can take a car. You can take the stairs, you can take the elevator. All these small things are really essential in terms of like, even if we go deeper into quality of life. One example, now that you have a rowing machine ad on your laptop, I quite often tell this story. This is how the physical activity or the exercising is nowadays. I heard this from our professor at Jyväskylä [University], back in the 90s. He said that people's physical activities gone this way that, there is a man in Finland. He spends his summer holidays on an island, and from the island, he takes a motorboat to the land. And then he drives his car to gym, and at the gym, he does rowing machine. Everything is so organised, and that is a big obstacle for people being physically active enough. One good thing that COVID did is that people notice that, "Okay, I can go for a walk, I can go just straight out my door and even do some activities at home". That's the key. That's the foundation in my opinion.

**Interviewer** 06:27

And I think that's really important, and, again, that's something that we'll come back to a little bit in the next few questions. Being a little bit more specific then, what type of things do you think help people to move well, when they're actually doing that formal exercise?

**Participant 11** 06:43

Yeah, it's a good question, because everybody should have the knowledge that it's good for you, but they need to feel confident about it. Other thing is, they need to feel that "It's good for me, and I'm good enough to participate into this". And the other other thing is that they need to think that it's worth doing it. Like, "It's worth spending my time being physically active". And then, if they come to our business, and what's going to give you nice feedback about your activity. Of course, I need to think that helps, and I know that it helps. It doesn't help everybody, but it helps quite many people.

**Interviewer** 07:50

So I think we kind of covered the next question there. Because we spoke about, not only what helps people, but the next question is about what influences people. And I think you've kind of touched on that a little bit with talking about modern living. Have you got any additional thoughts on that one? What might influence how well someone moves?

**Participant 11** 08:13

Yeah, it's so strongly connected to the motivation and where the person gets the motivation. So that is something that needs to be supported in every possible way. Of course, I understand that not everybody gets the good feeling from sports or being physically active. No, they don't get the endorphin or anything like that. So we need to understand also that we can't help everybody. But we can help, or we can support lots of people. And one common problem is that people think that you need to be exhausted in your physical activity, or it doesn't help at all. That is so strange. People don't think that you can even burn the calories by walking and even do it every day.

**Interviewer** 09:34

And I think, actually, just to expand on that, people think that to get the benefit, they need to be exhausted and because on a certain day, perhaps they haven't got that motivation to push so hard, they then don't do it without realising that actually, you don't have to do that to be active. You can just, as you say, walk, and physical activity doesn't have to be a chore and doesn't have to feel like effort.

**Participant 11** 10:03

Yeah, that's true, and that is a big, big problem. It's all over the places, if you look, TV programmes, or social media or whatever, they're all full of-. They are torturing people to lose weight and so on, but it usually doesn't help much.

**Interviewer** 10:26

So what benefits do you think that people could have by improving how well they move during exercise? So if you think about technical ability or something when they're doing the exercise, how do you think moving well would be of benefit to people when they exercise?

**Participant 11** 10:47

Yeah, like, what could they get from-? Yeah, well, I think these things are all connected, if you start to be physically active, next thing you'll get interested in is sports. You'll start doing even exercises, and that relates to, quite often, better eating. These are all connected. And if you do this, you notice that you start sleeping better. And the whole package comes. And my strong opinion, is that the normal physical activity is the foundation. And then you can start. Of course, somebody can start with the food, and put that in the order and then build something on that. But anyway, you don't get into shape by eating well, you still need the physical activity.

**Interviewer** 11:43

So when somebody is trying to improve how well they move during exercise, and this is-, if we think of it more on the technical side of things. So your positioning when you do a squat. Or how efficiently you run. That type of thing. What information do you think it's important to receive when you're trying to improve how well you're moving?

**Participant 11** 12:14

Okay. Yeah, I think, from running, it's quite easy to give the feedback, especially if your maximum heart rate is known quite well. And then, for example, [company] watch can give you the feedback. How well did you do? Or what's happened there? But if you're in the gym, in the beginning, I think that you need some guidance there if you don't know anything about it, because quite often, especially women, they just go to the gym and they just do it, and they don't feel anything. They don't know how to push. You don't need to push all-out, but you know how it goes. But some guidance is needed there. I don't know if I answered your question or-.

**Interviewer** 13:15

If you're giving that guidance, what sort of methods are you thinking of in terms of getting that guidance? And what sort of information is that guidance giving do you think? Possibly even going back to your PE teacher days, were there any particular things that were really important to focus on when trying to get somebody to learn a technique?

**Participant 11** 13:49

Just the foundations, that when you're starting to do this, the basic technique things, you don't hurt yourself. And then how many reps you should do when you start doing it. And then how many sets and and what kind of feeling your should get into the muscle to start getting-, if you want to improve it. Of course, you can hang around there to. Just in the last week I guided one lady in the gym. She's been around there like two or three years. She likes to go, but just hangs around and does things and I tried to teach her, "Okay, you just need to feel that the last two or three [reps] are quite heavy, and then you'll start to improve", and then she understood what's the game.

**Interviewer** 14:50

I think that's quite common, actually, that people do the same thing on repeat because they don't know-.

**Participant 11** 14:57

They don't know what to do next and you should change, even if you don't have any programme, you should give different stimulus to your muscles after a couple of weeks. And this information, I think that's all over the place. It's kind of same thing as with running or whatever. The info is everywhere, but you're just-.

**Interviewer** 15:25

It's just finding it. Like filtering it out?

**Participant 11** 15:29

Yeah, exactly. Yeah, there's of course all kinds of information. Not so good [information], also.

**Interviewer** 15:36

So we're exploring ways to help people understand how they move, and how they can improve the way that they move during exercise. So currently, there are no affordable, accessible and effective ways in which this can be achieved. And by that, what I mean is that, if you think about some of the ways that people can improve how well they move, people might have a personal trainer, which obviously comes with a cost. Plus, you have to generally go to a facility for access to the personal trainer. Maybe if you're working with a with a physiotherapist, you have to go to a hospital. Again, there might be a fee, or you may even have a lab-based analysis method, which maybe has the camera systems and they cost a significant amount of money, and only really are accessible to elite athletes, researchers, that type of thing. So what we're looking to do is use wearable technology to hopefully change this. And explore ways in which we can use wearable technology to measure movement quality, but also ways in which we can then make the data that we collect easy for people to interpret, so that then they can improve the way they move and implement that safely and effectively. So first of all, are you able to give me some examples of feedback methods that you're aware of the help inform people, first of all, how much activity they do?

**Participant 11** 17:15

Yeah, yeah. If we think about like, our products, or-?

**Interviewer** 17:19

Yeah, so that's one example. But more expansive than that?

**Participant 11** 17:24

One thing, if you think about basic physical activity, I think that, like with our products, that your own goal, that is a good starting point. And people find it interesting and they find it helpful. Of course, if you are training a lot, you can easily reach 200% [of daily activity goal] every day. But those who really need the guidance, it's a good goal for for everybody. And when these features came like, maybe, almost 10 years ago, people got so excited that they wanted to get their 100% [of daily activity goal] and they needed to go for a little walk in the evening if they didn't reach it and I think we have done great help for many many peoples. People who are feeling better now. They are learned how to be physically active. One thing for wearable is that it you can live without it, but it can help you to start doing things better. Of course, if you want to follow and become better, of course, after that it's good to start following, for example, your training load and things like that, because now it's the time of the year that people are, after summer holiday, start working out like crazy. And that lasts for a couple of weeks. If you don't listen to your body or listen to the technology, which could guide you to take a break and have days off. Yeah. The next one then is, can you think of any ways for measuring how well people move? That is a more difficult thing. Everything that is heart rate based movement, that's quite easy. "If you're this age and you're in this shape, you should move this much", maybe during the week, or two weeks period, just to have this package. Then you should be-, if you wanted, really, to improve your fitness, you should be improving, and then after, then you have an easier week. So like in the endurance training, I think it's quite easy, and we are quite good at that. But if we go to the gym environment and that kind of exercises, it's more difficult. Definitely, it's more difficult. And I don't even know how it should be done. Maybe this could measure the quality better during the gym exercises, but they don't know how much weight there is, if you're doing something. That is really, really difficult.

**Interviewer** 20:45

So something we've been really drawn to is the use of visual aid. So how do you interpret that initially?

**Participant 11** 20:59

Visual aid in wearable, or anything?

**Interviewer** 21:04

Anything.

**Participant 11** 21:05

Yeah. When you gave some background info earlier, I was just visualising, "Oh, there should be a platform where you could easily have like, high quality support for your training". Like, if you're doing squats, you would easily have access, maybe on your phone that, "Okay, this is the way you should do it". And then maybe you could use your phone and you could get a new idea. You could film your squat, and it goes to app and you get guidance [on] what's going well, and what's going wrong. If your back is in the wrong position. Something like that could, in the future, like AI platform. Definitely, quite good.

**Interviewer** 22:02

Yeah. So hold that thought, because we'll come to-.

**Participant 11** 22:06

My daughter is studying AI. So I always talk to her about what's possible. It's so much interesting.

**Interviewer** 22:14

So first of all, what do you think about-. What do you think people would think of visual aids to get feedback? Something like you just described. What do you think people actually think of that?

**Participant 11** 22:27

I think it would be really helpful, especially in the beginning. But people learn to do things. But of course, there's always new things and hundreds of different movements. So for the beginners, of course, or those who haven't been physically active, who have just been sitting on the sofa for the last 10 years, I think then it will be really helpful. But it should be really smart guidance that what you should've done. There should be quite nice background information about the person, what he can do. What if there is back problems, or medication, or whatever. But I can really see the future in this actually. Maybe there is even [a technology-based movement quality learning aid] at the moment, I don't know.

**Interviewer** 23:30

So do you think that you would like something like that?

**Participant 11** 23:35

I wouldn't.

**Interviewer** 23:36

Because you've already got that experience?

**Participant 11** 23:39

Yeah.

**Interviewer** 23:40

Okay. Right, so if we're going to provide feedback, visual feedback, and you're trying to get somebody to move better, do you think that the information be best delivered only as a visual? Or do you think that it would be best to to accompany it with some other type of feedback, such as audio, or text, or vibrations from the wearables?

**Participant 11** 24:26

Depends on the environment. Audio would be a bit difficult in the gym, for example, but audio would be nice in a run, for example. You could get info for the road. If you have uploaded roads that you're running, there's uphill, the guidance could give you that, "Okay, you should take shorter steps when you're running now to improve your performance", or things like that could be done.

**Interviewer** 24:57

Okay. So it's really depends on-.

**Participant 11** 25:01

It is, yeah.

**Interviewer** 25:03

When we're giving feedback, what sort of devices do you think would be best? And again, this might depend on the environment. If you're trying to get visual feedback on your movement, what sort of devices do you think would be best?

**Participant 11** 25:23

At the moment, I would see the phone being the device, because of the bigger screen and ability to show videos and stuff. Maybe a watch could be, but it has to be quite simple, then. Even [though] the displays are getting better. I would say the phone, like the app giving you the info at this point.

**Interviewer** 25:59

How much detail would you expect the feedback to provide? So, you do your exercise, whatever that might be. Do you think it needs to be detailed or not?

**Participant 11** 26:16

It depends on the on the exercise actually. I'm now stuck with the gym, because it's so difficult, because you don't know the weights or exactly everything. But, for example, like running, why not if it can help that person? Or swimming, which we can measure quite well nowadays. I think it would be interesting, what happens with your strokes and-. But if you think about the big picture, those who we are trying to get physically active more, or they are just beginning, not too much information, because, well, I think they could get lost in there and it's kind of hard to understand it.

**Interviewer** 27:23

Okay, so what I'm going to do now is I'm going to show you an example of some visualisations that exist, either in products or by concept. I'm just going to show them to you get your thoughts on them, what you think it shows, and then we'll have a conversation about it. So the first one is this one at the top [Figure 1]. Just have a look at that for a moment. See if you can work out what it shows or what you think it shows, and then we can have a conversation about it.

**Participant 11** 28:02

Okay, so throwing something I guess, like it says here, but what is it? Gold-standard throw trace is the green one. I don't know what he is throwing. I don't know.

**Interviewer** 28:40

Can you roughly work out what you think it shows though?

**Participant 11** 28:45

Yeah, it's shows how the arm goes. It goes there, and then it goes back [demonstrates throw motion]. But I don't know why it goes-. How can there be a gold-standard?

**Interviewer** 28:59

So if I just, just for context, this isn't to scale. It's just the orientation, so it's not as much of a-. But yeah, basically, so the the green line is the gold-standard. It's what you're trying to achieve. The coloured line is then the trace that the the arm did during the throw while wearing a wearable device, and the colours then tell you the speed. So yeah, it starts off slow because it's not moving. Accelerates, stops, and accelerates through. The pink area then just gives an area of tolerance. So the idea would be is that even though the green is the gold-standard, if you're within the pink zone it's acceptable. Outside is then not so good. What do you like about it and what do you dislike about it, if anything?

**Participant 11** 30:01

Yeah, I think this is quite clear. But one thing is that who knows, what is the gold-standard? Because, is it based on, for example, if there's a small person or a big person, is the moment same or not? I don't know. But somehow somebody has decided that this is the standard. I mean, this is quite clear. Yeah, even I don't fully understand how or why it is important the way it goes back here, but it doesn't matter, I guess.

**Interviewer** 30:48

Is there anything that you dislike about it?

**Participant 11** 31:01

Well, there's no numbers.

**Interviewer** 31:11

Okay. So just have a little bit more information for ease of assessment, then I guess?

**Participant 11** 31:19

Yeah.

**Interviewer** 31:21

So if I move on to the next one then [Figure 2]. This is an Apple Watch. Just again, have a look at that. See if you can work out roughly what it shows, and then we'll have a conversation about it.

**Participant 11** 31:37

Yeah, I would guess that these are-. I've never used Apple Watch, but I think somebody has shown me, but I guess these are like heart rate zones? It's been in the blue that much, and then in the green that much, and then red. Or maybe not, but it's activity. I don't know. I think it's somehow related to the intensity, but I don't know if it's heart rate, or is it some body movement.

**Interviewer** 32:06

So actually, the red one is just your total activity. How much you've done in a day. The green is how much activity you've done above a certain intensity threshold. So that's like brisk walking or higher. The blue is your time spent standing. So with the blue, basically, every hour, you have to spend five minutes at least standing ,and then as you tick the boxes for that, then that one goes round. What do you like about it? What do you dislike about it?

**Participant 11** 32:09

Once you know it, then it's okay. For me, because I'm so fixed into our heart rate zones, then it's-. And I would like to know what are the MET limits, or what are the limits between the zones. Like, this standing, okay. And the red one was above the-. No, the green one was above a certain threshold. Okay. I don't think it's very clear, at least for me.

**Interviewer** 33:20

Yeah. That's a common theme actually, is that the the red is perceived as high intensity in this environment, or that something wrong? So potentially, that's something to bear in mind for colours. If I move on to the next one then [Figure 3], and this is where you'll probably be quite interested because it's similar to what you've described. So this is a an individual who's doing squats. He's wearing a couple of sensors. You can see this one there. He's wearing one on his chest. He's wearing a couple of other ones as well. And then this is the app user interface. Have a look at that and see if you can work out roughly what it's trying to show.

**Participant 11** 34:06

Yeah, it clearly shows the first challenge he's having, that he's bending forward way too much and it's nice that the app shows that, and then what else is there? I should have my glasses on. These are degrees. Spine flexion, okay, this is like this way [demonstrates a forward bend]. Okay. And is there between the red-. I can't see what it says there. Should it be between these [red lines] or under?

**Interviewer** 34:52

Yeah, so it's between those.

**Participant 11** 34:54

Yeah. I think this is good info.

**Interviewer** 35:07

Yeah, and I think you've understood it quite well, as well. You haven't had any problem understanding that?

**Participant 11** 35:15

No, no.

**Interviewer** 35:18

What do you like about it? What do you dislike about it?

**Participant 11** 35:24

I like this. This is a good one [points to top portion of Figure 3]. And it says that, there is-. I don't know what this does actually, maybe it shows the angle [points to ring outside of avatar on Figure 3]?

**Interviewer** 35:41

So basically, as he bends down, this black marker is relative to his angle. So the idea is-.

**Participant 11** 35:47

You should stay there. Be funny if he was there [points to limit of being too upright]. So it's maybe falling backwards? Yeah, shouldn't be the-. Yeah, actually, it's okay, like this [forward limit], but it's unrealistic to go like-. The full would be maybe here [indicates a smaller range of motion tolerance]. So the scale would be like, I don't know. But the 360 degrees, of course, it's clear when you understand it. And this marker [black marker] maybe could be more clear, but now that you said that it is there, of course, it's obvious that's what it points to, that it's his current angle there. And these are, are these reps here.

**Interviewer** 36:42

Yeah, so those are his previous repetitions. So essentially, this one would then go down there once he's finished the rep and you'd expect them to be above that red line, because he's gone too far. And then you have the option then to change. So that changes the view, so obviously it's shown from the side at the moment, and then that also changes the angle that you're looking at. So at the moment, we're looking at how far he's bending forward. Maybe you could change it to be like the knee angle or something like that. So yeah, that's the other one. Is there anything we dislike about it?

**Participant 11** 37:24

Not really, no, I think this is a good start anyway.

**Interviewer** 37:29

Okay. So having seen those, has it changed your thoughts at all on possibly using visualisations for feedback?

**Participant 11** 37:49

Yeah, the first example with the throw, if in that, if there is also, if we go into sports, there would be a video taht adds to that, it shows the movement and then it can show the perfect person also, and then the graph. That would be quite helpful I would think. But, of course, that's in sports.

**Interviewer** 38:32

Okay. So, expanding on that then, how do you think that the general population would find the use of visualisations? So just your everyday person? Or maybe the less elite level of [company] customers?

**Participant 11** 38:55

Are we talking about performance or just-?

**Interviewer** 39:00

Not so much performance here. So it would be more

**Participant 11** 39:03

Active? Or trained person?

**Interviewer** 39:05

Yeah, if we think maybe recreational athletes to possibly semi-professional, we're kind of in that range. Or even the person that buys a [company] device, or a device, just to stay impartial, because they want to become more active. So it could be like the aspirational recreational athlete.

**Participant 11** 39:29

Yeah, maybe something. Because the basic stuff is quite good at the moment, the feedback of your daily physical activity, but maybe to give more to the people, we could give more, like precise things: "Hey, you were sitting three hours there. Was there any chance for you to be any active during that period?", "How was your day there?", "Why did you sit like this?", but dig more into that? "And then you did your job or workout, you spent there 55 minutes, but your average heart rate was 115bpm. So how much time you're spending on your phone?" or whatever. I mean, it's just not these kinds of things, but we could dig more deep into the routines, and maybe we could help. But there has to be a positive angle in that. It has to be a really-. You have to be really sensitive on how you put the words there, or whatever the feedback is.

**Interviewer** 40:56

Yeah. Okay. So, if I think about the examples I just showed you, and you think about the lady that you helped in the gym the other day, do you think someone like her would appreciate something like those visualisations?

**Participant 11** 41:18

Definitely. With the squat example [Figure 3], yeah, definitely.

**Interviewer** 41:25

And do you think the level of detail of that one was sufficient? Or do you think too much or too little?

**Participant 11** 41:32

I think that was all right. Yeah, it's a bit hard to think of somebody from somebody else's point. But those basics, basic things that you-. Basic technique. Plus, you have to-. Of course, there needs to be some data that how many reps you should do and things like that, I think that would be really, really helpful. But at the same time, it has to be really-. Because we are all sports people, it's really hard for us to be in those boots, that you haven't done anything, and you're scared of everything, and you're worried [about] being hurt, and you are worried about not being able to do anything, and you're ashamed to go there [gym or sports facility] and you don't know how to be there, or you don't know how to use the machine, or you don't know anything. You don't know how the locker works. And you don't know anything. When planning these, somebody should have people from-, non-active people, to help build this, definitely.

**Interviewer** 43:18

So how do you think that the users would use the information that they're provided? So again, using the example of the the lady in the gym, perhaps she gets information about her movement. How do you think she then uses that information?

**Participant 11** 43:37

Yeah, I think she would use the information. We can't underestimate, people are learning fast, and these are not very difficult things. I'm quite sure that now that we checked those couple of machines and couple of moments, I'm 100% sure that she remembers next time how these should be done. So using the app like that, with the pictures, with the squat, and things like that, it's kind of hard to imagine that somebody uses it for a long time. Maybe somebody needs the support and needs the help for a longer period. Again, it's a bit hard to imagine.

**Interviewer** 44:32

Yeah. When we're assessing movement quality, do you foresee any concerns that people might have about the information that they receive?

**Participant 11** 44:48

I don't know. If you think about endurance training, for some people, I'm quite sure that it would be kind of hard to believe that, "Is this really working? Or is this good for me? And how do they know that I should do this?", and convincing them. People are always after quick results. So, how can we sell the idea that there is no actual quick results available, that you just need to do your tasks, be physically active, and do these things, then the results will come. So that is at least one thing that the feedback that system gives you, it has to be really constructive. Based on what? "Okay, last two weeks you have done these [exercises]. Way to go. You're on the right path".

**Interviewer** 46:31

Now we're going to talk about wearing monitors. So if you're wearing a wearable device, and we're trying to measure movement, where do you think people would be willing to wear a monitor on their body?

**Participant 11** 46:49

If they need to have to feedback? Or are you meaning like, on the squat there? Or just on their own exercise in normal life?

**Interviewer** 47:05

More so for exercise specifically?

**Participant 11** 47:09

Yeah, but not in the research, just in a normal-?

**Interviewer** 47:12

In a normal sense, yeah. So they're in the gym, or they go running, or they go swimming, or something.

**Participant 11** 47:16

Yeah, well, the wrist is quite normal and common. That's easy to check the feedback that it gives you a during the exercise from there. Other thing would be the audio feedback, because quite many use the headphones or pods, or whatever. I don't know what else it could be. But then it's, of course, sports related, very unique to have the sensor, and you can wear the watch, but when we think of about normal people, thew atch is still quite good. So if we had somebody in the gym trying to learn a movement, do you think they'd be willing to wear a sensor on other parts of their body, just for learning that specific movement? So do you think they'd be willing to wear one on their ankle or their leg? I would think so, yeah. But if I think about people in that need, they should have somebody to guide them still. Like learning on your own, putting a new sensor on and using your phone? That would be maybe too much for for a beginner, but if there is somebody helping out, and using the sensors as part of the learning process, then a bit more natural. But anyway, if I think about learning, for example, golf, if there will be a really easy to use sensor that I could buy and I could have either in the driving range, for example, to measuring my movement and it would give me good feedback, I would definitely use it. But then you need to want to learn the thing.

**Interviewer** 49:26

Do you see any potential barriers to using wearables specifically to assess movement quality? Do you see any sort of limitations to using them?

**Participant 11** 49:46

Well, if we start with the physical activity, they could be more precise than they are nowadays. Like, dividing sitting and standing, it's still difficult. Measuring steps from the stairs, for example, that's kind of difficult. And that could help the person to perform better, and to get more excited about the measurement, if he could get more precise information. The basic stuff is quite good now, but the better it is, I think the better it moderates the user.

**Interviewer** 50:49

Do you see any potential facilitators to using wearables when assessing movement quality? So these are ways in which we can encourage people to use wearables? Sorry, I don't, understand the question. So if we're trying to get people to use wearable technology, to assess movement quality, how do you think we can actually do that? What can we do to make them think, "Yes, I want a wearable device and this will help me".

**Participant 11** 51:26

I think the basic idea is to, honestly, tell them that this can really help you, and this tells you what you do and this quite well tells you what you don't do, and how your daily life is, and it can help you to make those little choices that can support you on the way to-. Like we had the first devices measuring physical activity, people were crazy excited about it. And there's still millions of people who need that push. So, yeah, if you think about healthcare, it can be so the doctor or nurse says that you're so out of shape and so inactive that you need to wear this. Not that way, but it has to be the other way. There is a possibility for you to start being more active step by step, and then think about, like, the motivational aspects. And then they really need support, and supportive questions that, "How do you feel now that you have been physically active? Do you sleep better? And what else has happened?", and then it starts growing. So there are big possibilities.

**Interviewer** 53:11

Okay. What are your thoughts, then, on introducing some sort of movement quality assessment and feedback method into [company] products

**Participant 11** 53:30

Like I said earlier, more precise information. If we start from the physical activity, more precise information there, that would be beneficial. And then what we have so far is, like, the swimming strokes and that kind of exercise-based ideas. That's nice. What else in the gym? I guess there could be, or if I think about the throw example, so just sensors can do anything, but then we just need to find out what is really needed. You can't have everything in every sport and every technique. So it's hard to say. I'm sure that there is something.

**Interviewer** 54:29

Yeah. But do you think it fits within the [company] business model? Do you think there's something that there is an opportunity for it? Something getting people to move better?

**Participant 11** 54:53

In this, I would concentrate more on the physical activity side, not the sports side.

**Interviewer** 55:05

So the general person?

**Participant 11** 55:06

Yeah, yeah. I would see the better, or the bigger potential anyways in the Sales space there. To be able to give more feedback and encourage to be physically active, I think that would be a nice thing. And that goes all the way, of course, to the sleep measurement and that stuff too. But it's kind of same thing. And in the sports, if we go to the top top level, it's really, at this point, it's kind of hard to see what could be the-. Of course, we could, for example, I don't know if you know anything about cross-country skiing, but we could measure the strikes and how the poles are moving, I guess quite easily. And I guess they would be kind of-. It would be kind of interesting, but there is such a small amount of people who would utilise that. So it's hard to think. And then the gym exercises, those will be related to the app. And what, why not? I don't know if it needs any additional sensors, or I guess AI could help to scan the person so well that it could help measure the angles and so on. Yeah, why not?

**Interviewer** 57:03

So last thing, then, have you got any final thoughts or comments at all related to this that you think might be useful?

**Participant 11** 57:13

I guess I have talked a lot. I would be happy to find something for the sport side, but obviously, I don't know why, but my thoughts are more in the average people and how to support their physical activity moreso. And I think that's where the business has its biggest potential. Also, like healthcare related issues with this. If there is a big hospital and they want to do-. It doesn't need to be any research. They just want to support their client, customers, to be physically active and live healthier life. That kind of quality body movement, measurement support or whatever it is, I think that would be really, really nice.

**Interviewer** 58:31

Alright, I'm gonna stop the recording.

# **Participant 12 – Sales**

Thu, 9/8 12:43PM • 26:28

**SPEAKERS**

Interviewer, Participant 12

**Interviewer** 00:10

Okay, so as spoken off the recording, there's no right or wrong answers in this. We're just really keen to get your perspectives and your thoughts. Everything is confidential and anonymous, so it's my eyes only, or ears in the case of the audio. And then, just give you a brief introduction of what we're doing, as you probably already aware, basically, we're looking at movement quality and looking at how well people move. So, before I get started with the questions, could you just tell me a little bit about your role at the company? And then also, tell me a little bit about what you like to do outside of work in your spare time.

**Participant 12** 00:52

All right. My role at [company] is, I'm heading up our Sales and Marketing in the Nordics. We have three sales subsidiaries here, and I'm Managing Director for those three companies. In my spare time, I have two kids; a lot of time with that. And work of course. But I run and swim and do some gym workout, and I do racing in Swimrun. Where we go-.

**Interviewer** 01:25

Like triathlon without the bike, but a little bit more?

**Participant 12** 01:27

Yeah, sort of.

**Interviewer** 01:30

It's a little bit more cross country?

01:31

Yes, it's a lot more trail running. And then you go from swimming to running to swimming to running, so you don't just do them once like triathlon.

**Interviewer** 01:38

I'm vaguely familiar with it, but sorry, I didn't do the justice it deserves, actually. So as I mentioned, we're talking around movement quality. That term 'movement quality', how do you interpret that? What does that actually mean to you?

01:59

Well, the first thing that comes to mind is technique. I used to swim competitively, and there, my thought of quality of movement would be how correctly you do the different strokes, and there, it's invested quite a lot of time in videoing and different kinds of technique to try and look how well people are-, how efficient the movement is.

**Interviewer** 02:24

So what do you think helps people to move well, first of all, during everyday life? So in the context of, you're coming to work, you're doing your food shopping, you're with the children, what sort of things do you think might help you move well on a daily basis?

02:45

I think in general, having some kind of strength and conditioning routine, because then you're aware of the movements that you do. If you're training yourself, if you don't ever do anything, then you're maybe not aware that the way that you lift or the way that you walk, it's maybe not the best way for for your body and can cause some stress.

**Interviewer** 03:11

And then more specifically towards exercise, then, when you're actually doing exercise, whatever that might be, what helps you move well during exercise?

03:24

One thing is to concentrate on what you're actually doing. A lot of people run listening to music or doing something else, and then maybe you're not thinking about the way that you're moving. And then I think if we go to something like strength training, then to have a coach, or to workout in a group where you're able to get feedback about the way that you're doing the movements.

**Interviewer** 03:48

Okay. And then what do you think influences how people move? How well people move? So subtly different question, this is more other factors that could influence how you move. Like, externally.

04:09

Maybe what kind of person you are. If you're the kind of person who's always in a rush and wants to do things quickly, then maybe you don't think about the way you're doing things, also in the way that you're moving, rather than if you're a person who more takes your time and thinks about-. So personality, perhaps.

**Interviewer** 04:25

Okay. What benefits do you think people could have by improving how well they move during exercise?

**Participant 12** 04:35

I think the biggest one would probably be that there would be less injuries.

**Interviewer** 04:39

Yeah. Okay. And, obviously, you've said already about-, you alluded to earlier about performance, that efficiency as well. So when you're trying to improve how well you move during exercise, what information do you think is important to receive?

05:01

I think it's important if we think about strength training to be somewhere where there's mirror where you can see what you're doing, but then also to have feedback from from a coach or a PT or someone like this, or even someone you're training with. Because there might be things that you don't-, that you might think that "Okay, my back is straight", and in fact, it's maybe not straight.

**Interviewer** 05:23

Okay. So, we're exploring ways to help people understand how they move, and how they can improve the way that they move during exercise. Currently, there's no affordable, accessible and effective ways in which this can be achieved. So by that, what I mean is, you know, you've said about personal trainers, but there's obviously a fee that comes with that. And then the accessibility as well; you generally have to go to a facility like a gym or a personal training studio or something to get access to that. Maybe like a physiotherapist working through an injury; again, that comes with a cost. Or even like a lab-based system, so like there's camera-based systems, but there's a lack of accessibility to that, reserved more for, like, high level athletes are researchers. And it's not really practical to do that every time you want to train. So something we're looking at is the use of wearable technology, and how we can use wearable technology to measure and assess how well people move. But also the ways in which we can take that data and use it and deliver it as feedback to end users in a way that they can then interpret it and implement it safely and effectively. So can you think of any examples, first of all, of feedback methods you're aware of for measuring how much activity do people do?

06:51

Well, the wearable devices have activity measurement, which is based usually on movement. So the gyroscope that's in the device. Or then sometimes it's based on heart rate, so how elevated your heart rate is. Then it kind of gives you that level of activity; the strenuousness of the activity.

**Interviewer** 07:16

And then how well people move?

07:19

That's a bit more difficult. Yeah, I can't really think of anything that the wearable-. Well, I suppose if there's like a training regimen that's coming from the watch. Mostly, I would say it's linked to heart rate, that you have the feedback to train within a certain heart rate, that feedback you can get from the device saying that, "Okay, now your heart rate is too high", or, "Your heart rate is too low", that if you're running into hills, you need to go a bit harder to get into that right heart rate interval, or if you're supposed to be doing an easy workout to get the feedback to stay within the low heart rate levels that you're supposed to be in.

**Interviewer** 08:03

Okay, so something we're really drawn to is the use of visual aids. So first of all, what do you think I mean when I'm talking about a visual aid?

**Participant 12** 08:13

Picture or a video?

**Interviewer** 08:14

Yeah. So if we were going to use something like that, a picture, video, animation, that type of thing to give movement quality feedback to people, do you think that would be something they would like? Do you think it would be well received?

08:31

Probably. And I would think that in a lot of cases, that feedback needed is quite similar. For instance, if if a person needs to squat, it's probably certain things that you need to think about when you're squatting in order to do the squat correctly. That to have a visual of, "This is what it's supposed to look like and think about these three things when you're doing it", perhaps.

**Interviewer** 08:56

So would you personally like something like that do you think?

**Participant 12** 09:01

Maybe, yeah, I think that could be useful.

**Interviewer** 09:07

Do you think that if we were providing visual feedback to somebody, do you think it would be best to provide that in isolation? So they just get visual feedback. Or do you think that it would be better to deliver it with some other sort of feedback, like text or audio or vibration or something like that?

09:27

I think it would need text and/or audio, also. Visual on its own is maybe not enough.

**Interviewer** 09:35

Yeah. Okay. What sort of devices do you think would be best to provide feedback?

**Participant 12** 09:43

Probably the easiest is mobile [phone] device, because that's something that everybody has on them all the time. But of course, the screen is quite small. So in that sense, maybe like a tablet, for instance, they have a little bit bigger screen, but it's still something you can take around with you.

**Interviewer** 09:59

Yeah, okay. When giving this feedback, how detailed would you expect it to be?

**Participant 12** 10:09

I think if it's too detailed, people will ignore it. And then I think person's, probably, capability to, like, how many different things you can think about while you're doing something? I would say it's two or three, probably, at max. If you say, "Think about these eight things while you're squatting". Yeah, it's not gonna happen.

**Interviewer** 10:28

Yeah. Just to expand on that a little bit, do you think it's not necessarily the amount of detail but the delivery of it? Do you think there's-. Do you reckon it would be best just to keep things as simple as possible with a few things? Or do you think on that one, if you have those eight things, do you think there's a case to be made that you could just focus on the most important one or two, and then still have access to the others if needed or desired.

**Participant 12** 11:00

Now that you put it like that, I think it could be a good to have the basics. "This is the basics". And then when you've, let's say, you've done that exercise on five different occasions, and then you can make the assumption that "Okay, now you're familiar with these three things", that it tells you then you could move on to the next three things. So kind of build upon it in a way that's, for instance, in the lens of choreography, you don't start with the whole choreography, you start with a few elements and add on.

**Interviewer** 11:30

Yeah, okay. So, what I'm going to do now is I'm just going to show you a few examples of visualisations that exist in products. I'm going to present each one to you unprompted, just see if you can understand what it's trying to show. And then I'll explain a little bit and we'll have a conversation. And then we'll see what you like and what you don't like about it. So, first one is this one at the top, Figure 1. So if you just have a look at that, just take a moment and see if you can work out what you think it's showing, or trying to show.

**Participant 12** 12:23

I suppose it's showing the trajectory of a throw and how it should be, if it's perfect and where it lands on. There's 100 throws, and maybe the purple is all of the data points of what actually happened.

**Interviewer** 12:53

So, yeah, basically, the green one is what you're trying to achieve. That's the gold standard. And then the coloured one is what you actually did. That was recorded using a wearable device on the wrist, as they did this throw. The purple is basically an area of tolerance. So the idea is, while you're aiming for the green, as long as you're within that tolerance area, it's considered to be a good throw, and then when you deviate outside it, then you need to correct something. What do you like about it? And what do you dislike about it?

**Participant 12** 13:34

I find it difficult that it's circular, when it's talking about a throw that I would think is more linear. That I think makes it quite confusing. And also, the way that the the purple is shown, it's drawn in a different way than the other two. So it's difficult to understand that they're supposed to be showing the same thing.

**Interviewer** 14:01

Yeah. Okay. Is there anything you like about it?

**Participant 12** 14:10

I think that it's quite clear. The three different elements or colours are different enough and presented differently enough to understand which element is which.

**Interviewer** 14:18

Okay. Moving on to the next one, then this is obviously an Apple watch [Figure 2]. Okay, and what do you think it's trying to show?

**Participant 12** 14:26

Something to do with the amount of activity that a person has had, I guess. I don't know if they're different levels of activity.

**Interviewer** 14:33

Yeah. So this outer ring is basically the total daily activity. The green one is activity above a certain threshold, so some brisk walking or higher. The blue is basically standing time. So every hour you're supposed to spend five minutes or more standing. So as you check that off, the rings then go around and complete. Again, same questions, what do you like about it and what do you dislike about it?

**Participant 12** 15:06

It's quite simple. So that always helps. And visually, the circle is visually easy to grasp and pleasant to look at. Dislikes, I maybe dislike that there's more-, I guess you know what they are if you're using Apple Watch, but I dislike that there's no indicator of what those are. Like, there would be room to make an icon that actually shows shows what it is, or I guess there is an icon, but it's maybe not very clear.

**Interviewer** 15:42

Okay. And then the last one is this one [Figure 3]. So this is a person doing a squat. He's wearing sensors; there's one on his leg, chest, he's wearing a couple of other ones as well, but you can't really see them. And then this is the app user interface that is then giving him some feedback on his movement. Again, just see if you can work out what it's trying to show and then we'll have a conversation about it.

**Participant 12** 16:10

Well, there seems to be, again, values to stay within. And then if you're going outside of the perfect values and it gives you a prompt to change the way that you're moving. And here, it's prompting that he's bending too far forward and should be more more upright when he's squatting.

**Interviewer** 16:40

So just to expand as well, then, it records his repetitions, and that tells him how far through the range of motion he went on the previous ones, and then whether he exceeded the tolerance or not as well. What do you like about it? What do you dislike about it?

**Participant 12** 17:02

I like the guy.

**Interviewer** 17:07

The digital representation?

**Participant 12** 17:08

Yeah, because it makes it easy to understand what you're doing wrong, rather than just having a text around it. I don't really understand what the circle is.

**Interviewer** 17:21

So the circle, you can see there's a black marker, so the idea is that the black marker rotates around the circle. So as he goes through the range of motion, as he starts at the top and he bends forward, that black marker will rotate. And the idea is that the black marker is supposed to stay within the green zone, and then if it comes outside, that's when you get your text feedback there that says he is bending too far forward.

**Participant 12** 17:51

It's a lot of data on one screen. I think it would be better if it was just that top data. And then the second [bottom of Figure 3] could be if you scroll through, or maybe at the end of the workout. So I think at once, to think that you're actually looking at this while you're working out, it's too much.

**Interviewer** 18:07

Yeah. Okay. So, having seen those, has that changed your perceptions or your thoughts at all on using visualisations for feedback, specifically for movement quality?

**Participant 12** 18:25

No.

**Interviewer** 18:29

How would you think the general population would find the use of visualisations? And also, what do you think their thoughts would be on the level of detail and their ability to interpret what was presented?

**Participant 12** 18:47

Usually, I think the people making things go too deep into them. So I think there's a high risk that the detail gets too nitty-gritty, to the point where it feels not useful to the user that, like, I think it would be really important to to understand what are the most key points and focus on those. Maybe in addition to those that have an advanced level, where if the user would like more info, they're able to access it.

**Interviewer** 19:26

How would you think, then, the general population would respond to movement quality feedback? You've kind of touched on it a little bit already, but if we gave them the feedback, what do you think their general response would be?

**Participant 12** 19:40

I think that those that like tech would find it really interesting and be excited about it. But there are those users who, even the data that wearables nowadays give, they don't want. They think it's more important to just know your own body and how you feel, rather than interpret data that's given to you from a device, or don't like that their data is-. Like you're told how did you sleep, for instance.

**Interviewer** 20:11

Yeah. That's interesting. So we'll come back onto that one in a minute. If we give them movement quality information, how would you think that they'd actually use it? How do you think they might implement it?

**Participant 12** 20:28

The ones who are pro-technology, I think will take it into account in the way that they're moving and make changes. I think the ones that don't believe in it, if you can put it like that, who think they know best, then it won't have an impact on them.

**Interviewer** 20:51

So we covered some of the concerns that people might have. Where do you think that people will be willing to wear a monitor to capture their movement?

**Participant 12** 21:02

It depends on, probably, the size of the monitor, but I would think people are quite accustomed to wearing watches. I've seen some things where you attach something to your shirt, like your collar. That would probably be a reasonably easy place, or on your pants or shorts, like, waist, probably I think.

**Interviewer** 21:29

What about things like your ankles or your legs?

**Participant 12** 21:33

Yeah, probably, if it's not a big or heavy thing to wear them? I think so it probably wouldn't bother [people] either.

**Interviewer** 21:39

Yeah, it probably depends then, largely on what the actual device is like. If it's too big, then it's not so convenient. So this is where I'm coming back to something that you spoke about, the potential barriers to using wearables. So, limitations. I think we've covered them. What do you think about ways that we could facilitate the use of them, like actually encourage people to use a wearable for this sort of thing? Even maybe the technologically apprehensive ones. Or if that would maybe be a lost cause, maybe the others-?

**Participant 12** 21:40

Probably, I would say, maybe testimonials, and really focusing on the benefits of why you should use this. For instance, it's probably easy to find statistics on how many people are injured while they're training and what that causes. How many days they need to be away from work, or for how long they can't train, or things like this to say that, "With you having correct technique and what you're doing, you're able to minimise your likelihood of injury by X percent".

**Interviewer** 22:59

You said, then, about like being off work and stuff. That was quite interesting, because that suggests appealing more towards the general person moreso than-. I know you said about injuries and time off training and things like that. But it was quite interesting, you said about the time off work. Do you think that's something to really home in on to try and appeal to the general person and why-?

**Participant 12** 23:27

The reason I said it is, I've seen some statistics on that previously, or aside from mental health issues, the second most common reason for people to be out of work is, I'm not sure of the English word, but like back injuries, that's not right, but like having pain somewhere in your muscles, like having having back issues or neck issues or soreness that stops you from being able-.

**Interviewer** 24:02

'Chronic', is that the word?

**Participant 12** 24:02

Yeah, chronic is probably, yeah.

**Interviewer** 24:05

So then, what are your thoughts on something like what I've described being used in [company] products? Do you think there's scope for its usage?

**Participant 12** 24:17

I think there could be, and the thing with chronic injuries like this, it's not just about being away from work, it's being in pain, and a lot of times within healthcare, the pain is treated with a painkiller or something else, but the reason for the pain is never treated or never even found. That, "Okay, you have a sore back, take painkillers and rest". Okay, fine, but that doesn't-. I think that would appeal on quite a broad level to people, to be pain free.

**Interviewer** 24:46

Yeah. Okay, so then that's another element, then, as well to moving well, not just for the performance aspect, but it comes back to that injury-, not even just avoiding getting injured, but even once you are injured, there's that way of getting out of being in pain. Finally, then, do you have any other thoughts or comments related to this at all that you think might be useful?

**Participant 12** 25:14

This is something we talk about a lot that would be really useful to get the feedback. For instance, if I run quite a lot, and a lot of time when I run I am thinking that, "Is my technique correct?", and really, the only way to get any answer to that would be to have a running coach come and watch me run. And having a personal trainer for it, and like you said, for a lot of people that is not financially, or for other reasons, an option. So to be able to get some kind of feedback from a wearable saying that, even something as simple as cadence saying that, "Okay, your cadence is now too high" when you're running like, you know, "Takes shorter steps". Even simple things like this, that the devices actually already measure, but we don't necessarily give any feedback on. It's just a number that's in there. You can see what your cadence is, but we don't say. And I don't really know, should it be higher or lower or-? Sorry, now I went a bit off, but I think, for me, at least personally, I would find that quite useful to get that feedback.

**Interviewer** 26:19

Alright. Yeah, that's the end.

**Participant 12** 26:25

Alright.
