# Supplementary material for: Assessing and Enhancing Movement Quality Using Wearables and Consumer Technologies: Thematic Analysis of Expert Perspectives
Source: JMIR Form Res. 2024 Sep 13;8:e56784. doi: 10.2196/56784 (PMC11437222; doi:10.2196/56784)

**Supplementary Information 2 – Thematic maps to assist in the generation of themes**

*Phase 3 – Generating initial themes*
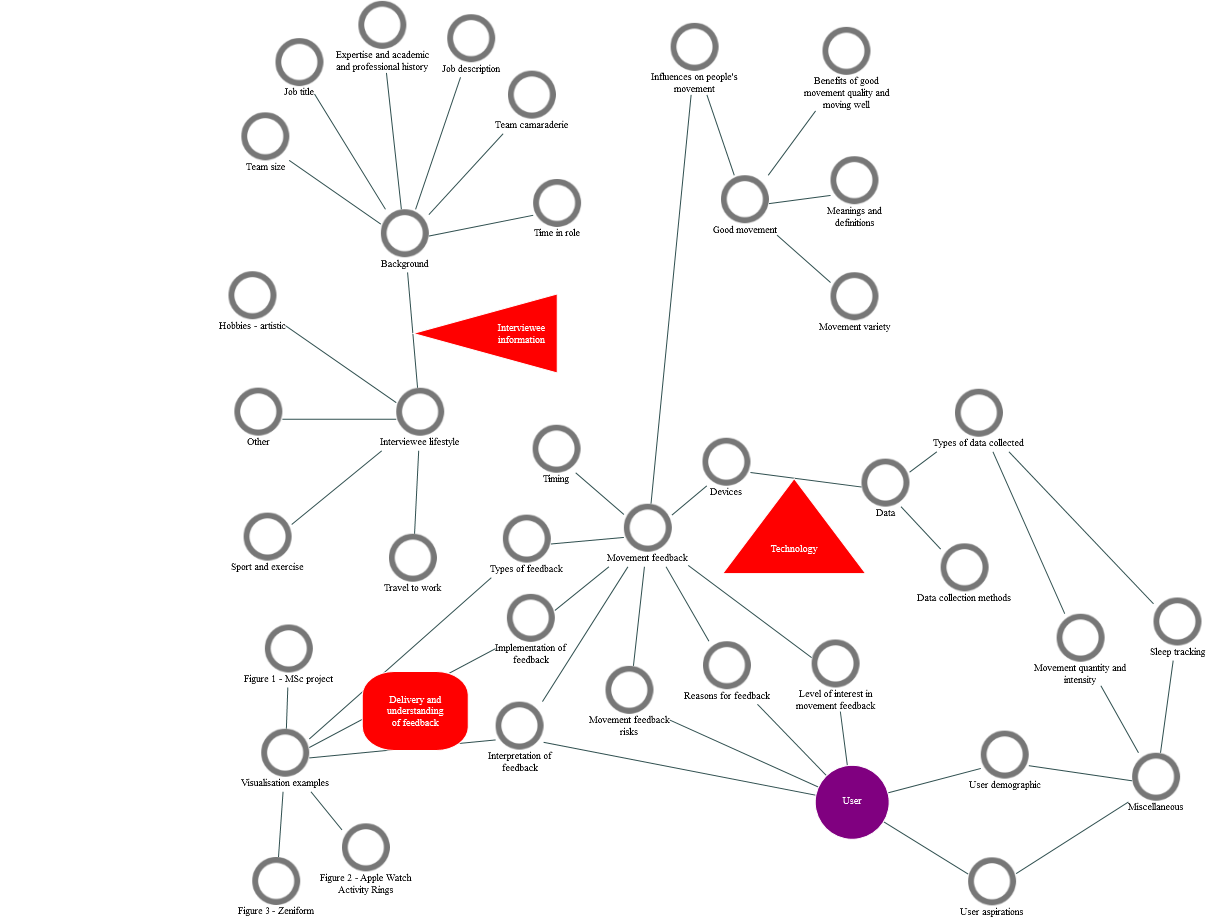


*Phase 4 – Developing and reviewing themes*


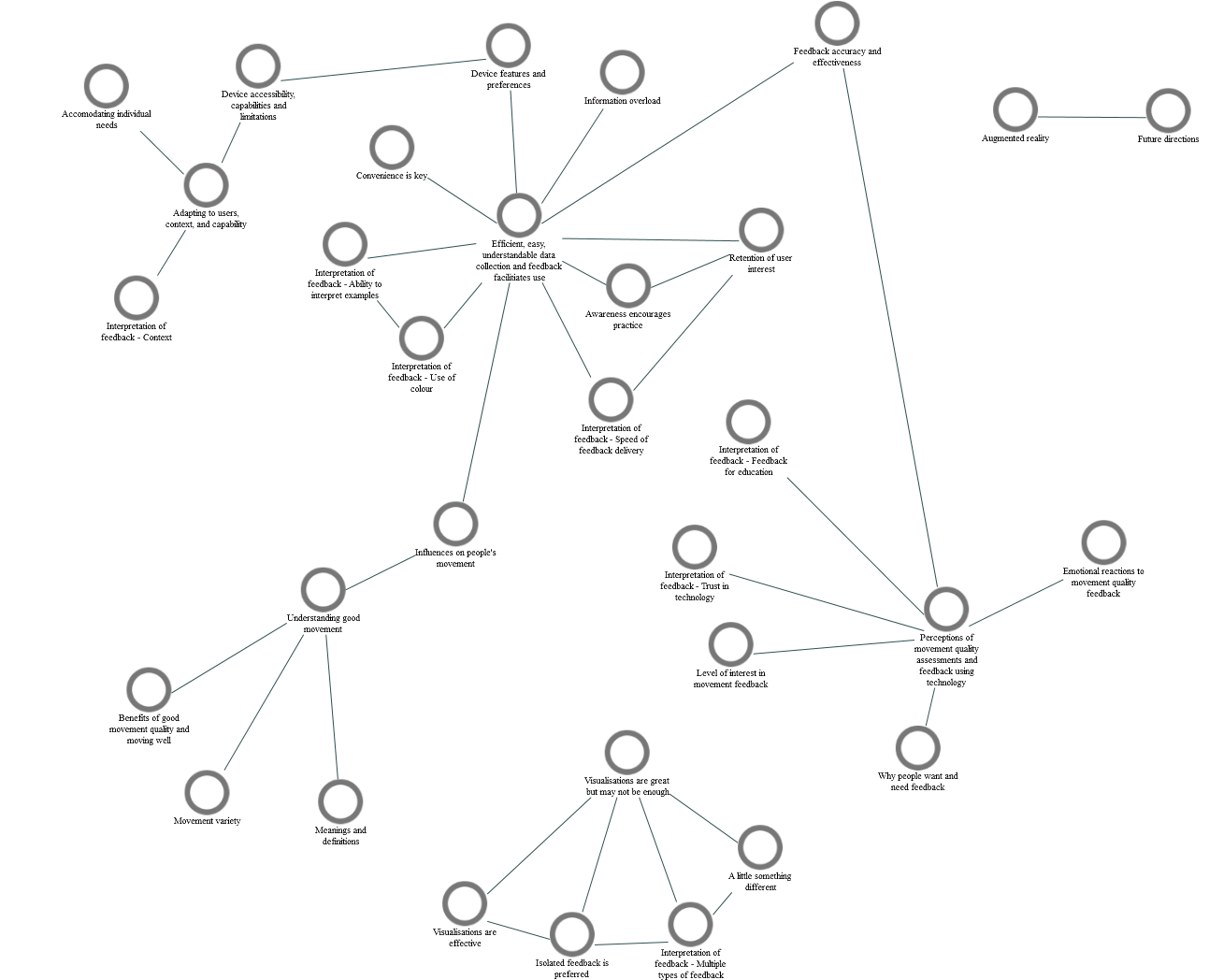


*Phase 5 – Refining, defining and preliminary naming of themes*


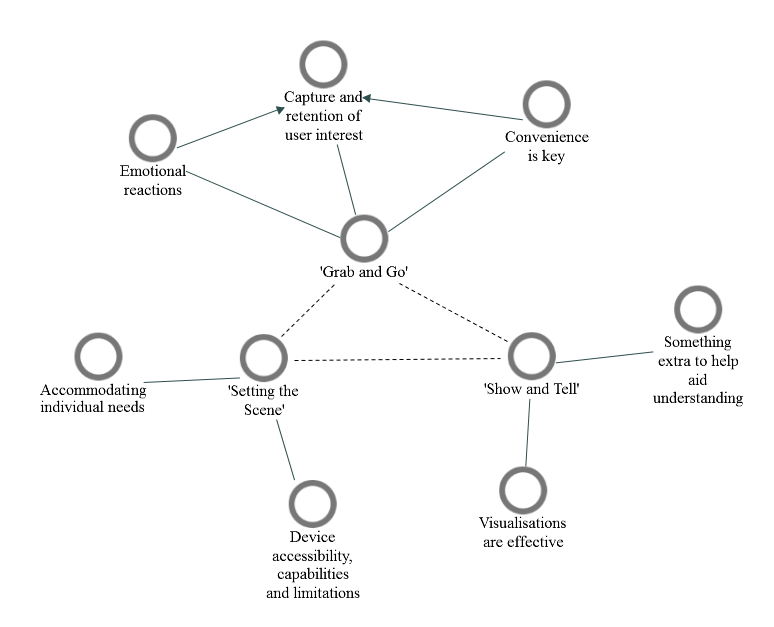

Supplement: Multimedia Appendix 4 [file formative_v8i1e56784_app4.doc]
